# Methodologies for Transcript Profiling Using Long-Read Technologies

**DOI:** 10.3389/fgene.2020.00606

**Published:** 2020-07-07

**Authors:** Spyros Oikonomopoulos, Anthony Bayega, Somayyeh Fahiminiya, Haig Djambazian, Pierre Berube, Jiannis Ragoussis

**Affiliations:** ^1^McGill Genome Centre, Department of Human Genetics, McGill University, Montréal, QC, Canada; ^2^Department of Bioengineering, McGill University, Montréal, QC, Canada

**Keywords:** RNA-Seq, long read, PacBio, nanopore, next-generation sequencing, transcriptome

## Abstract

RNA sequencing using next-generation sequencing technologies (NGS) is currently the standard approach for gene expression profiling, particularly for large-scale high-throughput studies. NGS technologies comprise high throughput, cost efficient short-read RNA-Seq, while emerging single molecule, long-read RNA-Seq technologies have enabled new approaches to study the transcriptome and its function. The emerging single molecule, long-read technologies are currently commercially available by Pacific Biosciences (PacBio) and Oxford Nanopore Technologies (ONT), while new methodologies based on short-read sequencing approaches are also being developed in order to provide long range single molecule level information—for example, the ones represented by the 10x Genomics linked read methodology. The shift toward long-read sequencing technologies for transcriptome characterization is based on current increases in throughput and decreases in cost, making these attractive for *de novo* transcriptome assembly, isoform expression quantification, and in-depth RNA species analysis. These types of analyses were challenging with standard short sequencing approaches, due to the complex nature of the transcriptome, which consists of variable lengths of transcripts and multiple alternatively spliced isoforms for most genes, as well as the high sequence similarity of highly abundant species of RNA, such as rRNAs. Here we aim to focus on single molecule level sequencing technologies and single-cell technologies that, combined with perturbation tools, allow the analysis of complete RNA species, whether short or long, at high resolution. In parallel, these tools have opened new ways in understanding gene functions at the tissue, network, and pathway levels, as well as their detailed functional characterization. Analysis of the epi-transcriptome, including RNA methylation and modification and the effects of such modifications on biological systems is now enabled through direct RNA sequencing instead of classical indirect approaches. However, many difficulties and challenges remain, such as methodologies to generate full-length RNA or cDNA libraries from all different species of RNAs, not only poly-A containing transcripts, and the identification of allele-specific transcripts due to current error rates of single molecule technologies, while the bioinformatics analysis on long-read data for accurate identification of 5′ and 3′ UTRs is still in development.

## Introduction

RNA sequencing (RNA-Seq) using short-read sequencing technologies currently offered by Illumina or Thermo Fisher (Ion Torrent) represents the standard and widely used method for transcriptome profiling (Goodwin et al., 2016). Recently, another sequencing technology from MGI (DNBSEQ), which is based on the formation of DNA nanoballs (Huang et al., 2017), has been used for RNA-seq studies and has shown a comparable performance in terms of quantification of gene expression and technical variability to the Illumina platform (Jeon et al., 2019; Natarajan et al., 2019). Short-read sequencing can produce reads of up to 300 bp (Illumina), 400 bp (MGI), or 600 bp (Ion Torrent), while paired-end sequencing (where library inserts are sequenced from both ends), whenever available, can effectively double the amount of sequence obtainable from a given DNA fragment. Short-read RNA-Seq requires either the RNA to be first fragmented and then reverse-transcribed or full-length cDNAs to be fragmented to create sequencing ready libraries with a mean length of 300 bp (typically ranging 200–700 bp). Since most mammalian mRNA transcripts are 1–2 kb in length (Harrow et al., 2012; Sharon et al., 2013), with the longest processed human transcript known, Titin, spanning > 100 kb (Bang et al., 2001), obtaining complete mRNA sequence information relies either on alignment to annotated genomic or transcriptomic sequences or *de novo* transcriptome assembly approaches. Furthermore, the complexity of the transcriptome is increased by the fact that most genes possess more than one transcriptional isoform (Gustincich et al., 2006). These isoforms are defined as mRNA molecules transcribed from the same locus, as mRNAs can be produced from different transcriptional start sites, terminated at different polyadenylation sites, or as a consequence of alternative splicing (Black, 2003; Matlin et al., 2005). Reconstructing all expressed isoforms for each gene and quantifying the expression of all the isoforms is difficult with currently available bioinformatics tools due to the limitations of short-read sequencing (Engstrom et al., 2013; Steijger et al., 2013). Therefore, long-read technologies represent a very powerful approach to unravel the full spectrum of gene expression profiles.

Currently, Pacific Biosciences (PacBio) and Oxford Nanopore Technologies (ONT) provide the most widely used long-read sequencing technologies. Read lengths achieved with these technologies (∼15 kb for PacBio and > 30 kb for ONT) surpass lengths of most transcripts. A relatively high cost and other limitations discussed here have prevented the wide use of these technologies in RNA-Seq, but now lower cost is promised through high-throughput nanopore sequencing approaches (currently represented from the ONT GridION and PromethION instruments), as well as the next generation of PacBio Sequel instrument (Sequel 2). For example, the per base sequencing cost on the PromethION platform can be as low as half the corresponding one on the MinION platform. In addition, the portability of the MinION instrument enables the sequencing of virus derived cDNAs and the identification of pathogens in the field (Castro-Wallace et al., 2017; Faria et al., 2017).

Long-read technologies combined with advances in full-length cDNA synthesis (Hawkins et al., 2003; Ramskold et al., 2012; Freeman, 2013; Cartolano et al., 2016), particularly SMARTer^TM^ (Switching Mechanism At RNA Termini) technology, commercially available from Clontech (United States), makes full length mRNA sequencing achievable with the added benefits of more accurate transcriptomic studies (Sharon et al., 2013; Oikonomopoulos et al., 2016; Byrne et al., 2017). PacBio provides the “Iso-Seq” workflow with protocols for all steps from library preparation to data analysis and viewing, while ONT provides PCR and PCR-free cDNA workflows that include library preparation protocols and data analysis guides and software. In this review, we will introduce the two currently available single-molecule, long-read technologies, followed by developments in cDNA synthesis and preparation of RNA molecules for sequencing.

## Analytical Aims of Long-Read mRNA Sequencing

There are seven main approaches underlying long-read mRNA sequencing, which lead to characterization and/or relative abundance quantification of transcripts that are already known or need to be discovered. (i) The first approach concerns the quantification of already known gene and isoform models. (ii) The second approach deals with the quantification of already known gene or isoform models, as well as the quantification of transcripts derived from novel isoform models (Abdel-Ghany et al., 2016). In this case, based on alignment data, a novel isoform can be characterized and assigned to an already-known gene. Additionally, the exon structure of novel genes can also be identified. (iii) The third approach involves only the characterization of the different isoforms, but not their quantification. Pioneered by the PacBio “Iso-Seq” method, this approach involves mainly the characterization of the different isoform models by sequencing groups of cDNA reads after fractionating them based on their length (Au et al., 2013). The process of size fractionation is adaptable to any long-read sequencing technology. (iv) The fourth approach involves the quantification (Dougherty et al., 2018) and/or exon structure characterization (Chen et al., 2017) of paralogous genes. (v) The fifth approach involves the identification of fusion transcripts and their corresponding chromosomal translocations (Nattestad et al., 2018). (vi) The sixth approach involves the identification of allele-specific expression (Tilgner et al., 2014) through haplotype phasing as well as the characterization of compound mutations (Cavelier et al., 2015). (vii) The seventh approach involves the identification of degradation patterns of specific transcripts (Ibrahim et al., 2018) or the identification of the native RNA processing patterns (Oesterreich et al., 2016).

## Properties of RNA Molecules and Library Preparation Strategies for Long-Read Sequencing

The aim of the following paragraphs is to present full-length cDNA synthesis strategies for long RNA molecules. Total RNA includes unprocessed transcripts, processed transcripts, and degradation products. Although the processed transcripts are the largest fraction of RNA molecules, the long-read sequencing platforms have been used to investigate all the different classes of RNA molecules. Below, we will first present the properties of the long-read RNA molecules that can be used to differentiate between the different categories. Next, we will present the library preparation methods that are exploiting these properties to specifically enrich for the different categories.

### Properties of Long RNA Molecules and RNA Fragments

Depending on the type of the RNA/cDNA library different features of the RNA molecules can be exploited to specifically enrich for the targeted population. These features rely either on the sequence content (presence or absence of a poly-A tail) or on the 5′ and 3′ end moieties present at the end of the molecules. The 5′ moieties correspond to the chemical entities attached on the fifth carbon (5′-C) of the sugar ring of the first nucleotide (phosphate, tri-phosphate, and hydroxyl groups). The 3′ moieties correspond to the chemical entities attached on the third carbon (3′-C; phosphate, and hydroxyl groups) or the second (2′-C) carbon of the sugar ring of the last nucleotide. Nucleotides that have different chemical moieties on the second carbon of the sugar ring, except the hydroxyl group, are part of the group of modified bases. Depending on the chemical moieties of the fifth carbon of the sugar ring of the first nucleotide and on the chemical moieties of the third carbon of the sugar ring of the last nucleotide as well as the presence or not of a poly-A tail and of the m^7^G cap, the RNA molecules can be grouped in the following three categories (Supplementary Figure 1).

In the first category belong the capped (m^7^G) RNA molecules. In this category, the RNA molecules correspond to full-length molecules (poly-A and non-poly-A molecules). Additionally, in this category, we can find RNA fragments that derive from either 3′ exonucleolytic degradation or endonucleolytic degradation of full-length RNA molecules. 3′–5′ exonucleolytic cleavage can occur, for example, from the Rrp44 ribonuclease of the exosome complex (Schoenberg and Maquat, 2012). Endonucleolytic cleavage can occur from the Smg6 ribonuclease part of a complex involved in the Nonsense-mediated mRNA decay pathway (Schoenberg and Maquat, 2012) or from the unknown protein Ribothrypsin (Ibrahim et al., 2018). Both can create capped RNA fragments with 3′-OH ends.

In the second category belong the uncapped RNA molecules. In this category, we can find uncapped RNA molecules with a 5′-PPP—for example, the prokaryotic transcripts (Sorek and Cossart, 2010). Additionally, in this category we can find uncapped RNA molecules with a 5′-P. These last fragments are derived from uncapped molecules with a 5′-PPP that are initially recognized from a pyrophosphohydrolase to create 5′-P molecules like the RppH (Hui et al., 2014). Endonucleolytic cleavage, for example, by the *B. subtilis* RNAse Y (Shahbabian et al., 2009), the *B. subtilis* RNAse J1 (Zhao et al., 2015), or the *E. coli* RNAse E (Kushner, 2002) can create RNA fragments with a 5′-PPP and a 3′-OH.

In the third category belong the uncapped RNA fragments. In this category we can find RNA fragments derived from uncapped RNA molecules with a 5′-P, as presented above. Afterward, these 5′-P molecules are recognized from RNA exonucleases to create shorter 5′-P degradation fragments. Additionally, in this category, we can find RNA fragments derived from endonucleolytic degradation of RNA. Endonucleolytic cleavage, for example, by the *B. subtilis* RNAse Y (Shahbabian et al., 2009), the *B. subtilis* RNAse J1 (Zhao et al., 2015), or the *E. coli* RNAse E (Kushner, 2002) can create RNA fragments with 5′-P ends and 3′-OH ends, in addition to the already mentioned RNA fragments with 5′-PPP and 3′-OH ends. Other endonucleases, like the secreted ribonucleases of the vertebrate-specific RNaseA superfamily (e.g., the multi-purpose RNAse 1 Lu et al., 2018), the tRNA splicing endonuclease (Trotta et al., 1997), the Ire1 (Sidrauski and Walter, 1997), the RNase T2 (Luhtala and Parker, 2010), and the RNase L (Cooper et al., 2014), can create RNA fragments with 5′-OH and 3′-P (Cuchillo et al., 2011). In the last case these 5′-OH and 3′-P are in small abundance in the cells. All these fragments can be sequentially degraded from exonucleases. For example, the ribonuclease XRN1 degrades RNAs bearing 5′-P (Schoenberg and Maquat, 2012).

Based on the above we have five types of RNA molecules (Supplementary Figure 1). (i) The first type is the capped and polyadenylated RNA molecules with 3′-OH. (ii) The second type is the uncapped and non-polyadenylated RNA molecules with 5′-PPP and 3′-OH. (iii) The third type is the capped and non-polyadenylated RNA molecules with 3′-OH. (iv) The fourth type is the RNA fragments with 5′-P and 3′-OH. (v) The fifth type is the RNA fragments with 5′-OH and 3′-P.

### Strategies for cDNA Synthesis Using RNA Molecules and RNA Fragments

Depending on the library preparation method used, all or a fraction of the molecules from the five categories presented in the previous paragraph can be sequenced. In general, the sequence properties of the RNA molecules of interest are exploited by hybridizing probes on the sequences of interest (for example poly-T probes against the poly-A sequences). Then, the RNA molecules can be either isolated with the help of these probes bound on streptavidin beads, or these probes can be used to prime reverse transcription (Supplementary Figure 1). Alternatively, the hybridized probes can block the reverse transcription of the targeted sequences [for example probes against the globin mRNAs; Globin Block Module from Lexogen (Austria)]. Exploiting the properties of the 5′ and 3′ ends relies on the ligation efficiency of the commonly used T4 RNA ligase 1 and the truncated T4 RNA ligase 2 (Supplementary Figure 1). For example, the m^7^G of the mature mRNA molecules prevents the ligation of adaptors on the 5′ end by the T4 RNA ligase 1. The T4 RNA ligase 1 can add an RNA adaptor only in the presence of 5′-P RNA molecules and not in the presence of a 5′-OH. The truncated T4 RNA ligase 2 can add a ssDNA adaptor only in the presence of 3′-OH RNA molecules. The presence of 3′-P inhibits the ligation of the adaptor with the truncated T4 RNA ligase 2 enzyme. In other methods, the small amounts of RNAs bearing 5′-OH termini can be ligated to the 5′ adaptors by using a RtcB RNA ligase (NEB) (Peach et al., 2015) (Supplementary Figure 1).

To sequence a specific subpopulation of RNA molecules, we can use either a method that targets a specific feature of this population (feature selection method) or a method that targets a feature of the non-targeted population which will eventually exclude it from the pool of RNAs (feature exclusion method). An example of the feature selection method is the standard reverse transcription from a poly-T primer. In this case, molecules with a poly-A tail can be specifically enriched with a poly-T reverse transcription primer and a Template Switching protocol, with a reverse transcriptase that shows a capped-dependent terminal transferase activity [SMARTer (Sharon et al., 2013; Oikonomopoulos et al., 2016; Byrne et al., 2017); Supplementary Figure 1]. A variation of this method is a protocol where the poly-A RNA molecules are first pooled down from the pool of RNA molecules with poly-T hybridization probes and then are reverse transcribed using either poly-dT or degenerate reverse transcription primers.

Examples of the feature exclusion method are the following. There is the case of the removal of unwanted ribosomal sequences that can be performed with subtraction hybridization with rRNA-specific probes. Similarly, highly abundant transcripts (for example globin) can be removed with subtraction hybridization with gene specific probes or can be blocked from reverse transcription through hybridization with gene specific sequences [for example in the case of globin the GlobinClear kit from ThermoFisher and the GlobinLock (Krjutskov et al., 2016) method, respectively]. In another case, capped mRNAs can be enriched through digestion with Terminator 5′ phosphate (P)-dependent exonuclease (Ibrahim et al., 2018) a procedure that will remove RNA fragments starting with a 5′-P (Supplementary Figure 1). Additionally, the RNA fragments with a 5′-OH terminus can be removed after T4 Polynucleotide Kinase (PNK, NEB) treatment, followed by Terminator 5′ phosphate (P)-dependent exonuclease (Ibrahim et al., 2018) (Supplementary Figure 1). In the feature exclusion methods, we can also include non-enzymatic approaches like gel-size selection. If present during cDNA library synthesis, the cDNA synthesized from small, degradation-derived, RNA fragments can be removed after gel-size selection of cDNA molecules (BluePippin; Sage Science, United States) by isolating molecules that are, for example, greater than 500 bp. Similarly, size selection of the cDNA molecules can be achieved with the bead based cleanup steps (SPRI Magnetic Beads; Applied Biological Materials Inc., United States) of the synthesized cDNA. Additionally, depending on the RNA extraction kit, the small RNA fragments can be removed during RNA extraction through RNA purification columns. For example, RNA molecules with a size < 200 nucleotides are removed with the RNeasy Mini Kit (Qiagen -United States).

Usually, the protocols have a combination of feature selection and feature exclusion methods. For example, the following approach aims to sequence capped mRNA molecules in a poly-A independent manner. There is an interest to use this method because the poly-A length and the polyadenylation levels themselves can vary, as shown in developmental processes (Owens et al., 2016). In this case a total RNA-Seq method is combined with a cap-enrichment methodology. To create a full-length cDNA from a ribosomal depleted total RNA-Seq, two alternative methods can be followed. In one method an adaptor is ligated on the 3′ end of the RNA molecules, which is followed by priming the cDNA synthesis from this adaptor [a similar approach to the Akron-SMRT (Ibrahim et al., 2018) and the SMIT-seq (Oesterreich et al., 2016) protocols; Supplementary Figure 1]. In another method, poly-A tailing of the RNA molecules is first performed using the *E. coli* Poly (A) Polymerase (NEB), which is then followed by priming the cDNA synthesis with a poly-dT sequence bound on the poly-A tailed region [a similar approach to the SMRT-Cappable-seq protocol (Yan et al., 2018)].

In general, after isolating the total-RNA, one of the following three cap-enrichment methods can be used. The first method is an enzymatic based capped mRNA molecules selection. This method includes all the template switching protocols, where the reverse transcriptase shows a capped-dependent terminal transferase activity—for example, the NanoCAGE protocol (Salimullah et al., 2011). The second method is a 5′ adaptor based capped mRNA molecules selection. Here, one can find the “TeloPrime Full-Length cDNA Amplification Kit” from Lexogen (Austria). The third method involves the depletion of uncapped mRNA molecule. As already mentioned, capped mRNAs can be enriched through digestion with Terminator 5′ phosphate (P)-dependent exonuclease (Ibrahim et al., 2018) (Supplementary Figure 1).

For the RNA fragments, as there is an interest to define precisely the beginning or the end of the fragments, an adaptor ligation technique is usually followed (Supplementary Figure 1). The approach is similar with the sequencing of the small RNAs on the Illumina platforms where adaptors are ligated at the end of the small RNA molecules. As already mentioned, the ligation of the library preparation adaptors is performed with the truncated T4 RNA Ligase 2 and the T4 RNA Ligase 1 for the 3′ end and the 5′ or 3′ end respectively (Dard-Dascot et al., 2018). For example 5′ end adaptor ligation with the T4 RNA Ligase 1 is used in the Akron5-seq protocol (Ibrahim et al., 2018), 3′ end adaptor ligation with the T4 RNA Ligase 1 is used in the Akron-SMRT protocol and 3′ end adaptor ligation with the truncated T4 RNA Ligase 2 is used in the SMIT-seq protocol (Supplementary Figure 1).

We also note that potentially modified nucleotides present at the end of the RNA fragments can affect the ligation efficiency of the adaptors. For example, in the plant miRNAs, in the piRNAs in insects nematodes and mammals, and in the siRNAs in insects and plants, the 3′ terminal nucleotide carries a 2′-O-methyl modification (Ghildiyal and Zamore, 2009). This modification strongly reduces the efficiency of 3′ adapter ligation (Munafo and Robb, 2010) on the RNA through the truncated T4 RNA Ligase 2. This differential ligation reaction can be used to negatively select a population.

### cDNA Synthesis

**FIGURE 1:**
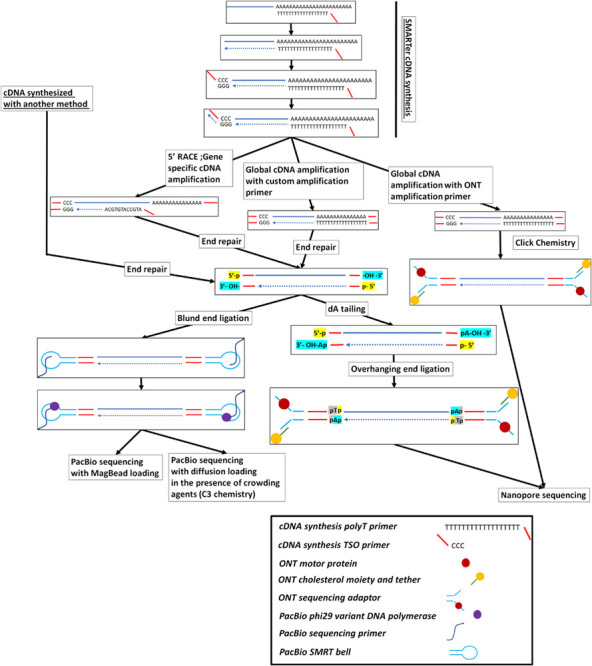
Full-length cDNA synthesis and long-read cDNA library preparation and sequencing on either the PacBio or the ONT platforms. The SMARTer cDNA synthesis method is presented here but other cDNA synthesis methods, like the ones presented in Supplementary Figure 1, can be used to create full-length cDNA from RNA molecules or RNA fragments.

Currently, there are two protocols for performing cDNA synthesis; cDNA synthesis from extracted RNA, typically using 10^5^ to 10^7^cells, or by direct lysis of 100 to 1,000 cells, followed by reverse transcription. The most widely approach for cDNA synthesis is the highly sensitive Smart-seq2 protocol (Picelli et al., 2013), which uses template switching and preamplification (Figure 1). It utilizes a combination of custom reagents and kits and is similar to the methodology tailored to long-read sequencing we recently published (Bayega et al., 2018b). It is important to consider the reverse transcriptase used in cDNA synthesis. For example, the Moloney murine leukemia virus (M-MLV) reverse transcriptase and its variants used in the SMART-Seq method (Ramskold et al., 2012) have a preference for full-length cDNAs over truncated ones as a substrate for their terminal transferase activities. This full-length cDNA preference is due to the 5′-CAP-dependent addition of specifically three to four non-templated dCMP residues to the 3′ end of full-length cDNAs in the presence of manganese (Schmidt and Mueller, 1999). Novel variants with increased thermostability have progressively been engineered named SuperScript II (42°C), Expand-RT (42°C), SuperScript III (50°C–55°C), and SuperScript IV (50°C–55°C). We note here that the different properties of these enzymes, namely the thermostability, the processivity (defined here as the amount of RNA reversed transcribed into cDNA) and the “template switching” efficiency (defined here as the amount of cDNA molecules that are full-length) are independent from one another. Improvement in one of these enzymatic properties does not imply improvement in the rest of them. For example, Superscript II has been reported to be eight times more efficient in creating full-length cDNA molecules, compared to the more thermostable version SuperScript III (Zajac et al., 2013), either due to the increased processivity or the increased terminal transferase activity happening in the lower temperatures of SuperScript II polymerization conditions; even the thermostability engineering of SuperScript III can have a negative effect in the other two properties of the enzyme. Despite a potential negative effect in the processivity and the terminal transferase activity, the thermostability improvement is beneficial as higher temperatures enable better dissociation of RNA secondary structures, thus enabling synthesis of longer cDNA molecules than the ones that are usually synthesized from the non-thermostable enzymes (Myers and Gelfand, 1991; Bayega et al., 2018c). Processivity can differ between the different enzyme variants. In a study where nine variants of the M-MLV reverse transcriptase were tested with 1,000-fold different concentrations of input RNA (1–1,000 pgs), variable cDNA yield amounts were observed for the different transcriptases with Maxima H Minus (ThermoFisher), producing consistently the highest cDNA yield (Bagnoli et al., 2018). Another important issue is that reverse transcriptases can have a high error rate, so it is important to use high-fidelity versions (available by several vendors) that are engineered to reduce error rates (Boutabout et al., 2001; Arezi and Hogrefe, 2007).

### cDNA Synthesis Artifacts

All the reverse transcription methods suffer from limitations inherent to cDNA synthesis and amplification such as template switching (Cocquet et al., 2006), artifactual splicing (Roy and Irimia, 2008), loss of strandedness information (Haddad et al., 2007), obscuring of base modifications (Ebhardt et al., 2009), and propagation of error (Nordgard et al., 2006). Artifactual splicing is the result of template switching on highly similar sequences present on the same molecule. This problem can appear during cDNA synthesis and can be avoided using the direct RNA sequencing platforms. In the long-read sequencing platforms, the loss of strandedness information can only happen in non-polyA + RNA molecules when the adaptors at the 5′ end and the 3′ end are similar and not distinct conditional on the error rate of the platform. In general, for reads from polyA + RNAs where the full-length molecule is sequenced, the beginning and end of the transcripts can be discriminated from the presence of the polyA + sequence (Byrne et al., 2017). The loss of RNA base information is expected as the RNA information is copied into cDNA, and only the direct RNA sequencing platforms can address this problem, at least for the modified bases that can recognize.

The propagation of error is inherent in the nature of PCR as molecules are copied multiple times. This kind of error can be controlled if during the cDNA amplification the minimum number of PCR cycles is selected. Increased coverage per molecule type can buffer these errors. Alternatively, molecular barcoding during reverse transcription can alleviate this problem by collapsing molecules with the same molecular barcode [unique molecular identifiers (UMIs)] into the same consensus molecule (Salk et al., 2018). Nevertheless, this approach is not easily implemented, as in the long-read sequencing platforms the error rate obscures the accurate basecalling of the molecular indexes. Additionally, the template switching and cDNA amplification (PCR-mediated recombination) artifacts can reduce the confidence in the detection for example of low-frequency compound mutations (Parker et al., 2014). It is worth noting here that for the sequencing reaction itself, although both long-read sequencing technologies are amplification-free single molecule sensing technologies, the short-read sequencing platforms still do rely on amplification of the sequencing ready library for priming their sequencing flow cells. This has the consequence of introducing additional amplification errors on top of the errors introduced during the PCR based cDNA amplification reaction. For this, efforts have been made to move away from an exponential PCR amplification reaction, such as the one used during the priming of the Illumina flow cell [Exclusion Amplification Chemistry (ExAmp)] (Shen et al., 2016), into a linear amplification reaction [Rolling Circle Replication (RCR)], such as the one used for the creation of the DNA nanoballs on the MGI platforms (Drmanac et al., 2020). Compared to ExAmp, the RCR amplification avoids the exponential accumulation of errors in the same position (Drmanac et al., 2020).

In the case of the reverse transcription of the viral RNA genomes, another artifact can arise when more than three nucleotides of the MinION strand switching oligonucleotide are found on the viral genome, allowing both template switching and false priming. It has been shown that this can result in a large number of false-positive 5′ ends (Moldovan et al., 2018). TSS variation and RNA degradation need also to be considered when assessing the presence of novel TSSs (Moldovan et al., 2018). Additionally, false priming errors can contribute to the artifactual 3′ ends of the reads, where the oligo (dT) primers hybridize with homologous stretches of the transcripts, generally with a much lower affinity. Assessing the upstream genomic region can be used to filter these artifactual positions (Moldovan et al., 2018).

## Long-Read RNA Sequencing Methodologies

Below, we will initially present some characteristics of the long-read sequencing platforms followed by a description of the current platform specific library preparation methods. A summary of the information presented in the following sections, regarding the technical comparison between the PacBio and the ONT platforms, is provided in Supplementary Table 1.

### PacBio Long-Read Sequencing Platform Overview

For a complementary review, see An et al. (2018) and Rhoads and Au (2015). Pacific Biosciences developed a method for single molecule real-time sequencing (SMRT), based on capturing sequence information during the replication process of the target DNA molecule (Eid et al., 2009). The template consists of a circular DNA molecule, created by ligating hairpin adaptors to both ends of a double-stranded DNA, called a library of so-called SMRTbell molecules (Rhoads and Au, 2015) Figure 1; for details, see Bayega et al. (2018a). The library is bound through the hairpin adaptors to a heavily engineered variant of phi29 Polymerase at a 1:1 ratio, loaded on a special flowcell consisting of SMRT cells where it diffuses into sequencing units, called zero-mode waveguide microwells (ZMWs). ZMWs provide the smallest available volume for light detection (Levene et al., 2003). In each ZMW microwell, the polymerase is immobilized at the bottom, and the replication process starts (Eid et al., 2009). Four phospholinked fluorescent-labeled nucleotides, with distinct emission spectra, are added to the SMRT cell. As a base is incorporated by the polymerase, a light pulse is produced that identifies the base (Eid et al., 2009). The replication processes in all ZMWs of a SMRT cell are recorded by a “movie” of light pulses, and the pulses corresponding to each ZMW can be interpreted to be a sequence of bases [for a representative figure, see Ardui et al. (2018)]. As the instrument is a laser based optical detection system, the sequencing length depends on the photodamage incurred on the polymerase. Despite that the sequencing reaction takes place in a low-oxygen, high-nitrogen atmosphere compartment, the photo-oxidation incurred on the enzyme prevents the polymerization reaction to reach its theoretical maximum sequencing length in all the occupied ZMWs.

The first PacBio instrument to be launched was the RSII, followed recently by the Sequel, which carries several modifications and improvements of the original technology that result in higher throughput. The number of ZMWs increased from 300,000 microwells on the RSII system to 1,000,000 microwells (1M SMRT cell) on the Sequel and SMRT cells with 8,000,000 microwells are scheduled to be produced. In our experience, read lengths of 20 kb can be achieved, while individual reads can exceed 60 kb, and there have been reports of maximum polymerase read lengths of 92.7 kb (Nakano et al., 2017; Zhang et al., 2017).

The PacBio systems have the limitation that are best suited to operate within dedicated facilities and are currently far away from being developed as bench top versions. The long-read lengths achieved with this technology, coupled with the Iso-Seq RNA sequencing protocol discussed below and downstream data analysis pipelines developed by PacBio provides a powerful approach to RNA analysis.

### PacBio Platform Sequencing Loading Overview

Originally loading the prepared library on the flowcell revealed a bias toward most efficient loading on the ZMWs array of shorter molecules (Loomis et al., 2013). This problem initially was not deemed very important for size-selected fragmented DNA library preparations where the size distribution due to the fragmentation process is relatively sharp and there is a complete representation of the different DNA sequences around the center of the fragmentation distribution. Nevertheless, it can significantly affect the cDNA libraries where different types of cDNA molecules have different sizes. In this case, no fragmentation is performed, as the cDNA is sequenced full length. To circumvent this problem a bead-based loading procedure, termed MagBeads, was followed where the molecules are first immobilized on the surface of solid beads and are then loaded on the SMRT cell. With this approach, the loading of the molecules is proportional to their concentration and not to their length. A disadvantage of this method is that molecules less than 600 bp cannot be loaded on the flowcell, as they are too short to be immobilized on the bottom of the ZMW microwell once the beads are deposited on top of the microwell (Supplementary Figure 2). This selective elimination of short fragments (< 600 bp) will unavoidably lead to inability to detect the shorter transcripts and RNA isoforms (Oikonomopoulos et al., 2016). Another approach that was followed to alleviate the size bias problem of the platform for the RNA-Seq samples, was to fractionate, based on their length, the cDNA molecules before sequencing. This approach, called Iso-seq (Au et al., 2013), is based on tight selection, using automated fractionation systems (SageELF; Sage, United States), of groups of cDNA molecules where the cDNA molecules inside each group are of a similar size and the groups between them show a different average cDNA size length. Alternatively, recent improvements in the chemistry (v3.0 chemistry) of the loading buffer with the introduction of molecular crowding agents seems to alleviate, to some extent, the short-read loading bias, making obsolete the usage of a bead-based loading procedure (Figure 1).

### Analysis of the PacBio Sequenced Reads

PacBio provides “SMRT Analysis,” an open-source bioinformatics software suite, for the analysis of data from SMRT technology. Here, we will focus only in the analysis of RNA-Seq data. Due to the small size of RNA molecules and the circular nature of sequencing, the forward and reverse strand of the same molecules can be sequenced multiple times, and all of them exist sequentially in the same sequenced read. Each time one strand of the cDNA molecule is sequenced, we refer to this event as a “subpass” and the produced part of the sequenced read as “subread.” The complete analysis of PacBio RNA-Seq data can be divided into five main processes: read-of-insert generation and classification, clustering, polishing, alignment, and visualization. Read-of-insert (ROIs) generation involves determining the highest quality sequence for each ZMW microwell; this includes inserts with < 2 subpasses and circular-consensus sequence (CCS) reads generated from inserts with ≥ 2 full-pass subreads. In the first case, the highest quality read is the longest subread or the only subread available. In the second, case a consensus is created from the full-length subreads. The ROI is then classified into “full-length” (FL) if the PacBio adaptor sequences are detected in both the 3′ and 5′ ends of the subread/consensus along with a poly-A tail. Alternatively, the ROI is classified into “non-full-length” (NFL) if the PacBio adaptor sequences are absent from either the 3′ or 5′ end or from both ends. Subsequently, the FL reads are processed through the Iterative Clustering for Error Correction (ICE) algorithm to build consensus clusters of one type of molecules. Each FL read is assigned to only one cluster while each cluster (also called consensus isoform) comprise of one or more FL reads. NFL reads are used to increase the coverage of each ICE consensus isoform. With enough FL and NFL coverage, the Quiver algorithm (for PacBio RS II data) or the Arrow algorithm (for Sequel data) polishes consensus sequences yielding high-quality (HQ) (basecalling accuracy ≥ 99%) and low-quality (basecalling accuracy < 99%) sequences depending on the number of reads present in each cluster (Supplementary Figure 3). Finally, the HQ reads can be mapped to the reference genome using a splice-aware aligner such as GMAP (Wu and Watanabe, 2005), GraphMap (Krizanovic et al., 2017), Minimap2 (Li, 2018) or to transcript sequences using BLASR (Chaisson and Tesler, 2012). In some cases, BBMap (Bushnell, 2014) can perform quite well, especially with long-read sequencing data that have lower error rates (for example PacBio ROIs) as well as on simpler organisms with less multi-exonic genes (Krizanovic et al., 2018). Another aligner Magic-BLAST, when tested on PacBio data, showed alignment statistics close to Minimap2 (Li, 2018) and a good performance in intron discovery and in the precise identification of intron boundaries (Boratyn et al., 2019). To further explore the Iso-Seq data several tools have been developed, for instance, MatchAnnot (Skelley, 2015), IsoView (Jack, 2015), and IsoSeq-Browser (Hu et al., 2017) provide a complete visualization of long read isoforms.

### PacBio Sequel Performance

With the Sequel system v6.0 release and the sequencing chemistry v3.0, a 1M SMRT cell can produce on average ∼615,000 sequenced reads, with a sequencing yield of ∼7.69 Gb and an average sequencing size of ∼12.5 kb from a 10-h sequencing movie time (Supplementary Figure 4). The length of the sequencing movie depends on the average length of the sequenced molecules. With a processing rate of 2–3 bp per second a 10-h movie time corresponds to a maximum sequencing length of 70–100kb. Due to the circular nature of sequencing, this length is the sequencing length and not the length of the original cDNA molecules. It is expected that in a PacBio read of a given length, if it has been produced from a short cDNA isoform, the sequence of this short isoform will be present on the PacBio CCS read many more times than the sequence of a long cDNA isoform if the PacBio read has been produced from this long isoform.

### Nanopore Sequencing Platform Overview

Nanopore sequencing is a relatively new, single-molecule sequencing technology. Oxford Nanopore Technologies (ONT) has pioneered the development of nucleic acid sequencing using protein nanopores and commercialized its sequencing platform, the MinION, in 2014. The MinION is a miniaturized portable USB-powered device and the first commercially available protein nanopore sequencer. In contrast to all other commercially available sequencers to date, ONT technology determines the sequence of nucleic acids in a molecule directly without the need of amplification, sequencing by synthesis, or by ligation. A protein nanopore is inserted into a synthetic electrical resistant lipid membrane and immersed in an electrolyte solution. An electric field applied across the membrane drives DNA molecules through the nanopore while the current flow within the nanopore is recorded. The unique pattern of current disruption generated by the different nucleotides as the nucleic acid molecule is driven through the pore is used to determine the sequence of DNA. Nanopore sequencing technology currently generates the longest raw reads with no theoretical limits (Loman and Watson, 2015), a significant advantage in all genomics applications. The platform had multiple upgrades. The recent ones named 7.X, 9.X and 10.X, where the number before the dot corresponds to the type of protein nanopore used (for example E.coli CsgG pore for the release number 9) and the number after the dot to the version of the specific protein nanopore.

### Nanopore Platform Library Preparation Overview

RNA sequencing with ONT can either be performed via a cDNA synthesis step or by direct RNA sequencing. The cDNA molecules are end repaired, dA-tailed, and then ligated on adaptors with dT overhangs. In contrast to the PacBio, where the polymerase is loaded after the addition of the adaptors, here the adaptors have pre-bound the molecular motor. The molecular motor will then drive the 5′ end strand inside the nanopore and the nucleic acid strand will be sensed by the platform (for details see Bayega et al. (2018a). Recently, nanopore upgraded their sequencing adaptor ligation chemistry (v109 cDNA Sequencing Kit), which increased the sequencing adaptor ligation efficiency. In this case, the enzymatic, ligase-based, adaptor binding was replaced with click chemistry. As a result, the cDNA amplification is only performed with primers provided by the company as they have the chemical moieties that will permit them to ligate to the sequencing adaptors through click chemistry.

We, along with others, have shown that the ONT sequencing technique does not have a skew toward a specific read length, thus it can equally likely sequence shorter and longer full-length cDNAs compared to both Illumina and PacBio sequencing platforms (Oikonomopoulos et al., 2016; Weirather et al., 2017). To reduce variability in the cDNA yield, ONT currently recommends starting the cDNA synthesis from a polyA + -enriched RNA. The amount of polyA + RNA needed is decreased in every newer version of the library preparation chemistry and the starting polyA + amount is significantly lower once a cDNA synthesis protocol with a cDNA amplification step (∼1 ng polyA + RNA) is selected when compared with protocols where no cDNA amplification step takes place (direct cDNA sequencing; ∼100 ng polyA + RNA). Instead of using beads to pull down polyA + RNA, we have successfully synthesized and sequenced polyA + cDNA from total RNA (Bayega et al., 2018b). In this case, the number of cDNA amplification cycles was adjusted to achieve the expected cDNA yield (Bayega et al., 2018b). The starting material for the direct RNA sequencing kit is 500 ngs of polyA + RNA. According to the best performance metrics of ONT, for the “v109 sequencing chemistry” and the “R9.4.1 pore chemistry,” the corresponding yields on the MinION flow cell are 8 Gb in 48 h for either the amplified cDNA or the direct cDNA sequencing kits and 1–4 Gb in 48 h for the direct RNA sequencing kit. 30% of the total yield (2–3 Gb for either the amplified cDNA or direct cDNA sequencing kits and 1 Gb for the direct RNA sequencing kit) is acquired in the first 6 h of sequencing. In our hands, from five 48-h sequencing runs of amplified cDNA, we took on average 15.5 Gb, which corresponded to an average number of 16.2 million sequenced reads (Supplementary Figure 5). Additionally, from five 48-h direct RNA sequencing runs, we took on average 2.12 Gb, which corresponded to an average number of 1.96 million sequenced reads (Supplementary Figure 5). We expect in newer versions of the pore chemistry and sequencing chemistry that these performance metric will improve even more.

### Nanopore Data Analysis

In contrast to the circular nature of PacBio sequencing, the standard nanopore protocol does not permit the same molecules to be sequenced multiple times. Similarly, with the PacBio platform the reads can be grouped based on sequence similarity and a consensus can be produced that will increase the accuracy of the molecules. For example, the corresponding steps of alignment, clustering, and polishing can be performed as follows, with publicly available software. For classification, the reads can align with each other with the Minimap2 (Li, 2018) software and the alignment is filtered based on identity cut-off and length coverage. Then, clustering can be performed using either CARNAC (Marchet et al., 2018), isONclust (Sahlin and Medvedev, 2018), or even cd- hit (Li et al., 2017). This will create highly interconnected groups of reads where each read aligns with each other. Finally, in the polishing step, a consensus sequence can be created from reads present in the same cluster using pairwise partial order alignment (Byrne et al., 2017). Similar to PacBio, the reads can be error-corrected with short reads or corrected using the reference genomic sequence or self-corrected with the rest of the sequenced reads corresponding to the same transcript. In the absence of a reference genome, superTranscripts (Davidson et al., 2017) can be used to create a reference database where each gene will be uniquely presented as a concatenation of all its unique exonic sequences. Similarly, IDP-*de novo* (Fu et al., 2018) is another tool that can perform *de novo* transcriptome assembly, isoform annotation, and quantification by integrating information from both long reads and short reads when the reference genome is absent.

## Library Preparation Methodologies to Decrease the Error Rate of the Sequenced Reads Below the Raw Error Rate of the Individual Platforms

All the next-generation sequencing (NGS) technologies have some error rate during basecalling. Depending on the application, even the short-read sequencing technologies may have an unacceptable high error rate, as in, for example, the detection and quantification of rare variants/low frequency mutations among the different genome copies in heterogeneous mixtures of cells or molecules. The raw read error rate of the basecalling informatics for the PacBio Sequel is ∼85% (Ardui et al., 2018). In the case of the ONT, since 2014, the dramatic improvements in both the basecalling informatics (RNN instead of HMM basecalling) as well as the nanopore pore type and chemistry itself has led to an increase in accuracy from 60% in the initial versions to up to ∼90% in the current versions [reviewed in Rang et al. (2018)]. Although, currently it is not clear whether there is an inherent ceiling to either the nanopore read accuracy (Rang et al., 2018) or the ZMW read accuracy, variations of the conventional NGS protocols, along with the corresponding computational tools, have been developed that can improve the accuracy past the raw read error rate of the platform (for a review see Salk et al., 2018).

The PacBio consensus sequencing of single DNA molecules is one of these approaches. The circular library creation on PacBio can be used to sequence the same molecule multiple times, thus increasing the accuracy from 85% up to 99%, proportional to how many times either strand has been sequenced (Supplementary Figure 3). For example, the PacBio basecalling accuracy for the two read passes is 90% whereas three to four sequencing passes of the same molecule reaches an average accuracy of 95%. In its turn, ONT adopted a chemistry where both the forward and the reverse strand of a DNA molecule were sequenced, called 2D chemistry (Jain et al., 2016) in the earlier versions and currently called 1D^2^ chemistry. The nature of the nanopore sequencing platform does not permit to sequence the same molecule more than two times. The 2D chemistry permitted the sequencing of the two individual strands of a DNA molecule through the introduction of a hairpin in one end of the DNA molecule. In 1D^2^ chemistry, the hairpin molecule was abandoned and a time-based inference of the two complimentary strands was adopted. Although both these chemistries are significantly different in the way the sense and antisense strand of the DNA molecule are sequenced, they both can increase the accuracy by 5–15% (Rang et al., 2018) on top of the raw read accuracy.

**FIGURE 2:**
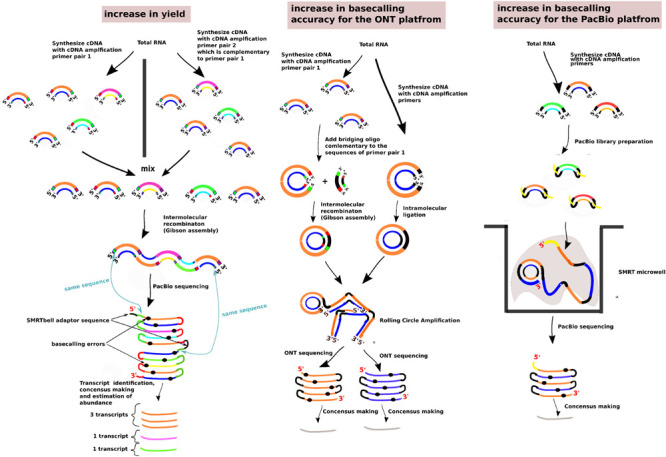
Methods to improve the sequencing yield and the basecalling accuracy past the specifications of the long-read sequencing platforms. The methods presented are the ConcatSeq (Schlecht et al., 2017), INC-seq (Li et al., 2016), R2C2 (Volden et al., 2018), and the PacBio Circular Consensus Sequencing. In the section “increase in yield,” the green boxes present at either the 5′ end or the 3′ end, depending on the pool of the cDNA molecules, indicate the same DNA sequence. Similarly, for the red boxes. The sequences represented from the green and red boxes are reverse complementary with each other. The same case is for the cDNA molecules and the bridging oligo in the “increase in basecalling accuracy for the ONT platform” section. In all the sections, the two strands of the cDNA molecules are indicated with different colors. Different cDNA molecules are indicated with a different pair of strand colors.

In the case of the ONT platform, two methodologies have been suggested to create linear concatemers of the individual sequenced cDNA molecules; these methodologies are called INC-seq (Li et al., 2016) and R2C2 (Volden et al., 2018; Figure 2). In INC-seq, intramolecular cDNA ligation with a T4 DNA ligase takes places and is followed by Rolling Circle Amplification with a phi29 DNA polymerase and random primers (Li et al., 2016). In R2C2, to circularize the cDNA molecules, a Gibson Assembly approach is followed where a DNA splinter joins the beginning and end of the molecules in the presence of an exonuclease, DNA polymerase, and DNAligase mix (NEBuilder HiFi DNA Assembly Master Mix). A Rolling Circle Amplification is followed with a phi29 DNA polymerase and random hexamers (Volden et al., 2018). As the rolling circle amplification can create structures with tree branches whose presence can block the nanopores, a T7 Endonuclease is being used that can debranch the RCA molecules (Volden et al., 2018). Similar with the PacBio Circular Consensus Sequencing approach, both these approaches for 1, 2–5, 6–10 and > 10 sequencing passes can increase the accuracy as follows, from 90.5%, 94.5%, 96.5% and up to 97.5%, respectively (Volden et al., 2018; Supplementary Figure 3).

To increase the accuracy further, a consensus creation approach is followed by overlapping similar but independently sequenced molecules. For example, in the case of PacBio, to increase the accuracy to 99.99%, approximately 15 sequencing passes of the target molecule are needed (Supplementary Figure 3), which can only be achieved from a mixture of multiple sequencing passes of the same molecule as well as of other similar molecules. Similarly, to reach a perfect consensus, each exon needs to be covered at least 50 times (Supplementary Figure 3). In the case of the RNA-Seq due to the large variety of isoforms as well as the different levels of expression, there will be exonic parts of the genes that are covered fewer than 50 times. In this case, further polishing of the sequence can only be achieved by merging high-quality short-read data with long-read data following the hybrid approach methods. However, in this case the biases present in the short-read data can be introduced in the long-read data ones.

## Direct RNA Sequencing Methodology

For a complementary review, see Marinov (2017) and Hussain (2018). As direct RNA sequencing, we describe the capability of the sequencing platform to directly sense the RNA strand of the RNA molecule. As indirect RNA sequencing, we describe the capability of the sequencing platform to indirectly sense the RNA strand of the RNA molecule through a cDNA intermediate. In 2009, a method for indirect RNA sequencing was developed on the Helicos Genetic Analysis System, a platform that was quickly phased out, where poly-A mRNA is sequenced by the step-wise synthesis of complementary first-strand cDNA and imaging of nucleotides labeled with an interfering but cleavable fluorescent dye (Ozsolak et al., 2009). The Helicos Genetic Analysis System was a short-read 32 bp sequencing system. In a similar way, RNA sequencing on the PacBio platform has been achieved by combining RNA molecules with a reverse transcriptase (Vilfan et al., 2013). Currently, a commercial kit for direct RNA sequencing method is restricted to the Oxford Nanopore platform and will be the focus of the review.

Similar to cDNA sequencing methodologies on the ONT platforms, the direct RNA sequencing methodology can identify and quantify splice variants (Workman et al., 2018). The ability to directly sequence RNA skips three main problems: First, there is no necessity to reverse transcribe the RNA into cDNA, a process that can introduce errors or biases in the resulting sequencing data (Lahens et al., 2014). Second, it permits the identification of RNA modifications as well as the poly-A tail length (currently for poly-A tails > 10bp). Third, and most important, for every different isoform detected, its fully processed (no introns present) or unprocessed (some introns present) status can be recorded along with its modifications and poly-A tail length (Workman et al., 2018).

It has already been shown that the ONT direct RNA sequencing approach can sequence some long and very long RNA molecules that are not efficiently synthesized into cDNAs (Workman et al., 2018). The identification of RNA modifications has biological implications and is based on the ability of the platform to sense the RNA modifications directly [see review from Novoa et al. (2017)]. The m^6^A in both *H. Sapiens* (Workman et al., 2018) and the *S. cerevisiae* (Garalde et al., 2018) has been shown to be detected from the ONT platform as well as the m^7^G modification in E. coli (Smith et al., 2017). Tombo (Stoiber et al., 2017) can be used to identify modified nucleotides from direct RNA raw nanopore sequencing data. Sensing modified nucleotides depends on the properties of the nanopore channel (Simpson et al., 2017) and as ONT has already changed the nanopore channel once and can change it again in the future, extra RNA modifications, potentially not currently detectable, can be sensed in the newer versions of the platform.

### Direct RNA Sequencing Library Preparation

**FIGURE 3:**
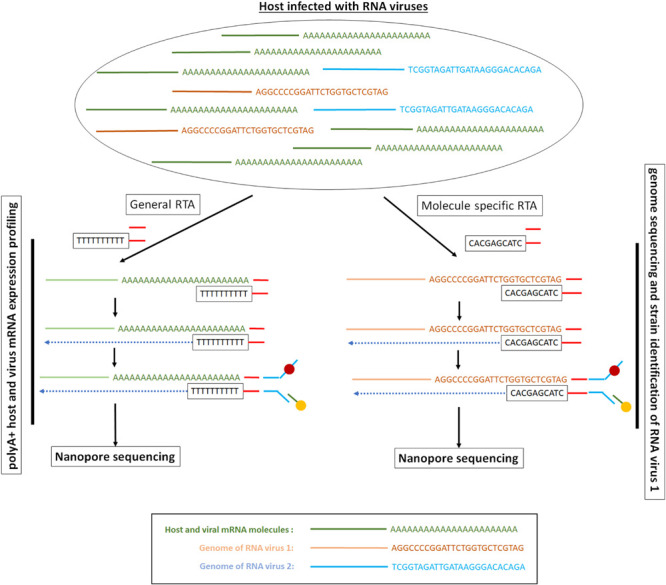
Long-read direct RNA library preparation methods for polyA + RNA sequencing or targeted RNA sequencing. The picture presents a hypothetical example where we have a mixture of polyA + mRNA molecules and viral RNA genomes. Depending on the RNA population of interest, the RTA adaptor can be generic and target all the mRNA molecules or can be specific and target only the viral RNA genomes. In the direct RNA sequencing method only the RNA strand goes through the ONT nanopore. Depending on the ONT protocol used, the RTA can serve as a priming location for reverse transcription of the entire length of the RNA molecule, though the cDNA strand that is generated is not sequenced.

The direct RNA sequencing method involves the sequential ligation of a reverse transcriptase adapter (RTA) and a sequencing adapter (Garalde et al., 2018; Figure 3). The RTA is a small dsDNA molecule that contains a T10 overhang designed to hybridize with poly-A mRNA and a 5′ phosphate (Pi) that ligates to the RNA creating a DNA–RNA hybrid. The RTA can serve as a priming location for reverse transcription of the entire length of the RNA molecule, though the cDNA generated is not sequenced. The DNA–RNA hybrid is then ligated to the sequencing adapter, which directs the RNA strand of the assembled library into the nanopore for sequencing (Garalde et al., 2018) for details see Bayega et al. (2018a). As there are only 10 thymidines as overhang, the adaptor can be used to assess the size of a poly-A mRNA tail at least 10 bp long (Workman et al., 2018).

The primary limitation of this technology is the high input material requirements, currently around 500 ngs of polyA + RNA. These RNA input requirements are not physically achievable with most original clinical samples and further improvements of the platform might reduce the input material. Another limitation is that without the presence of an extra adapter sequence at the 5′ end of the RNA molecule, there is a loss of coverage at the extreme 5′ end of the molecule, which is due to unreliable processivity as the last bases of the molecule pass through the pore, resulting in the loss of 10–15 nucleotides not being sequenced (Keller et al., 2018; Workman et al., 2018). Along the same lines with this remark is that with the direct RNA sequencing approach there is no selection for full-length RNA molecules. Indeed, molecules partially sequenced have been observed (Workman et al., 2018) and depending on the length of the mRNA, they can consist of up to 40% of total sequenced molecules (Workman et al., 2018). On the contrary, the SMART-seq protocol used during cDNA synthesis selects for full-length molecules, while assays for pull down of capped RNA with cap-binding proteins do exist (Bajak and Hagedorn, 2008).

Depending on how well trained the basecaller is for the targeted species along with potential species specific RNA modifications, the accuracy can be as good as the cDNA one for example the case of *H. sapiens* (Workman et al., 2018) or lower than this for example the case of Influenza (89% read-level accuracy for the cDNA versus 85% for the RNA sequenced reads Keller et al., 2018).

As the system is a two-ligation system based protocol, the RTA adaptors can be used to select a subset of RNA molecules conditional upon them having unique sequences at the end of the molecules, as has been done for the RNA genome of the Infuenza A virus (Keller et al., 2018; Figure 3). Although this might reduce the total sequencing yield, as the molecules of interest are a fraction of all available molecules, this targeted sequencing approach can enrich for the population of interest. However, this targeted sequencing of known or anticipated viruses, might not be optimal for samples with multiple viral strains and genotypes as well as for viruses cultured from clinical samples (Tan et al., 2018) where unknown viruses might be present and of significant interest. Alternatively, in order to obtain sufficient sequencing throughput, capture-based enrichment of the RNA molecules of interest can be performed, as demonstrated for viral RNA (Tan et al., 2018).

## Applications of Long-Read Sequencing Technologies

Single-molecule long-read technologies are being increasingly applied for transcriptomic studies (Supplementary Figure 6). These technologies provide new insights into the full-length sequence, alternative splicing, gene structure, and alternative polyadenylation sites. Long-read sequencing is an invaluable resource to capture the complexity of structural variation on both the genomic and transcriptomic levels, and a widespread adoption is expected as the costs further decline. The PacBio technology is now widely used for the characterization of cancer transcriptomes, where novel isoforms and fusion transcript expression detection is superior than short-read technologies. This approach surpasses mapping-based or assembly-based approaches. The effectiveness of MinION in accurate quantification of transcripts, in the detection of transcript variants and fused genes, in transcript based haplotype phasing and allele specific expression as well as single cell expression profiling has been shown in multiple studies, either jointly or separately as is presented in the next paragraph and in the supplementary text (Seki et al., 2019). In addition, full-length transcript sequence information is very useful for both genome annotation and gene function studies. Due to space limitations, we provide a detailed overview of the applications of long-read sequencing in the supplementary text. The reader is encouraged to read the supplementary text.

### Single Cell RNA-Seq With Long-Read Sequencing

Long-read technologies have been used in single-cell experiments. The two main features of the single-cell experiments are the barcoding of the individual mRNA molecules with Unique Molecular Identifiers (UMIs) and of the synthesized cDNA from each cell with a cell-specific barcode. Due to the high error rate of the long-read sequencing platforms, the PacBio CCS reads have been used to more confidently sequence either the UMIs or the cell-specific barcodes.

Demultiplexing of sequenced reads with UMIs can lead to better quantification of transcripts in single cells (Islam et al., 2014). As the UMIs are usually degenerate, in platforms with a high error rate of raw reads UMI identification is highly problematic. Accurate UMI demultiplexing has only being performed with the CCS reads of PacBio (Karlsson and Linnarsson, 2017). In this case, the CCS reads had an average Phred quality score of 40 (99.99%; 1 in 10,000 bases erroneous), which correspond to 12–13 sequencing passes of the same molecules (Supplementary Figure 3). This high-quality requirement is necessary, as only degenerate UMIs were used and not self-error-correcting UMIs. Nevertheless, CCS reads with 12–13 sequencing passes correspond to relatively short cDNA molecules and thus the long ones will be either absent or underrepresented. A similar PacBio CCS-based, UMI deconvolution approach, was used to calculate the abundance and the type of the different “Human Endogenous Retrovirus Type K” proviruses across five patient samples (Brinzevich et al., 2014). In this case, the authors sequenced cDNA amplicons of a highly divergent region of the viral RNA genome (Brinzevich et al., 2014).

Single-cell barcode identification was only performed after combining the 10x Genomics Chromium platform (abbreviated below as 10x) with PacBio in a method called ScISOr-Seq (Gupta et al., 2018). With this method, the full-length cDNAs produced from the 10x platform were subsequently sequenced on PacBio. In this case the Circular Consensus Read transcript reconstruction was followed again. Due to the high error rate and the fact that the error-corrected cell barcodes on 10x can only tolerate one mismatch, the authors predicted that the CCS PacBio reads will only be able to assign 60% of the reads in a given cell (Gupta et al., 2018). The authors did use the PacBio reads to infer the isoforms and they combined them with the 10x Genomics 3′ end quantification protocol to infer the abundance of isoforms based on the known 3′ end.

Similar with the approach above, single-cell cDNA synthesis with 10x has been combined with transcriptome profiling on the nanopore platform (Singh et al., 2018). For the nanopore library preparation the authors had, for each molecule, only one sequencing pass and, as mentioned, the 10x cell barcodes can only accommodate one sequencing error. For this reason, the authors focused on profiling only a few types of transcripts with nanopore, namely the B-cell and T-cell receptors through targeted cDNA capture and subsequent cDNA amplification. As the molecules differed mainly in the hypermutated regions and in the cell barcode region, the authors decided first to extract the detected cell barcodes from the short-read 10x scRNA-Seq data and then each nanopore read was assigned to a detected cell barcode after finding a perfect match of the cell barcode sequence in the first 200 bp or in the last 200 bp of each nanopore read (Singh et al., 2018).

A similar single-cell experiment was performed where the authors were trying to identify hypermutations on B-cell receptors (Volden et al., 2018). The difference with the previous experiment was that the single-cell cDNA barcoding was not based on a 10x platform but rather on a plate-based sequencing experiment. In this case no complex mix of single cell barcodes was used but rather due to the low throughput plate-based approach followed, the authors tagged cDNAs synthesized from different cells with the ONT multiplexing barcodes designed to accommodate the high error rate of the platform. Additionally, the authors did not rely on error-correcting the reads with short reads but rather they increased the accuracy of the nanopore reads with a multiple sequencing passes method like the R2C2 (Volden et al., 2018). Despite that, the authors used the R2C2 circularization protocol of nanopore reads, and their primers did contain degenerate sequences; no attempt for UMI deconvolution was performed.

In some of the above studies, the rational of using a long-read sequencing approach over a short-read sequencing one, for isoform identification and quantification, has already been commented in a recent review (Hardwick et al., 2019). From our side, we comment the following. Generally, in the single-cell studies the gene abundance quantification can be efficiently performed with the short-read sequencing of the 3′ end or the 5′ end cDNA fragments, as is employed in the protocols used in the droplet-based single-cell capture methods, as for example with the different protocols of the 10x Chromium platform (Salomon et al., 2019). Similarly, single-cell gene abundance quantification can be efficiently performed with the short-read sequencing of cDNA fragments that cover the full length of the cDNA molecules, as for example the Smart-seq2 method (Picelli et al., 2014) that uses a plate-based single-cell capture approach. Both these methods are adequate for gene expression quantification and no long-read sequencing is necessary unless the sequenced short fragments of the genes of interest do not align uniquely on the genome. If this is the case, longer reads are necessary, as for example the case of paralogous genes (see supplementary text for discussion on the advantages of long-read sequencing of highly similar genes). Indeed, in one of the studies mentioned in this paragraph, the authors (Brinzevich et al., 2014) used long-read sequencing because it permitted them to differentiate the abundance of 89 highly similar HERV-K pro-viruses that are integrated into the human genome by specifically sequencing as full length a 700 bp highly divergent part of their sequence.

The rest of the authors of the studies presented in this paragraph, additionally to gene expression quantification, they tried to quantify the different isoforms present in the single cells. Given that the isoform abundance reconstruction from full length short-read sequencing cDNA data can only give confident observations on differential exon abundance and not on the expression changes of the full length reconstructed isoforms, the authors correctly selected the full-length long-read sequencing approach to answer their biological question. For example, Karlsson and Linnarsson (2017) decided to profile the heterogeneity of isoforms among single cells from an oligodendrocyte population. This isoform heterogeneity includes alternative transcription start or end sites, novel splice junction,s and alternatively splicing patterns of non-constitutive exons. Similarly, Gupta et al. (2018) profiled the isoform heterogeneity among single cells in different cell types of the cerebellum, whereas Volden et al. (2018) identified isoforms of cell surface receptors in B-cells, some of which were of pharmacological importance.

Moving away from gene or isoform abundance quantification, another study presented in this paragraph used the long-read sequencing data to perform cell clonality studies. For example, Singh et al. (2018) performed targeted capture and sequencing of the full-length TCR and BCR receptors to match the T-cells and B-cells clonotypes between the primary tumor sites and their draining lymph nodes. This could have also been performed with short-read sequencing of the 5′ cDNA fragments corresponding to the V(D)J sequence part of the receptor cDNA, with protocols available on the 10x Chromium system. Furthermore, the authors (Singh et al., 2018) sequenced the full-length cDNA of the Igs and performed Ig isotype identification and quantification in naïve and memory B-cells present in a lymph node. The secreted or membrane bound status of these Igs was also elucidated. This approach necessitates the use of full-length long-read sequencing as it is the only way to pair the 5′ clonotype-specific V(D)J sequence of BCR or TCR transcripts with the different 3′ sequences for secreted or membrane forms of the different immunoglobulin isotypes (Singh et al., 2018). Overall, it is evident that some of the biological questions examined from the authors of the studies presented in this paragraph, could only be addressed with long-read sequencing. All these authors took into account the limitations of the long-read sequencing platforms regarding their error rates and throughput and they structured their experimental designs and interpretation accordingly. Further applications of long-read sequencing described in the supplementary text, and not in the studies presented here, can be used wherever appropriate in future single-cell studies.

## Read Coverage Equivalence Between the Short-Read and the Long-Read Technologies

**FIGURE 4:**
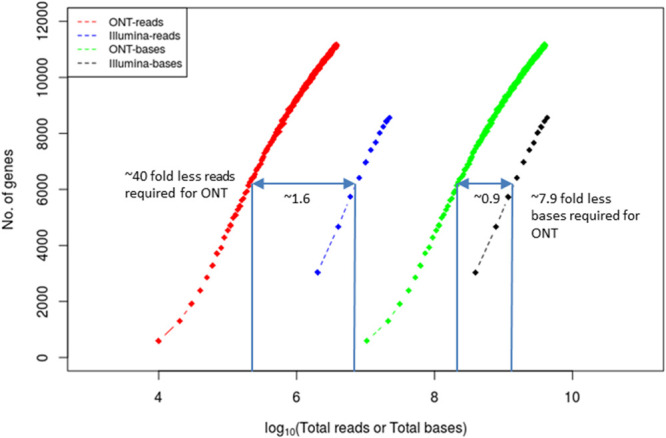
Coverage equivalence between short-read and long-read sequencing platforms. Number of cDNA reads and total number of bases required to detect the same number of genes (at 95% gene length coverage) in either the Illumina or the ONT MinION platforms. The *x*-axis and the indicated numbers on the plot are in log10 scale. The data used to derive the plot are from Bayega et al. (2018b).

Short-read and long-read sequencing technologies have obvious differences in read lengths and throughput and yet the number of reads is usually a critical factor in RNA-Seq applications and cost. Standards and expectations have already been established for short-read technologies. For example, for the accurate quantification of highly abundant genes (FPKM > 10) 36 million short-reads are needed whereas for the accurate quantification of low abundant genes (FPKM < 10), the corresponding sequencing depth can go up to 80 million short-reads (Sims et al., 2014). As expected, quantification of alternative splicing events requires more sequencing depth. Given that the spliced human genes have on average over nine transcribed isoforms at different abundances (Djebali et al., 2012), to detect alternative splicing events with 80% power, over 300 million reads are required (Consortium, 2014). It is important, therefore, to estimate how many long reads and short reads are needed to achieve the same goal. We performed Illumina short-read RNA-Seq and Oxford Nanopore long-read RNA-Seq on the same sample and performed rarefraction analysis (Figure 4). We determined that to detect the same number of genes (for example 6,000) with 95% coverage across the gene, and in comparison, to short reads, ∼40-fold fewer long reads and ∼8-fold less bases were required. This is expected since each long read routinely covers the entire length of the transcript unlike short reads.

## Toward Increasing the Number of Sequenced Molecules Beyond the Standard Yield of the Platforms

The number of molecules sequenced in PacBio is always proportional to the number of microwells present in the SMRT cell. As the rate of occupancy of the microwells follows a poisson distribution, there is always care to avoid overpopulating the SMRT cell with molecules that can lead to occupancy of the ZMW microwell from more than two molecules and a sequencing read that will either be badly basecalled or will have a random basecalled sequence. This means that trying to saturate the SMRT cell will significantly increase this problem. Re-engineering the ZMW microwells by introducing nanopores at the bottom has been proposed to alleviate this problem and offer a complete saturation of the SMRT cell (Larkin et al., 2017). Nevertheless, without re-engineering the SMRT cell, there have been studies that are exploiting the concatemerisation in the form of the ConcatSeq (Schlecht et al., 2017) protocol, where the same cDNA molecules are amplified in two separate reactions with two distinct sets of primers. The one primer set has complementary ends to the other primer set and after pooling the two cDNA populations together, a Gibson assembly reaction is followed where the ends of the two cDNA populations are recombining together, resulting in concatamers of the individual cDNA molecules (Figure 2). After PacBio library preparation and ZMW occupancy, not only one type of molecules will populate the SMRT cell but more than two depending on how many concatemerized molecules were present. The pitfall is that for the individual cDNA molecules, the sequencing subpass coverage from the CCS reads will be lower than in the conventional protocol, but the advantage is that after bioinformatically separating the concatemerized molecules the platform will have a 2× to 3× times more yield of the different types of cDNA molecules.

## Application of Linked Read Technology in Full-Length cDNA Sequencing

**FIGURE 5:**
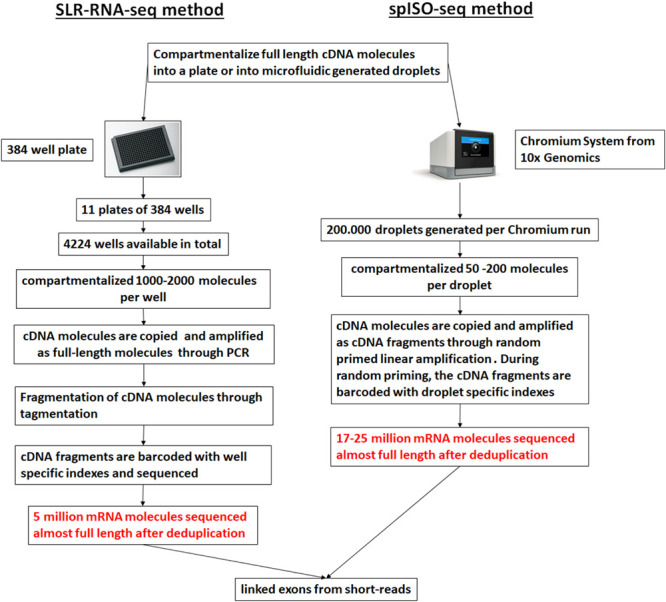
Linked short sequencing reads from cDNA molecules. A ‘384-well plate’-based approach (SLR-RNA-seq; Tilgner et al., 2015) or a droplet-based approach was used to compartmentalize the cDNA molecules and create linked reads. For the droplet-based approach the 10x Genomics platform was used (spISO-seq; Tilgner et al., 2018). The picture was adapted from Tilgner et al. (2018).

Droplet-based microfluidic approaches can be applied to generate sequence information from individual full-length cDNA molecules through the development of the spISO-seq method (Tilgner et al., 2018), which is an improved method over the ‘384-well plate’ based SLR-RNA-seq one (Tilgner et al., 2015). Both these methods are based on the concept of genomic linked-reads (Zheng et al., 2016). In the spISO-seq method, the microfluidic-generated droplets are populated with a small number of full-length cDNA molecules. Inside each droplet the full-length cDNA molecules usually correspond to different genes and are copied, through linear amplification with degenerate primers, in short cDNA fragments, each one tagged with a droplet-specific barcode (Figure 5). A short-read sequencing platform is used to sequence the short cDNA fragments, and cDNA fragments with the same barcode aligning on the same annotated gene model are assumed to correspond to the same transcript. cDNA fragments derived from different exons and found on the same transcript are characteristic of the co-occurrence of the different exons on the same transcript (linked exons), an outcome of the method that the authors call exon coordination (Tilgner et al., 2015). The rational of developing these methods is that the sequencing depth of the short-read sequencing platforms surpasses the long-read sequencing ones and thus more individual molecules can be profiled, necessary for isoform quantification and splicing coordination. Thus, with a high number of assessed molecules these approaches can have advantages over lower throughput single molecule-based approaches. However, with sparser sequencing of all the molecules, a full-length sequence of a single molecule cannot be achieved; rather, mapping is performed using unassembled short reads, which are usually problematic for pseudogenes and repetitive regions (Tilgner et al., 2018).

Despite the limitations, the advantage of these methods (Figure 5) is the low input requirements for spISO-seq (and SLR-RNA-seq), in the order of 100 pg – 1 ng of cDNA, thus making prior amplification unnecessary (and therefore limiting any bias) in most cases. In its application, spISO-seq revealed new molecules in transcript classes such as lincRNAs and antisense trasncripts, however in the case of pseudogenes, mapping was still error-prone compared to long SLRs (Tilgner et al., 2015) or PacBio reads (Sharon et al., 2013). A similar approach to the SLR-RNA-seq has been commercialized by Loop Genomics (United States).

## Future Directions

The last five years have seen a dramatic improvement in the nanopore technology. Both the accuracy and the yield of the ONT MinION platform have significantly increased. In parallel alternative versions of the nanopore platform have been created (GridION, PromethION) that promise a higher parallelization of sequencing, which can offer higher yield. The PacBio platform is also preparing major improvements like the introduction of 8 million ZMW well SMRT cells. Given the increased yields there is a considerable cost reduction in the experimental design that can be accompanied with sample barcoding possibilities. One important advantage of the MinION is that the signal processing and the basecalling can be done in a laptop without the requirement of a server cluster, a feature that has proven its utility in field applications. Overall, the long-read sequencing platforms have been used for a variety of applications that mainly complemented short read approaches, but now, single molecule, long-read sequencing approaches will become a mainstream approach in RNA-Seq.

## Author Contributions

SO, AB, SF, and JR wrote the manuscript. SO, AB, HD, and PB prepared the figures. All authors discussed the final manuscript.

## Conflict of Interest

JR is a member of the MinION Access Program (MAP) and has received free-of-charge flow cells and sequencing kits from Oxford Nanopore Technologies for other projects. JR has had no other financial support from ONT. AB has received re-imbursement for travel costs associated with attending Nanopore Community meeting 2018, a meeting organized by Oxford Nanopore Technologies.

The remaining authors declare that the research was conducted in the absence of any commercial or financial relationships that could be construed as a potential conflict of interest.
