# Supplementary material for: Methodologies for Transcript Profiling Using Long-Read Technologies
Source: Front Genet. 2020 Jul 7;11:606. doi: 10.3389/fgene.2020.00606 (PMC7358353; doi:10.3389/fgene.2020.00606)
Supplement: Supplementary file 1 [file Data_Sheet_1.pdf]

Transcript profiling using long-read sequencing technologies

(Supplementary text and supplementary figures)

Contents

1. Applications of long read sequencing technologies 1

1.1. Identification of gene fusions, integration with the underlying genomic rearrangements and molecular monitoring of disease progression with long read sequencing 1

1.2. Long read sequencing unveils mechanistic aspects of mRNA splicing 2

1.3. Long read sequencing unveils mechanistic aspects of mRNA degradation 3

1.4. Allele specific sequencing 4

1.5. Transcriptome annotation in the presence or absence of a reference genome 4

1.6. Accurate determination of Transcription Start Sites and Transcription End Sites 5

1.7. Error correcting long read sequencing data for genome annotation 5

1.8. Complementing short read data gene model assemblies with long read data 6

1.9. Long read sequencing of highly similar genes 6

1.10. Correcting misannotation with long-read sequencing data 7

1.11. Characterization of long non-coding RNAs and anti-sense transcripts with long-read sequencing 7

1.12. Gene expression quantification and differential expression using long-read sequencing data 8

1.13. Targeted long read sequencing 9

1.14. Characterization of the transcriptome of RNA and DNA viruses 10

1.15. Characterizing the genome of RNA viruses 12

Supplementary tables 13

Supplementary Table 1. Technical comparison between the PacBio Sequel and the ONT MinION platforms. 16

Supplementary Table 2. Examples of organisms whose transcriptome or genome was sequenced on a long-read sequencing platform 22

Supplementary figures 18

Supplementary Figure 1 (previous page). Sequence characteristics of RNA molecules or RNA fragments and their full-length cDNA synthesis methods. 20

Supplementary Figure 2. MagBead loading on the ZMW flow cell. 21

Supplementary Figure 3. Consensus accuracy as a function of long read sequencing coverage. 22

Supplementary Figure 4. PacBio Sequel SMRT sequencing run statistics for the 1M SMRT cell from fragmented genomic DNA samples. 23

Supplementary Figure 5. Sequencing run statistics for the ONT MinION from amplified cDNA and direct RNA experiments........ 24

Supplementary Figure 6. Biological problems addressed with the long-read sequencing platforms relevant to RNA sequencing. 25

Supplementary Figure 7 (previous page). Identification of processing patterns with long-read sequencing........ 28

Supplementary Figure 8 (previous page). Identification of degradation patterns with long-read sequencing........ 30

References 31

1. Applications of long read sequencing technologies

1.1. Identification of gene fusions, integration with the underlying genomic rearrangements and molecular monitoring of disease progression with long read sequencing

Gene fusions are one of the main class of somatic events in cancer genomes that occur when structural chromosomal rearrangements (translocations, inversions, interstitial deletions) brings two separate genes together into a new functioning gene. The first gene fusion (*BCR-ABL1*) was discovered >50 years ago in chronic myeloid leukemia (Nowell and Hungerford, 1961). Since then and with the advancement of new technologies, several others have been discovered in different type of cancers, including carcinomas of the thyroid, salivary gland, prostate, lung, breast, head and neck, brain, skin, gastrointestinal tract, and kidney (Kumar-Sinha et al., 2015). During these years, the technologies for detection of gene fusions have evolved to include DNA (DNA-Seq) and RNA (RNA-Seq) approaches (Parker and Zhang, 2013). The latter has been widely used for transcriptome profiling and detection of expressed chimeric transcripts (likely driver events) from non-expressed passenger events in diverse solid tumors (Maher et al., 2009). A recent systematic analysis of TCGA RNA-Seq data for detection of gene fusions on 9624 tumors across 33 cancer types revealed a total of 25664 fusions with a 63% validation rate (Gao et al., 2018a). Moreover, Lee et al (Lee et al., 2017) developed a database of fusion genes that contains 33316 gene pairs by compiling data from well-known public resources (e.g. COSMIC, OMIM, etc.), published papers using text mining approach and systematic computational analysis of RNA-Seq data such as TCGA. Although these studies give us a rough estimation on gene fusion frequency in tumors, most of them are computationally predicted from short-read RNA-Seq data and remain to be confirmed by more sensitive and accurate experimental or sequencing approaches.

Recently, Nattestad et al. combined genomic structural variant discovery from long-read PacBio genomic sequencing with Iso-Seq PacBio full-length cDNA transcriptome sequencing (Nattestad et al., 2018). As the length of the transcripts expressed from the fused genes is unknown, the Iso-Seq approach was used to have a comprehensive picture of cDNA molecules of different sizes. The authors observed both the genomic rearrangements as well as the fused genes that are produced from the closed proximity of the genes in the rearranged genomic fragments (Nattestad et al., 2018). To be confident about the fused regions only the full-length High-Quality Quivered consensus sequences were used supported by at least 5 full length Iso-Seq reads (Nattestad et al., 2018). The authors discovered three gene fusions at the cDNA level and their corresponding genomic rearrangements that can take place in two or three steps (Nattestad et al., 2018). Gene fusions in other highly rearranged cancer genomes could reveal other instances of a complex type of variation. In general, it is very advantageous to combine long read genomic sequencing with long read RNA sequencing as both approaches validate each other (Nattestad et al., 2018).

RNA-seq has been used for molecular monitoring of patients during disease progression. For instance, the *BCR-ABL* fused gene is a known tyrosine kinase responsible for the chronic myeloid leukemia progression. Tyrosine kinase inhibitors (TKI) halt the progression of the disease until resistant clones appear whose blasts will take over the bone marrow. Early detection of resistant clones is important, as bone marrow depletion or a different pharmacological scheme can be used. Preferably, mutations in both the regulatory and kinase domains as well as co-existing mutations should be detected as early as possible, prior to an expansion of resistant clones. In addition to

point mutations, the BCR- ABL1 protein can be affected by alterations in splicing where whole exons, or smaller parts of exons, are included or skipped from the main transcript. Full length transcripts have been used to characterize biologically important compound mutations (Cavelier et al., 2015), along with splice isoforms. The ability to differentiate between independent and compound mutations is a major advantage of this technology. For example, compound mutations have been associated with moderate and high-level resistance to chemotherapeutic drugs ponatinib and rebastinib, respectively (Zabriskie et al., 2014), while the individual mutants are instead sensitive to these substances.

In the example mentioned above, a 10,000X coverage of *BCR-ABL1* for each of the presented clinical samples was achieved, leading to increased sensitivity and ability to detect resistant clones months earlier than after applying Sanger sequencing (Cavelier et al., 2015). With the ability to sequence the whole transcript, the method permits us to exclude other *BCR-ABL1* mutations as responsible factors for the observed TKI resistance. Furthermore, rare mutations within the regulatory domain of ABL1 have also been reported to lead to TKI resistance in patients without kinase domain mutations (Sherbenou et al., 2010). These rare mutations can be detected and associated with the outcome of the disease.

Gene fusion detection has also been performed on the nanopore platform (Jeck et al., 2019). The authors showed that once the high-quality nanopore reads are used, the platform can detect quickly with high sensitivity and specificity gene fusion events derived from targeted amplification of specific loci even when the tumor fraction is 5-10% (Jeck et al., 2019). The fused genes *BCR-ABL1*, *PAX5-AUTS2*, *KMT2A-MLLT3*, *HAS2-PLAG1*, *EWSR1-FLI1*, *NCALD-PLAG1*, *ETV6-NTRK3*, *SLC34A2 - ROS1*, *RARA–PML*, *EWSR1–FLI1*, *CBFB–MYH11* present in the acute promyelocytic leukemia clinical samples were detected on the ONT platform (Jeck et al., 2019). The fused genes *EML4-ALK*, *KIF5B-RET* were also identified from cDNA amplicons produced from clinical samples of lung adenocarcinoma patients (Suzuki et al., 2017). Similar to the mentioned PacBio studies, the authors phased compound mutations in the EGFR gene (Suzuki et al., 2017). They also phased Single Nucleotide Polymorphisms (SNPs) with fused transcripts for example in the case of the *EFHD1-UBR3* fusion (Suzuki et al., 2017).

1.2. Long read sequencing unveils mechanistic aspects of mRNA splicing

Long read sequencing can be used to assess mRNA processing events. Mechanisms of mRNA splicing can easily be explored. In case of the mRNA splicing, comparison of the unspliced nascent mRNAs versus spliced ones can be used to derive in vivo splicing kinetics (Oesterreich et al., 2016) . For example, although it is known that transcription and splicing can occur simultaneously and influence one another, knowledge of the rate of splicing in vivo was missing. A technique was initially developed that permitted the cDNA synthesis of unprocessed mRNA molecules based on their properties as presented in Supplementary Figure 1. The technique was named long-read SMIT-seq (Supplementary Figure 7). The authors used PacBio to examine both unspliced and spliced reads of the same gene and figured out that splicing starts when Pol II is 26 nt downstream of a 3'SS and splicing ends when Pol II is 129 nt downstream of the 3'SS and on average splicing is complete within 1.4 seconds after 3' SS synthesis, at least an order of magnitude faster than previous estimates (Oesterreich et al., 2016). This indicates that Pol II and the spliceosome are physically closer during splicing catalysis than previously anticipated and splicing catalysis starts as soon as the 3'SS exits Pol II. The authors concluded that the mechanistic implications of these findings shed new light on the physical and temporal relationship between transcription and

splicing catalysis, the relative speed with which the spliceosome assembles, and how opportunities for regulation arise in vivo. In another study the authors used the same technique to assess directly the order of intron removal as can be examined in nascent RNAs from multi-intron transcripts in the fission yeast *Schizosaccharomyces pombe* relative to the progression of transcription which is marked by nascent RNA 3′ ends (Herzel et al., 2018). The authors observed fully spliced, fully unspliced, and partially spliced nascent RNAs, noticing that ordered intron removal is not strictly enforced (Herzel et al., 2018). The high proportion of multi-intron transcripts that were either fully spliced or fully unspliced, suggested that splicing of any given intron is dependent on the splicing status of other introns in the transcript (Herzel et al., 2018).

The ability to sequence both processed and unprocessed full-length transcripts can give clues to the biogenesis of the processed transcripts. For example, in the case of insect mitochondrial gene transcription it was possible to propose a biogenesis model of mature mRNA and tRNA molecules from polycistronic RNAs. In the single-celled alga *Euglena gracilis*, the authors sequenced both processed and unprocessed transcripts in order to identify the order of removal of conventional and nonconventional introns for a subset of genes that have both types (Guminska et al., 2018). Based on the abundance of the different intermediates between the unprocessed and the fully processed transcripts, the authors concluded that the conventional introns are removed before the nonconventional introns, that the nonconventional intron removal is quicker than the conventional intron one and that the conventional intron removal does not strictly proceeds collinearly with the progress of transcription but all possible combinations of occurrence of conventional introns can be noticed in the sequenced transcripts (Guminska et al., 2018). Additionally, they noticed that there is a lag in the processing time between conventional and non-conventional intron removal indicating that the conventional intron removal might take place co-transcriptionally whereas the non-conventional intron removal might take place post-transcriptionally (Guminska et al., 2018).

1.3. Long read sequencing unveils mechanistic aspects of mRNA degradation

Canonical mRNA decay involves deadenylation followed by 3′-to-5′ exonucleolysis by the exosome and decapping followed by 5′-to-3′ exonucleolysis by XRN1 (Schoenberg and Maquat, 2012). The 5' degradation pattern is conceptually appealing because the mRNA that undergoes 5′ -to-3′ decay will not be able to reinitiate translation while the last protein molecule that it produces will be full length. Much less clear, however, is what happens to translated mRNAs when degradation occurs in the 3′ -to-5′ direction. The authors (Ibrahim et al., 2018) investigated human mRNA dynamics in vivo by developing the Akron-SMRT method (Supplementary Figure 8) after exploiting the properties of the degradation fragments as presented in Supplementary Figure 1. In this method, capped RNAs are enriched through digestion with the Terminator 5′ phosphate (P)-dependent exonuclease (Supplementary Figure 1). The authors found that the capped RNAs, are co-translationally degraded from the 3′ end because these fragments were found in translating ribosomes. This indicates that canonical human mRNAs undergo repeated co-translational and ribosome-phased endonucleolytic cuts at the exit site of the mRNA ribosome channel, in a process called "ribothrypsis" (Ibrahim et al., 2018). We note here that the authors based their conclusions on a modification of the Akron-SMRT method, the Akron3-seq method which is similar to the Akron-SMRT but permits the sequencing of libraries on a short-read sequencing platform. The authors argued that due to the high sequencing depth of the short-read sequencing platforms, the Akron3-seq method permitted them a whole-transcriptome analysis, in contrast to the Akron-SMRT, which interrogated a few targeted genes.

1.4. Allele specific sequencing

The unambiguous mapping of long reads permit the identification of allele specific expression (Sun et al., 2018). The authors investigated the effects of cis-regulatory divergence on RNA decay in an F1 hybrid mouse and quantified allele-specific differences in RNA decay rates. They identified 621 genes exhibiting significant cis-divergence, contributed by genetic variants affecting microRNA binding and RNA secondary structures, which are features that affect RNA splicing, RNA polyadenylation, RNA stability and RNA degradation (Bevilacqua et al., 2016;Duchaine and Fabian, 2019). The authors used PacBio to validate the observations based on the Illumina data. Using the PacBio RS system, they deep-sequenced the RT–PCR products using primers targeted at the regions with no sequence variants between the two alleles. The longer read length allowed the assignment of the PacBio reads to the parental alleles by utilizing CCS reads without any ambiguity. Allelic ratios of the read counts were then compared between the two time points. The allelic decay rates were closely correlated with those determined using the Illumina approach.

In the case of the ONT platform and the direct RNA sequencing platform, allele specific expression detection has been explored from raw read data (Workman et al., 2018b). Due to the high error rate of the reads, allele specific analysis was only possible in well characterized genomes like the GM12878. Raw reads that contain at least two variants known to be heterozygous in GM12878 were annotated as maternal, paternal or unassigned. To reduce the chances of a false positive from nanopore sequencing errors as well as to avoid positions with systematic errors, the authors examined only genes where, greater than 75% of the reads that contain at least two variants, agreed on the parental allele of origin. The authors found 492 genes that showed allele specific gene expression as well as 34 genes with allele specific isoform expression out of 2,917 genes with at least 10 haplotype informative reads. These numbers are likely an underestimation of the true number of allele specific transcripts, as only deeper sequencing will allow the analysis of low abundance transcripts.

Although not directly relevant to allele specific sequencing but given that identification of SNPs is important before phasing, the long-read sequencing platforms have been used to identify SNPs corresponding to RNA editing events. By comparing mitochondrial genomic DNA assemblies and the corresponding PacBio generated mitochondrial transcripts, RNA editing was observed in select species of *Leucaena* (Kovar et al., 2018).

1.5. Transcriptome annotation in the presence or absence of a reference genome

The long-read sequencing technologies have been extensively used in characterizing genes and isoforms across multiple organisms. As no assembly is required, but rather multiple rounds of error correction, the gene and isoform models are reliable enough and can be used without the necessity the corresponding genomes to be either well characterized or even present. Novel genes, novel isoforms, corrected gene and isoform models, novel alternative splicing sites and transcription start sites as well as alternative polyadenylation sites are reported in all these studies. The long-read RNA-seq methodologies have been used across different organisms and some examples are presented in Supplementary Table 2. Databases have been generated, hosting Iso-Seq data along

with an in-depth analysis of the datasets and visualization of the full-length transcript isoforms (Xie et al., 2018).

Studies that use the PacBio Iso-Seq approach to characterize de novo transcripts are usually correcting and expanding on a combination of Illumina short-read RNA-seq assemblies and predicted gene models. Novel genes are frequently reported. For example, in *Oryctolagus cuniculus* long read RNA-seq, 23% of genic loci have not been annotated before (Chen et al., 2017). Similarly, in the *Sorghum bicolor* transcriptome over 2,100 novel genes are reported (Abdel-Ghany et al., 2016).

The main advantage of the long-read platforms is the accurate characterization of isoforms without employing any statistical prediction approaches. In the short-read sequencing platforms although most transcript reconstruction algorithms achieve good performance at assigning the corresponding exons to the individual genes, accuracy is a lot lower for assembly of complete transcripts (Steijger et al., 2013). Indeed, in *Oryctolagus cuniculus* long read RNA-seq, 66% of the observed isoforms were not annotated before (Chen et al., 2017) whereas in the *Sorghum bicolor* transcriptome the corresponding number is 40.7% (Abdel-Ghany et al., 2016).

1.6. Accurate determination of Transcription Start Sites and Transcription End Sites

An advantage of the long-read platforms is the accurate determination of alternative Transcription Start Sites (TSS) or alternative Termination End Sites (TES). In the standard short-read Illumina RNA-seq the coverage at the end of the individual molecules drops. The result of this is that the TSS and the TES of low abundant transcripts is not demarcated properly. The exact position of the TES is more problematic than the TSS as due to the paired end nature of sequencing of the cDNA fragments, there is no guarantee that all the read up to the polyA tail will be sequenced. Nevertheless, there are techniques that can target specifically only the 5’ end (NanoCAGE (Salimullah et al., 2011)) or the 3’ end (PAS-seq (Shepard et al., 2011)) of the cDNA molecules. In the *Oryctolagus cuniculus* long read RNA-seq, 11,184 alternative polyadenylation sites were detected in 3,492 genes (Chen et al., 2017) whereas in the *Sorghum bicolor* long read RNA-seq 11,013 polyadenylation sites were detected in 14,550 genes (Abdel-Ghany et al., 2016). In moso bamboo long read RNA-seq 25,069 polyadenylation sites were detected in 11,450 genes and 6,311 of these genes had more than one polyadenylation site (Wang et al., 2017).

In another study the authors identified the 5′ and 3′ ends of bacterial operons by specifically sequencing full length unprocessed polycistronic transcripts through a methodology called SMRT-Cappable-seq that enriches full-length primary transcripts with triphosphorylated 5’ ends and hydroxylated 3’ ends (Yan et al., 2018) (Supplementary Figure 1). For 1/3 of the detected operons the authors revised the genes involved by adding at least one more gene (Yan et al., 2018).

1.7. Error correcting long read sequencing data for genome annotation

Ideally, characterizing the transcriptome with a long-read sequencing platform is the optimal approach. Data from short-read sequencing constitute often part of the analysis pipeline, as long-read sequencing does not reach enough depth for accurate consensus based basecalling of low abundance transcripts. In this case the long reads are error corrected with the short reads. For example, in *Oryctolagus cuniculus long* read RNA-seq, 86.2% of the constructed isoform

sequences contained erroneous fragments that were corrected with the short read sequences (Chen et al., 2017).

Error correction of long reads can be performed with either short read sequencing reads like the FMLRC method (Wang et al., 2018), the HySeMaFi method (Ning et al., 2017) or with a reference genome like in the case of TranscriptClean (Wyman and Mortazavi, 2019). Short- and long-read cDNA sequencing data can be combined together with protein evidence, and ab initio prediction algorithms to generate accurate genome annotations as has been implement in the LoReAn package (Cook et al., 2019).

Additionally, feature classification can be used to increase the confidence in the identified isoforms. In this case, in order to assert that the exon structure is the correct one, machine learning approaches can be used that can classify the non-highly confident isoforms and discriminate artifacts from true novel transcripts based on a set of features. For example, in the SQANTI software (Tardaguila et al., 2018) a Random Forest classifier employs different features to classify the transcripts into highly confident ones, based on both short read and long read sequencing data. In another case the PLEX (Li et al., 2014) software has been used to classify transcripts generated with the error prone long read technologies into potential long non-coding RNAs or messenger RNAs in the absence of genomic sequences or annotations. In this case a support vector machine algorithm has been trained on calibrated k-mer frequencies derived from known long non-coding RNAs and messenger RNAs (Li et al., 2014). Additionally, the CPAT tool (Wang et al., 2013) uses a logistic regression model, trained with four difference sequence features, to classify coding and noncoding transcripts.

1.8. Complementing short read data gene model assemblies with long read data

Gene models assembled from short-read data can be validated with transcripts sequenced on long-read sequencing platforms. In one study the authors identified, in the marine syllid polychaete *Odontosyllis undecimdonta*, four transcripts from Illumina assembled transcript models as well as from ONT MinION cDNA transcripts, that matched Mass Spectrometry data corresponding to potential luciferase coding genes (Schultz et al., 2018). Through cloning, protein expression, protein purification and luciferin assays they validated that only two of them were coding for functional luciferase proteins (Schultz et al., 2018). In another study, long read cDNA sequencing was used to validate protein isoforms of industrially relevant hydrolases that were identified through haplotype reconstruction in the samples of a metatranscriptome from a rumen microbial community (Nicholls et al., 2017).

1.9. Long read sequencing of highly similar genes

Short-read RNA-seq of paralogous genes makes both their de-novo assembly and their unique genomic assignment, problematic. On the contrary, long read RNA-seq of paralogous genes permits their assignment in the correct genomic positions. For example, in *Oryctolagus cuniculus* the gene structure of nine paralogous genes of the Major Histocompatibility Complex (MHC) were well recovered by PacBio transcripts in comparison with reference annotation (Chen et al., 2017). Assembled transcripts from short reads by genome-guided methods (Cufflinks (Trapnell et al., 2010)) performed adequately in eight of these MHC paralogous genes to identify the gene structure

but some of the observed PacBio isoforms were missing (Chen et al., 2017). On the contrary, the assembled transcripts of the MHC locus using de novo approach were poorly assembled showing fragmented and confusing gene models (Chen et al., 2017). The PacBio CCS reads have been used to characterize novel alleles from full-length MHC class I cDNA sequences expressed by a cohort of cynomolgus macaques (Westbrook et al., 2015;Karl et al., 2017). A similar work with PacBio CCS cDNA reads was done to characterize novel KIR alleles expressed by a cohort of 30 cynomolgus macaques (Prall et al., 2017).

In another study, the authors used nanopore cDNA sequencing to find the expression levels of the different alleles expressed from the paralogous loci of the *NOTCH2NL* gene (Fiddes et al., 2018) using a targeted cDNA capturing approach. The *NOTCH2NL* gene is expressed in radial glial and is responsible for delaying differentiation of neuronal progenitors into cortical neurons (Fiddes et al., 2018). Each of these alleles can produce distinct protein or protein-abundance variants with a different functional effect on the activation of the NOTCH2 receptor (Fiddes et al., 2018). Similarly, PacBio was also used to assess the expression levels of paralogous genes (>99% identity between them) from 19 gene families that are present in human-specific segmental duplications and that are having a brain specific gene expression pattern (Dougherty et al., 2018). The IsoCon (Sahlin et al., 2018) software was developed to assign separate transcripts into putative gene copies and to derive copy-specific exon sequences and splice variants from multigene families where the individual gene copies can have in some cases a high sequence identity (up to 99.99%). In this situation teasing out sequencing errors from true variants can be difficult and the use of a reference genome for correction might not be effective as the variability of gene copies might not be reliably captured by the reference (Sahlin et al., 2018).

1.10. Correcting misannotation with long-read sequencing data

The long-reads can also overlap exons from adjacent annotated genes. This can either reflect misannotations or represent read-through transcripts. For example, 3.4% of the unique transcripts identified in the *Sorghum bicolor* long-read RNA-seq overlap two adjacent annotated genes (Abdel-Ghany et al., 2016). We can assume that the misannotations can derive from exonic regions of low abundant genes which are partially covered from short-reads or from long genes whose partial assembly from short-read data failed to produce the full-length transcript. These gene fragments can be directly connected with just a few long-read sequenced molecules because there is no stochastic sampling of the cDNA fragments as the cDNA molecules are full length. In the case of the HSV-1 (Tombacz et al., 2017;Depledge et al., 2018), the long-read RNA-seq revealed a greater complexity of the viral transcriptome as the authors observed spliced HSV-1 read-through transcripts that encode a cryptic class of novel protein fusions which provide evidence for disruption of transcription termination for a number of viral transcription units. In another study, the authors used Iso-Seq to improve the gene annotation of *Anopheles stephensi* and identified 6 trans-splicing events (Jiang et al., 2017).

1.11. Characterization of long non-coding RNAs and anti-sense transcripts with long-read sequencing

LncRNA gene annotations remain incomplete and poorly characterized (Derrien et al., 2012) due to their low steady state levels (Derrien et al., 2012) which results in partial gene structures and

lack of terminal exons or splice junctions between adjacent exons, as the sequencing coverage is usually low (Steijger et al., 2013). Accurate gene annotation requires the use of high-confidence transcriptomic evidence, such as sequencing of full-length cDNA or large cDNA fragments derived from 5' and 3' RACE (Lagarde et al., 2016). SMRT sequencing has been used in combination with targeted RNA capture (RNA Capture Long Seq) (Lagarde et al., 2017). This method captures full length unfragmented cDNAs and enriches the low abundant full-length transcripts of long-noncoding RNAs. In another study, novel transcripts and long non-coding RNAs were also observed in PacBio data of human mitochondria (Gao et al., 2018b).

Long genes are more difficult to reconstruct using transcriptome assembly by short-read technologies. In the maize long-read RNA-seq, 867 novel high-confidence lncRNAs were identified that had a much longer mean length than those identified by Illumina short-read sequencing assembly (Wang et al., 2016). Additionally, as the transcripts are sequenced full length, the long-read platforms permit the unambiguous assignment of noncoding status. For example in the Red Clover transcriptome characterization, the authors identified 4,333 lncRNAs compared with 11 lncRNAs present on the reference genome (Chao et al., 2018b).

In another study the authors used PacBio SMRT sequencing to identify the exon structure of natural antisense transcripts in the moso bamboo seedlings in the presence or absence of exogenous gibberellins treatment (Zhang et al., 2018). These transcripts were further validated with strand specific short-read RNA-seq (Zhang et al., 2018).

1.12. Gene expression quantification and differential expression using long-read sequencing data

Short-read RNA-seq can be used not only to polish the long-read RNA-seq but also to complement it. For example, the short-read data can be used to quantify differentially expressed exons and genes between the different conditions in a given sample. Afterwards, the long-reads can be used to identify the precise differential expressed isoform models for the genes that showed the differential splicing or the differential expression pattern. This approach was followed in order to find differentially expressed isoforms in Arabidopsis thaliana in response to 6h and 48h of abscisic acid (ABA) treatment (Zhu et al., 2017).

A few studies have performed differential gene expression analysis with the long-read sequencing data. In one study the authors performed differential gene expression analysis based on ONT direct RNA sequencing data comparing the two metabolic stages of diauxic growth of *Saccharomyces cerevisiae*, namely the respiro-fermentative growth on glucose and the oxidative growth on ethanol (Jenjaroenpun et al., 2018). Bayega et al. (Bayega et al., 2018) performed differential gene expression analysis based on cDNA sequencing, between the 1 hour interval stages of the first 6 hours of the Olive fruit fly (*Bactrocera oleae*) embryonic development. Differential expression from only the long-read data has also been performed for example to identify differentially expressed genes in response to drought treatment in *Sorghum bicolor* (Abdel-Ghany et al., 2016).

In another study the authors sequenced full length cDNA from Chronic Lymphocytic Leukemia samples with and without mutations of the splicing factor SF3B1 (Tang et al., 2018). As the mutated splicing factor is responsible for aberrant splicing patterns, the authors identified that the mutant splicing factor $SF3B1^{K700E}$ induces alternative upstream 3' splice sites on certain transcripts as well as a reduction of isoforms that showed intron retention (Tang et al., 2018).

Iso-Seq has been used to characterize the different isoforms in comparative transcriptome analyses of human and rhesus macaque cerebellum which led to the identification of lineage specific isoforms in humans (Zhang et al., 2017). PacBio was also used to quantify the abundance of L1 retrotransposon elements, in comparison to the abundance of L1-related sequences that are co-transcribed within genes, through a 5' RACE assay (Deininger et al., 2017).

Finally, the PRAPI pipeline can be used to characterize alternative transcription initiation, alternative splicing, alternative cleavage and polyadenylation from long-read sequencing data or from a combination of long-read and short-read sequencing data (Gao et al., 2018c). It can also be used to annotate antisense transcripts, novel genes, to correct mis-annotated ones as well as to perform differential expression analysis of all the previous categories across different samples (Gao et al., 2018c).

1.13. Targeted long read sequencing

The least expensive way of performing full length cDNA sequencing is through targeted full-length amplification of the cDNA locus of interest using long amplification PCR. For example, on the PacBio platform, the human *FMR1* (Pretto et al., 2015) and the Neurexin-1-alpha (Schreiner et al., 2014;Treutlein et al., 2014) cDNAs have been specifically targeted for SMRT sequencing. In another study, cDNA amplicons of gluten genes were individually barcoded, pooled and sequenced with SMRT sequencing (Zhang et al., 2014). This permitted the characterization and phylogenetic reconstruction of gluten gene families across 10 wheat cultivars, even without any draft genome sequence available (Zhang et al., 2014).

Several eukaryotic genes can encode hundreds to thousands of isoforms. This can be accomplished through a combination of constitutive exons and exon clusters. The exons within each cluster are spliced in a mutually exclusive manner leading to a large number of alternatively spliced isoforms (Yang et al., 2011). Assembling these isoforms from short read data is usually unsuccessful as the variable exon clusters are located far from one another on the mRNA sequence and the exons within each cluster can be up to 80% identical to one another at the nucleotide level (Bolisetty et al., 2015). In Drosophila (Brown et al., 2014), example of genes with complex isoforms are the *Dscam1*, *MRP*, *Mhc*, and *Rdl* genes. For these Drosophila genes, the ONT MinION platform has been used to characterize the type and abundance of the complex isoforms (Bolisetty et al., 2015). In human, examples of genes with complex isoforms are the Voltage Gated Calcium Channels (VGCCs). Alternative spliced isoforms encode functionally different VGCCs (Raj and Blencowe, 2015) and therefore only full length cDNA sequencing can identify the entire protein coding sequence of these isoforms. One of these genes is the *CACNA1C* gene, that consists of at least 50 known exons. The ONT MinION was used to sequence long cDNA amplicons and the authors identified 90 isoforms from which 83 were novel (Clark et al., 2018).

SMRT sequencing has been used to sequence a *FANCB* aberrant transcript containing exon 3 duplication which is predicted to introduce a stop codon in the FANCB protein and was identified in a Fanconi anemia patient with a mild phenotype (Asur et al., 2018).

A similar cDNA amplicon study with the ONT MinION was performed with the human *PKD1* gene (Lea et al., 2018), a gene responsible for the major form of autosomal dominant polycystic kidney disease. For this gene, the authors observed that the gene is differentially spliced over introns 21 and 22 and by applying long read sequencing were able to study the different isoforms resulting from combinations of exons 20-24 (Lea et al., 2018). The same platform was used to

sequence cDNA amplicons from the *ABCA7* gene whose Premature Termination Codon (PTC) mutations have been identified as an intermediate-to-high penetrant risk factor for late-onset Alzheimer's disease (De Roeck et al., 2017). These PTC mutations can frequently lead to the nonsense mediated decay of the isoforms that carry the exon with the corresponding mutation (Hillman et al., 2004). The authors screened early onset Alzheimer disease patients for mutations on the *ABCA7* gene in order to identify the exons that carry novel PTC mutations and identified isoforms that exclude the exon with the PTC mutation and thus rescue the transcript from nonsense mediated decay (De Roeck et al., 2017). They also measured the varied expression levels of the transcripts that carry exons with the PTC mutation and identified the sequence of truncated proteins (De Roeck et al., 2017).

In another study the authors used ONT MinION to sequence long range PCR generated cDNA amplicons (~6kb) of the *BRCA1* transcripts in order to resolve the exon structure of the whole transcript which enabled them to predict in-frame and out-of-frame coding events (de Jong et al., 2017). This is important for interpreting the clinical significance of spliceogenic variants and to identify mRNA splicing changes that are expected to disrupt protein function either through truncation or in-frame deletion of important regions of the encoded proteins (de Jong et al., 2017).

1.14. Characterization of the transcriptome of RNA and DNA viruses

In general the viral transcriptomes are usually highly complex showing embedded RNAs, complex transcripts (Prazsak et al., 2018), bicistronic and polycistronic transcripts, transcript isoforms as well as an extended meshwork of overlaps between the transcripts (Tombacz et al., 2016;Moldovan et al., 2017b;Prazsak et al., 2018). Compared to the short-read sequencing platforms, the long-read sequencing ones are the ideal technology to characterize in greater detail and accuracy all this complexity which can provide useful information to target transcript candidates for controlling the viral replication and propagation.

Indeed, all the available SMRT and nanopore library preparation methods have been used to characterize the viral RNA transcriptomes. For example, the non-amplified and amplified SMRT methods, the PacBio Iso-Seq protocol, the Nanopore full-length cDNA-sequencing, the Nanopore direct RNA-sequencing, as well as the Nanopore cDNA-sequencing on 5′ Cap-selected samples for both oligo (d)T and random primed reverse transcribed samples have been used either exclusively or in combination (Tombacz et al., 2018b) to characterize the viral transcripts. Transcriptomes from a large number of viruses have been extensively characterized and some examples are presented in Supplementary Table 2.

The transcriptome of the viruses has been characterized through either targeted cDNA amplification of the viral transcripts or through RNA-seq or direct RNA-seq of the infected viral host transcripts for either the lytic or the latent phase of the viral life cycle. In all the cases except the targeted amplification, the sequenced reads are aligning in both the host genome and the genome of the virus if known. The majority of studies identified novel RNA molecules, alternatively transcribed and processed transcripts, coding and non-coding RNAs as well as they permitted to distinguish between transcript isoforms including splice and length variants. In a lot of cases the studies are uncovering a very complex transcriptome. For example, the Vaccinia virus (VACV) has a unique form of gene regulation where the transcriptional overlaps generated by the read-through mechanisms are very frequent. Additionally, the transcription patterns of VACV genes exhibit an increased stochasticity, which includes a large number of transcriptional start sites (TSSs) and transcription end sites (TESs) even within the open reading frames (ORFs) (Tombacz

et al., 2018b). Therefore, the use of full-length sequencing methods is important to identify the transcript ends with relative base pair precision (Tombacz et al., 2018b). These alternative TESs can potentially influence RNA metabolism by allowing or preventing the binding of microRNAs to the viral RNA (Barth et al., 2008) and by facilitating or preventing deadenylation (Dickson et al., 2012).

Long read RNA-seq has also been used to characterize polycistronic mRNAs in the Herpesvirus HSV-1 (Tombacz et al., 2017;Depledge et al., 2018). These viral polycistronic transcripts are different from the prokaryotic polycistronic transcripts because the polycistronic transcripts overlap with each other and the same viral gene can be expressed from more than one polycistronic transcripts. These overlapping polycistronic architecture can be achieved by a common poly-A signal and varying transcription start sites that are controlled by distinct promoters. The authors (Tombacz et al., 2017;Depledge et al., 2018) report new transcription initiation sites that produce mRNAs encoding novel or alternative ORFs. Transcription initiation sites are critical for productive gene expression as their location relative to the translation initiation site determines the length and composition of the 5′ UTR of mRNAs, which can have profound effects on translation efficiency.

Multi-platform data integration is also important as the same sample can result in different read length distributions with the longest reads derived from direct RNA sequencing followed by the PacBio Sequel and ONT platforms depending on the library preparation methods (Boldogkői et al., 2018b). The method can also be used to infer the different viral clones present in the genome of the host cell. For example, for the Porcine Endogenous Retrovirus (PERV) the authors uncovered several intronic regions in the transcriptome data after mapping them back on the genome (Moldovan et al., 2018a). The authors confirmed that these introns were not alternative spliced products but rather deleted genomic segments indicating the existence of at least four different PERV clones in the genome of the host PK-15 cells (Moldovan et al., 2018a). For the Varicella Zoster Virus (VZV), the authors defined a new class of transcripts the nroRNA that are transcripts encoded by the genomic region located in close vicinity to the viral replication origin which suggests an interference between the replication and transcription machineries (Prazsak et al., 2018). A similar case was observed in Pseudorabies Virus (PRV) after profiling the transcriptome with PacBio (Tombacz et al., 2015). For the VZV virus, the complex meshwork of transcriptional read-throughs can regulate gene expression through a transcriptional interference mechanism (Boldogkoi, 2012;Prazsak et al., 2018). Additionally, the authors detected RNA editing in a novel non-coding RNA molecule from the nanopore sequenced reads (Prazsak et al., 2018). Furthermore, in the VZV virus, additional to the lytic phase transcriptome, the latent phase transcriptome was characterized, and it was showed that the three genes that are expressed during the latent phase can occasionally form a single transcriptional unit (Prazsak et al., 2018). ONT and PacBio-based studies have also detected a number of embedded transcripts with in-frame truncated ORFs in several herpesviruses (Prazsak et al., 2018) coding for potentially truncated polypeptides. In the case of the VZV virus, four complex transcripts, which are multigenic RNAs that contain one or more genes in opposite (Quick et al., 2017) orientations, have been characterized (Prazsak et al., 2018).

The cDNA fragments derived from 5’ rapid amplification of cDNA ends (5’-RACE) from the transcriptome of the Mouse Papillomavirus type 1 have been sequenced with the PacBio Iso-Seq method in order to map the viral transcription start sites (Xue et al., 2017). In another cases, the PacBio Iso-Seq was used to sequence cDNAs derived from total RNA of Porcine Circovirus type 1 infected cells that was reversed transcribed with either oligo (dT) primers or random hexamer primers (Moldovan et al., 2017a).

Lastly for a more general review of the application of long read sequencing technologies on the transcriptomic analysis of complex viral genomes see Depledge et al.(Depledge et al., 2019)

1.15. Characterizing the genome of RNA viruses

Although not a transcriptome, the genome of RNA viruses can be sequenced with the same methods. From relative pure samples, RNA/cDNA hybrids have been sequenced at a reasonable depth, without amplification, on the nanopore platform (Kilianski et al., 2016). For the RNA viruses if the titer is low or enough coverage is required for strain typing and variant identification an enrichment method needs to be followed. In this case either an amplicon or a non-amplicon based approach can be used. The non-amplicon based approaches are using RNA baits on streptavidin beads and are supposed to improve library complexity and uniformity of the sample, thus aiding in the detection of single-nucleotide variants. This method was used for enrichment of the Influenza virus A genome cDNA from the cDNAs of the MDCK cell line (Eckert et al., 2016). In another study, cDNA amplicons from the Zika Virus (ZIKV) were sequenced on the ONT MinION in order to identify the origin and epidemic history of ZIKV in Brazil (Faria et al., 2017). Similarly, the ONT MinION sequencing of multiplexed cDNA amplicons, has been used to measure the diversity of the West Nile virus (Grubaugh et al., 2019) and the defective viral emergence in the Flock House virus (Jaworski and Routh, 2017). MinION has been used to sequence the genome of multiple RNA viruses and some examples are presented in Supplementary Table 2.

Metagenomic sequencing allows for identification of multiple RNA pathogens within a sample in a non-targeted and unbiased approach. With this approach the chikungunya and the dengue viruses have been identified in total RNA extracted from patient samples and permitted the identification of co-infections (Kafetzopoulou et al., 2018) as well as it has the potential to identify novel pathogens. PacBio was used to sequence the genome of the Hepatitis C virus at near full length which permitted the identification of viral quasispecies in clinical samples after phasing the observed variants (Bull et al., 2016).

Supplementary tables

**sequencing company**	PacBio	ONT	ONT
**sequencing platform**	Sequel	MinION	MinION
**system version**	v6.0		
**chemistry version**	v3.0	SQK-PCS109	SQK-RNA001
**flow cell version**	SMRT Cell 1M	R9.4.1	R9.4.1
**type of sequenced material**	cDNA	cDNA	RNA
**sample preparation method**	reverse transcription with or without cDNA amplification followed with sequencing of cDNA molecules	reverse transcription with or without cDNA amplification followed with sequencing of cDNA molecules	reverse transcription to make the first strand of cDNA, in order to melt the secondary structures of RNA, followed with sequencing of the RNA strand
**type of strand sequenced**	for each cDNA molecule the sense AND antisense strand is sequenced	for each cDNA molecule the sense OR antisense strand is sequenced	the RNA strand is sequenced

**starting amount of RNA needed for the sample preparation method**	As a starting material either an enriched polyA+ fraction is used or total RNA with the equivalent amount of polyA+ RNA (1 - 5% of the total RNA is polyA+). If the reverse transcription of the full-length cDNA synthesis protocol is followed with cDNA amplification, then ~1 - 6 ngs polyA+ RNA are used as a starting material or the equivalent 50 - 300 ngs of total RNA. If no cDNA amplification is followed, then ~100 ng polyA+ RNA are needed.		~500ng polyA+ RNA are used to make a cDNA/RNA hybrid
**amount of cDNA or hybrid cDNA/RNA needed for the preparation of the sequencing library**	~ 100 fmoles of cDNA (80 ngs for the Sequel and 160 ngs for the Sequel II platform)	~ 100 fmoles of cDNA (100-200 ngs of cDNA with average length 1.5 kb)	all the above hybrid cDNA/RNA

**type of sequencing method**	multiple sequencing passes of the same molecule (Circular Consensus Sequencing)	for a given cDNA molecule usually there is only 1 sequencing pass of either the sense or antisense strand. Multiple sequencing passes of the same molecule possible with an adapted library preparation method (linear consensus sequencing)	for a given RNA molecule there is only 1 sequencing pass of the RNA strand
**average length of sequenced reads**	~12.5 kb (Multiple of the average length of the sequenced cDNA molecules due to the sequencing method followed)	same as the average length of the cDNA molecules	same as the average length of the RNA molecules
**average yield in Gigabases**	~7.69	~15.5	~2.12
**average number of sequenced reads per flow cell**	~612,000 reads (~60% of the total number of microwells; 1,000,000 microwells for the 1M SMRT flow cell)	~16.2 million reads per flow cell run (the flow cell was run up to the complete degradation of the electrochemistry)	~1.96 million reads per flow cell run (the flow cell was run up to the complete degradation of the electrochemistry)
**basecalling accuracy (for the human reference)**	~85%	~90%	~86%

**possibility for multiplexing multiple samples in the same flow cell**	Yes	Yes	No
**GC bias present**	No	No	No
**length bias present**	Yes. Depending on the sample preparation and flow cell loading procedure followed, the length distribution of the sequenced reads does not match the length distribution of the original solution of the cDNA molecules. For example, if the MagAttract loading procedure is followed then cDNA molecules equal or less than 600 bp are not sequenced. Additionally, if the Iso-Seq sample preparation method is followed then the cDNA is first size fractionated and then the fraction(s) with the enriched sizes of interest are sequenced.	No	No
**sequencing speed**	~3-4 bases/second	~350 bases/second	~70 bases/second

Supplementary Table 1. Technical comparison between the PacBio Sequel and the ONT MinION platforms. The table summarizes the information presented in the main text. The table is based on the performance characteristics of the 1M SMRT flow cell (1,000,000 microwells) from the PacBio Sequel system and of the R9.4.1 MinION flow cell from ONT (~1,600 nanopores). For higher throughput systems, like the PacBio Sequel flow cell with 8,000,000 microwells or the flow cells with ~6,500 nanopores on the ONT PromethION platform, the yield

(number of reads and total Gigabases sequenced) scales linearly with the number of microwells or nanopores respectively. For more information on the individual points presented in the table the reader is referred to the main text.

Sequencing the transcriptome of plants	Sequencing the transcriptome of fungi	Sequencing the transcriptome of animals	Sequencing the transcriptome of viruses	Sequencing the genome of RNA viruses
Red Clover (Trifolium pratense) (Chao et al., 2018b)	The edible mushroom Shiitake (Lentinula edodes) (Park et al., 2017)	Human (Homo Sapiens) (Sharon et al., 2013;Tilgner et al., 2014)	Porcine endogenous retrovirus (Moldovan et al., 2018a)	Venezuelan equine encephalitis virus vaccine (Kilianski et al., 2016)
Sorghum bicolor (Abdel-Ghany et al., 2016)	Cordyceps militaris (Chen et al., 2019)	Snake (Echis coloratus) venom gland (Hargreaves and Mulley, 2015)	Varicella zoster virus (Tombacz et al., 2018a)	Ebola virus (Greninger et al., 2015;Kilianski et al., 2016)
Arabidopsis thaliana (Zhu et al., 2017)		Pig (Sus scrofa) (Liu et al., 2017a)	Autographa californica multiple nucleopolyhedrovirus (Boldogkői et al., 2018a;Moldovan et al., 2018b)	Plum pox virus (Bronzato Badial et al., 2018)

**Moso Bamboo (Phyllostachys edulis)(Wang et al., 2017)**		**Rabbit (Oryctolagus cuniculus) (Chen et al., 2017)**	**Herpes simplex virus type-1 (Boldogkői et al., 2018b;Depledge et al., 2018)**	**Newcastle disease viruses (Butt et al., 2018)**
**Wheat (Triticum aestivum) (Dong et al., 2015)**		**Chicken (Gallus gallus) (Kuo et al., 2017)**	**Bovine Leukemia Virus (Durkin et al., 2016)**	**Yellow fever virus (Faria et al., 2018)**
**Maize (Zea mays) (Wang et al., 2016)**		**Flesh fly (Sarcophaga peregrina) (Kim et al., 2018b)**	**Varicella zoster virus (Prazsak et al., 2018)**	**Venezuelan Equine**
**Alfalfa (Medicago sativa) (Chao et al., 2019;Luo et al., 2019)**		**Parasitic hookworm (Ancylostoma ceylanicum) (Magrini et al., 2018)**	**Human cytomegalovirus (Balazs et al., 2018)**	**Encephalitis Virus (Russell et al., 2018)**
**Sugarcane (Saccharum officinarum) (Hoang et al., 2017;Piriyapongsa et al., 2018)**		**Pond loach (Misgurnus anguillicaudatus) (Yi et al., 2018)**	**Vaccinia virus (Tombacz et al., 2018b)**	**Bovine Herpes Virus (McCabe et al., 2018)**

**Astragalus membranaceus (Li et al., 2017)**		**Cave nectar bat (Eonycteris spelaea) (Wen et al., 2018)**	**Pseudorabies virus (Tombácz et al., 2018)**	**Bovine Parainfluenza Virus 3 (McCabe et al., 2018)**
**Sugar Beet (Beta vulgaris) (Minoche et al., 2015)**		**Pacific white shrimp (Litopenaeus vannamei) (Zhang et al., 2019b)**		**Human Immunodeficiency Virus (Bonsall et al., 2018)**
**Spinach (Spinacia oleracea) (Minoche et al., 2015)**		**Hummingbird (Archilochus colubris) (Workman et al., 2018a)**		**Maize chlorotic mottle virus (Adams et al., 2017)**
**Korean ginseng (Panax ginseng) (Jo et al., 2017)**		**Pacific Abalone (Haliotis discus hannai) (Kim et al., 2017)**		**Chikungunya virus (Greninger et al., 2015)**
**Populus Nanlin895 (Chao et al., 2018a)**				**Hepatitis C virus (Greninger et al., 2015)**

**Switchgrass (Panicum virgatum L.) (Zuo et al., 2018)**				
**Corn poppy (Papaver rhoeas) (Oh et al., 2018)**				
**Iceland poppy (Papaver nudicaule) (Oh et al., 2018)**				
**Rice seedling (Oryza sativa) (Zhang et al., 2019a)**				
**Arabica coffee bean (Coffea arabica) (Cheng et al., 2017)**				
**Zanthoxylum planispinum (Kim et al., 2018a)**				

**Nepenthes ampullaria (Zulkapli et al., 2017)**				
**Amborella trichopoda (Liu et al., 2017b)**				
**Picea abies (Akhter et al., 2018)**				.

Supplementary Table 2. Examples of organisms whose transcriptome or genome was sequenced on a long-read sequencing platform.

Supplementary figures

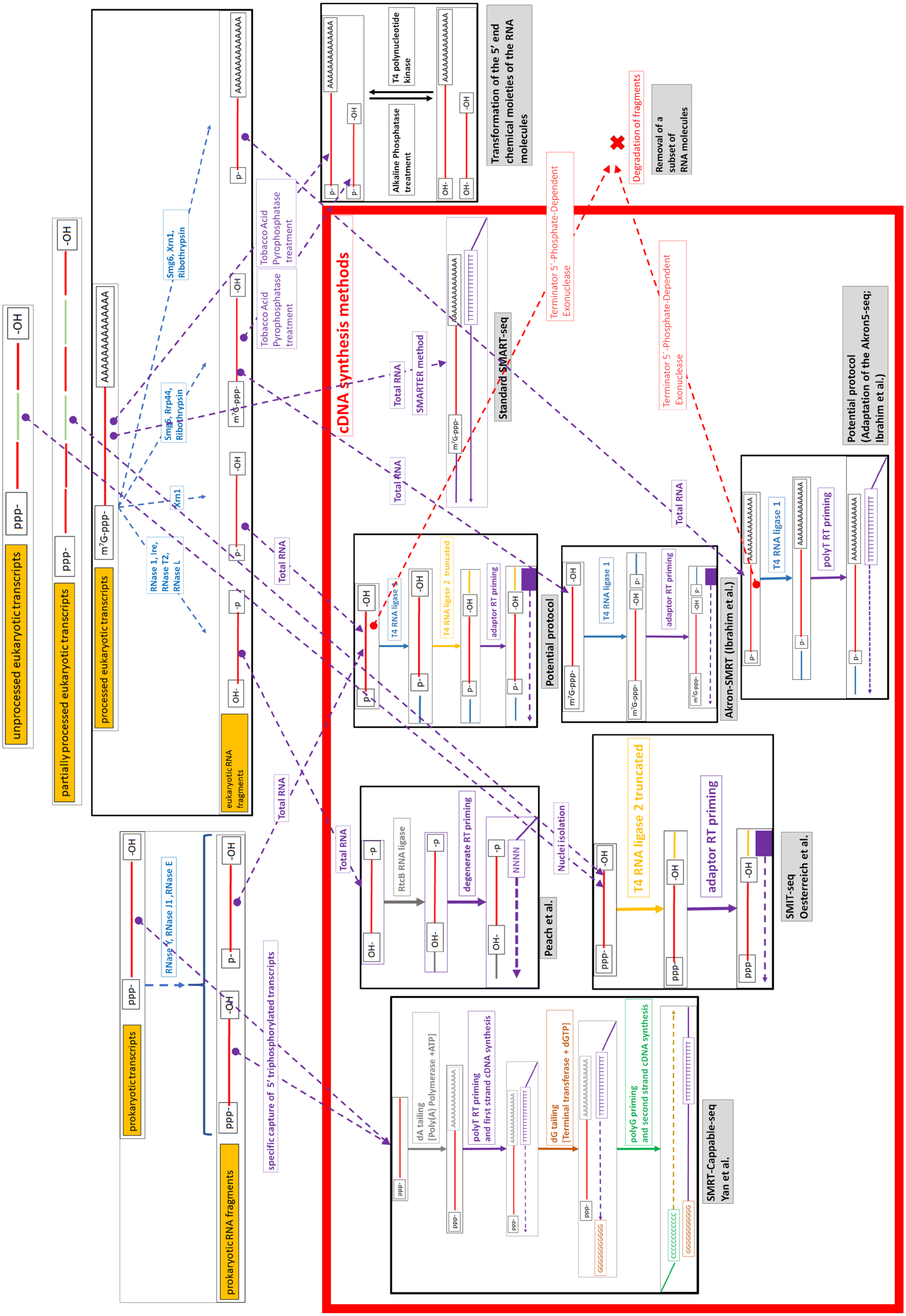

unprocessed eukaryotic transcripts
partially processed eukaryotic transcripts
processed eukaryotic transcripts
eukaryotic RNA fragments
prokaryotic transcripts
prokaryotic RNA fragments
RNase 1, Ire, RNase T2, RNase L
Xrn1
Smg6, Rrp44, Ribothrypsin
Smg6, Xrn1, Ribothrypsin
RNase Y, RNase J1, RNase E
specific capture of 5' triphosphorylated transcripts
Tobacco Acid Pyrophosphatase treatment
Transformation of the 5' end chemical moieties of the RNA molecules
Alkaline Phosphatase treatment
T4 polynucleotide kinase
Removal of a subset of RNA molecules
Degradation of fragments
Terminator 5'-Phosphate-Dependent Exonuclease
cDNA synthesis methods
Total RNA
SMARTER method
Standard SMART-seq
Potential protocol
T4 RNA ligase 1
T4 RNA ligase 2 truncated
adaptor RT priming
Akron-SMRT (Ibrahim et al.)
polyT RT priming
Potential protocol (Adaptation of the Akron5-seq; Ibrahim et al.)
RtcB RNA ligase
degenerate RT priming
Peach et al.
Nuclei isolation
SMIT-seq Oesterreich et al.
dA tailing [Poly(A) Polymerase +ATP]
polyT RT priming and first strand cDNA synthesis
dG tailing [Terminal transferase + dGTP]
polyG priming and second strand cDNA synthesis
SMRT-Cappable-seq Yan et al.

Supplementary Figure 1 (previous page). Sequence characteristics of RNA molecules or RNA fragments and their full-length cDNA synthesis methods. No RNA or cDNA fragmentation methods are followed in any of the presented cDNA synthesis methods. The dotted cyan lines correspond to RNA fragments produced from the processed eukaryotic mRNA transcripts of from the prokaryotic transcripts. The beginning and end of the dotted purple arrows indicate the same molecules at different places on the graph. The yellow, dark blue, grey short arrows indicate enzymatic reactions responsible for the addition of adaptors sequences on the RNA molecules/fragments from the truncated T4 RNA ligase 2, T4 RNA ligase 1 and RtcB RNA ligase respectively as indicated in the boxes. The black short arrows correspond to enzymatic reactions that are affecting the chemical moieties on the 5' end of RNA molecules/RNA fragments through the action of the Alkaline Phosphatase, Tobacco Acid Pyrophosphatase or T4 polynucleotide kinase as indicated inside the respective boxes. The dotted red lines indicate potential degradation of the fragments, present at the beginning of the arrow, through the action of the Terminator 5´-Phosphate-Dependent Exonuclease. In case that an "enzymatic reaction set" is not part of an already published protocol mentioned in the manuscript, this set is characterized as "Potential protocol". The methods presented here are from the following papers (Peach et al., 2015;Oesterreich et al., 2016;Ibrahim et al., 2018;Yan et al., 2018).

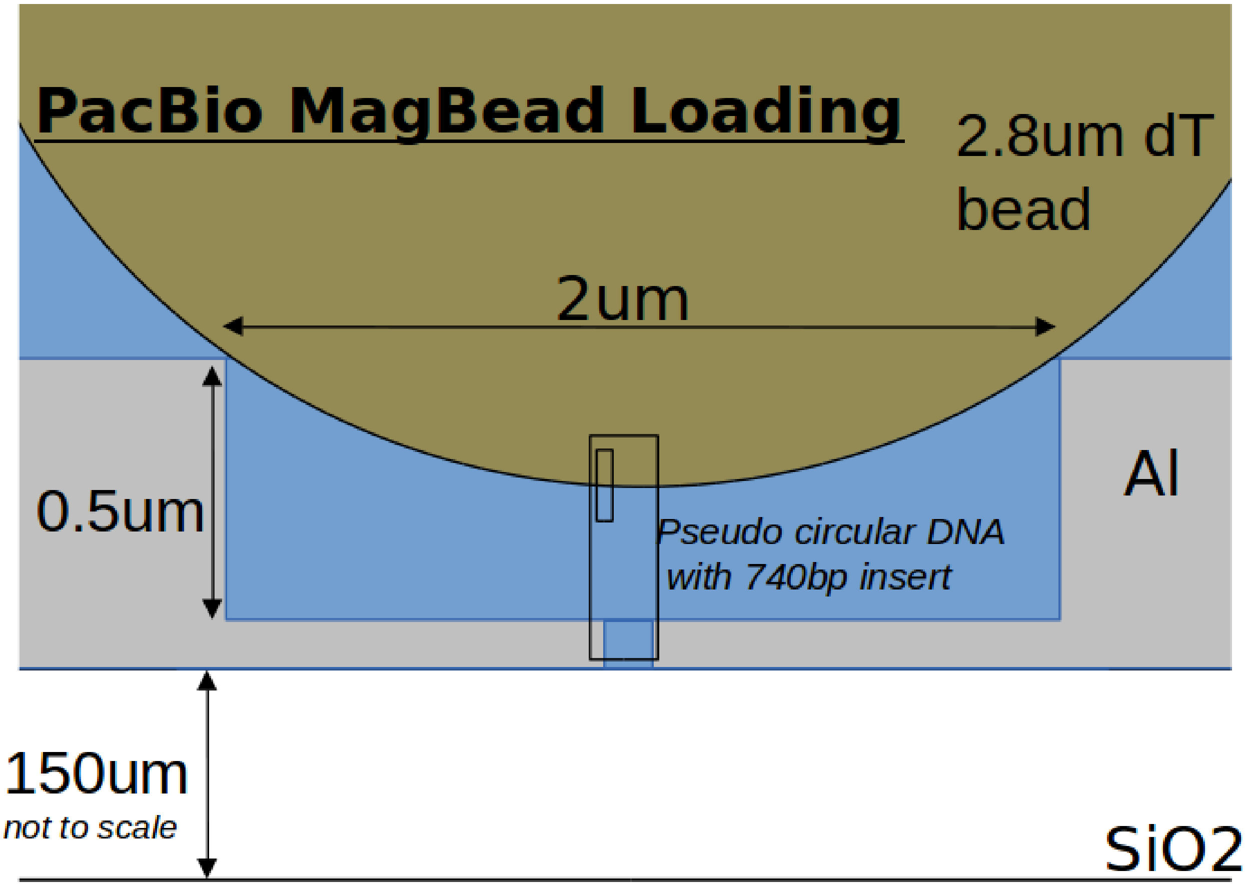

Supplementary Figure 2. MagBead loading on the ZMW flow cell. Dimensions of the MagBead and the ZMW well, are shown representing their relative positions once the beads are loaded on the flowcell.

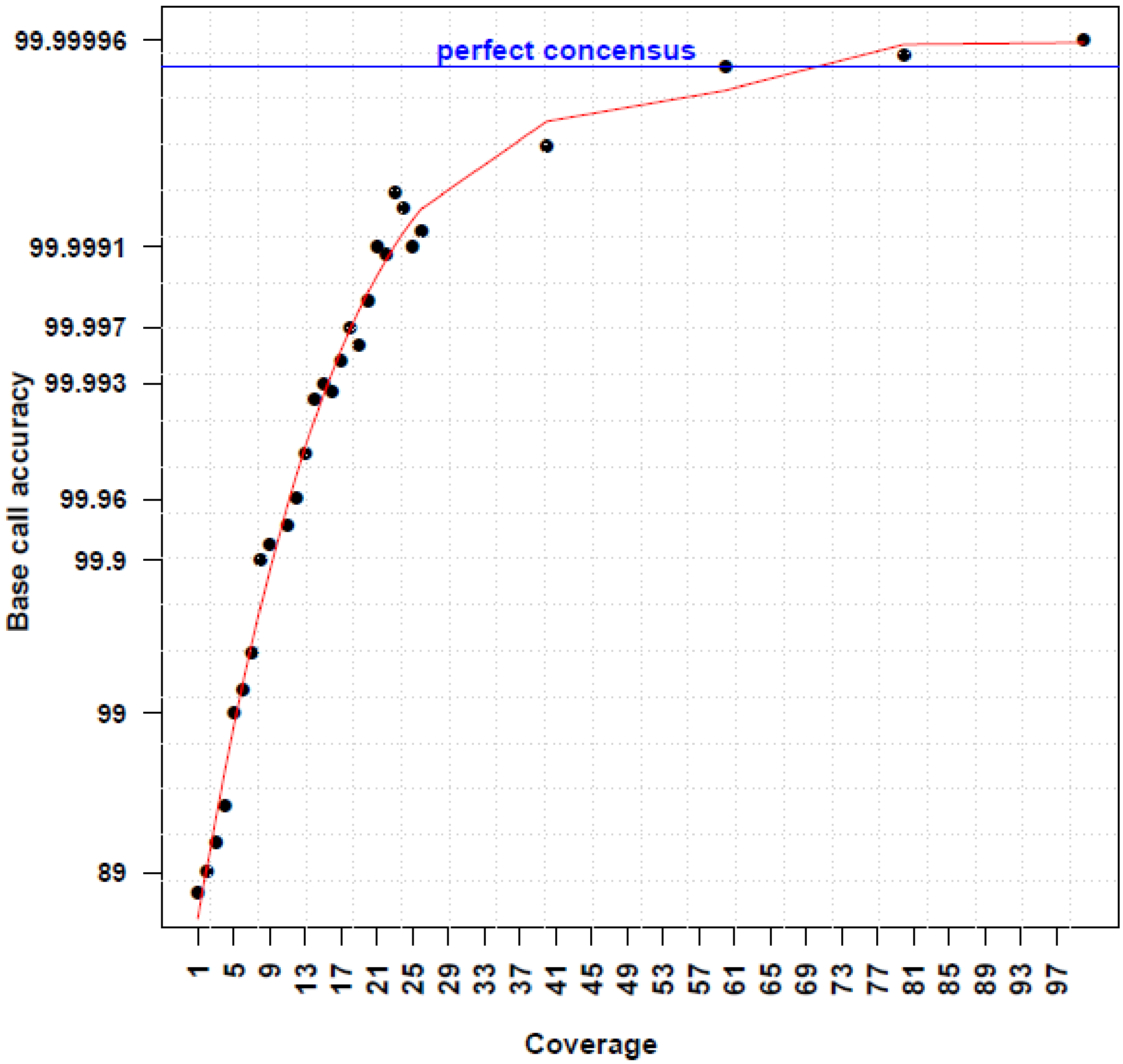

Supplementary Figure 3. Consensus accuracy as a function of long read sequencing coverage. The picture corresponds to the consensus accuracy recovered from the SMRT sequencing data. As the raw error rates between the Nanopore cDNA and PacBio cDNA platforms are not significantly different, the base call accuracy versus coverage plot should be approximately similar. We note here that the indicated improvement in the base calling accuracy is due to either the pile up of individually sequenced reads on the same locus or the pile up of the subreads from the PacBio Circular Consensus sequencing and by extrapolation from the R2C2 (Volden et al., 2018) or from the INC-seq (Li et al., 2016) nanopore based methods. The improvement in accuracy does not correspond to joint basecalling methods such as the ONT 2D sequencing protocol or the ONT 1D^2 sequencing protocol. The picture was produced after merging the reported consensus accuracy metrics for the PacBio data from similar plots publicly available on the PacBio website (PacBio_website _1, 2013;PacBio_website _2, 2015).

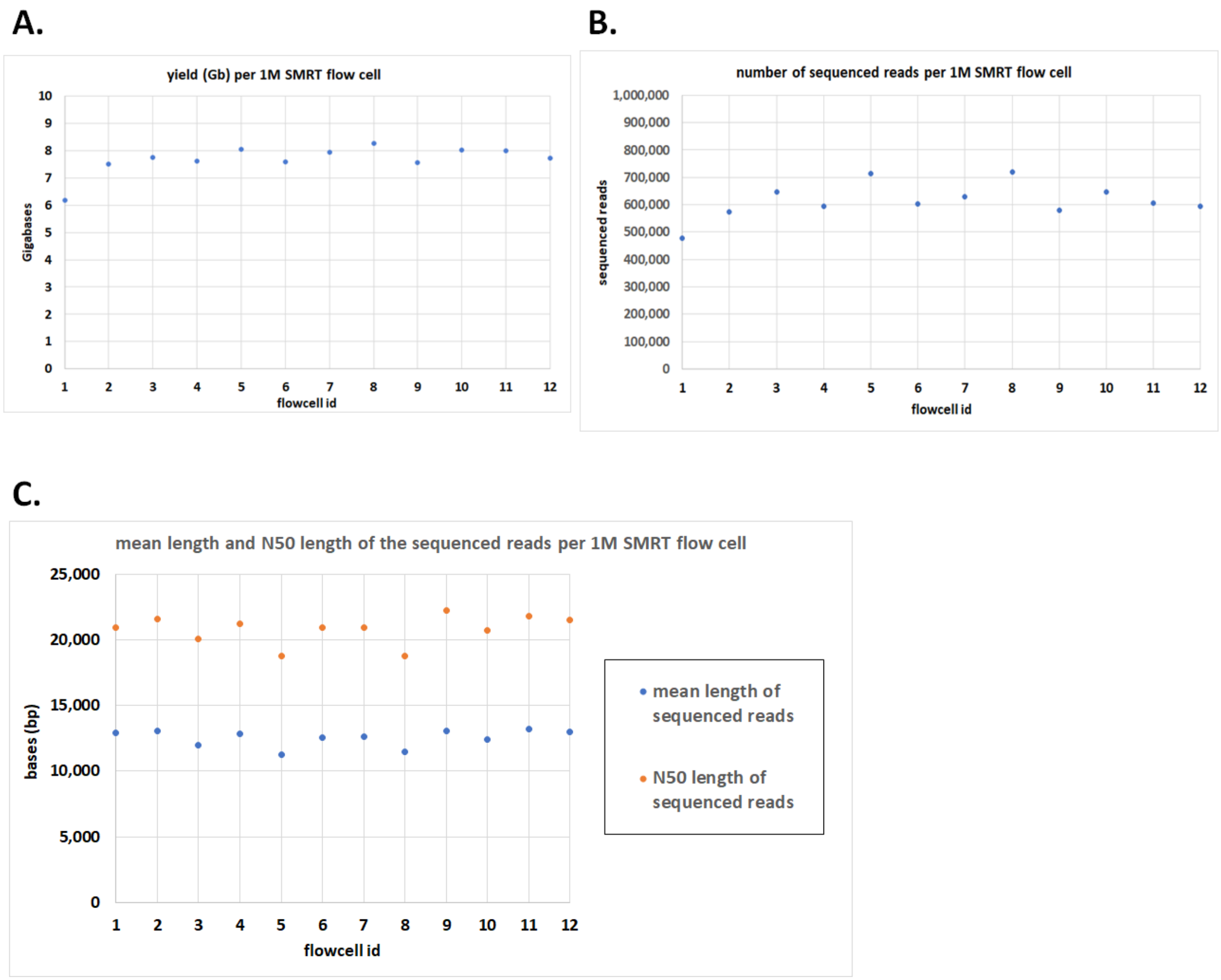

Supplementary Figure 4. PacBio Sequel SMRT sequencing run statistics for the 1M SMRT cell from fragmented genomic DNA samples. The PacBio Sequel v3.0 chemistry was used. A) Yield per 1M SMRT cell. The y axis corresponds to Gigabases produced. B) Number of sequenced reads per 1M SMRT cell. C) Mean read length and N50 length of the sequenced reads. Given the circular nature of sequencing, the sequencing run statistics from cDNA samples should be at least as good as the ones presented here.

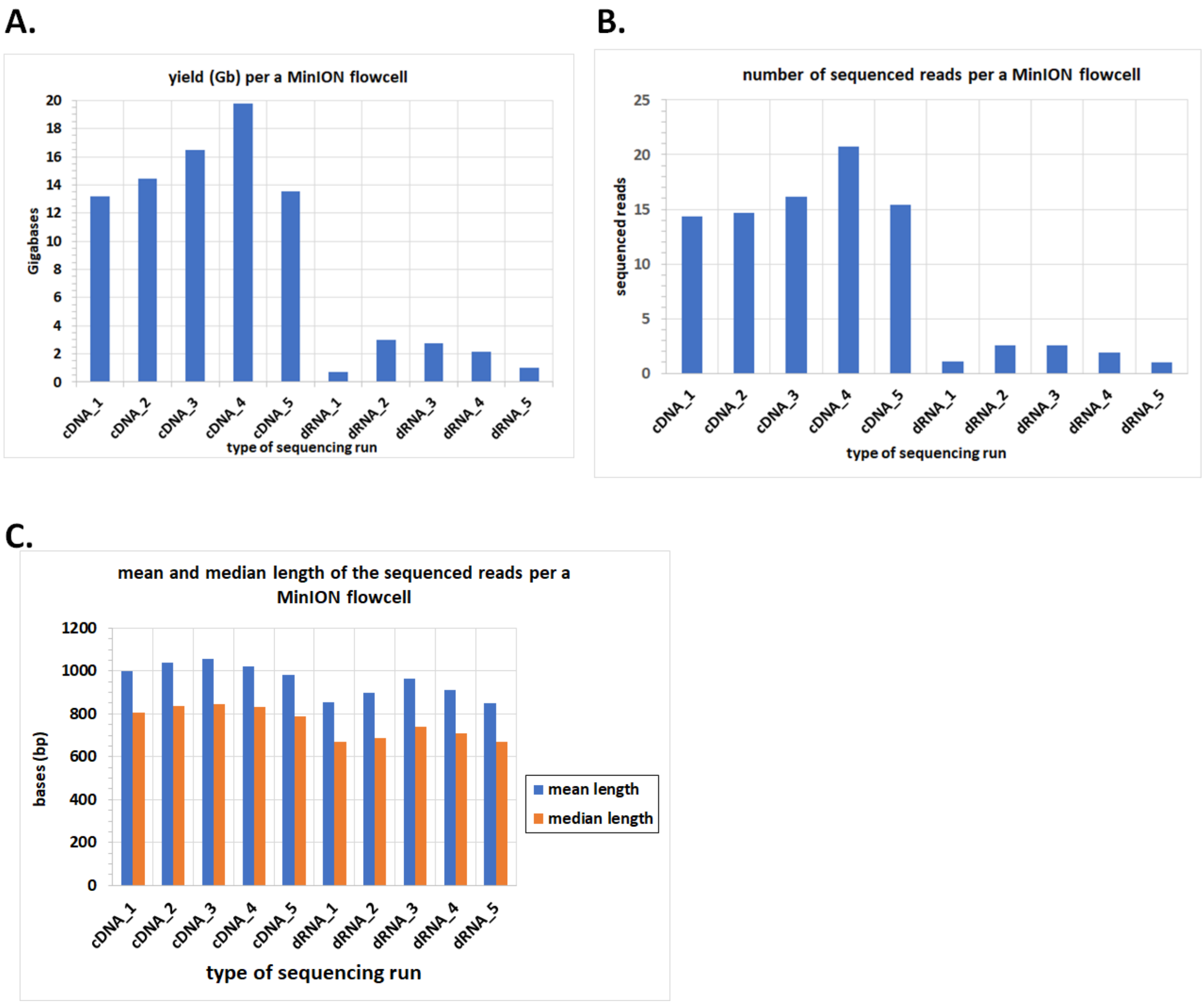

Supplementary Figure 5. Sequencing run statistics for the ONT MinION from amplified cDNA and direct RNA experiments. A) Yield per ONT MinION flowcell. The y axis corresponds to Gigabases produced. B) Number of sequenced reads per ONT MinION flowcell. C) Mean and median read length of the sequenced reads per ONT MinION flowcell. Each position on the x axis is a different MinION run. The amplified cDNA sequencing runs are presented as "cDNA_X" and the direct RNA sequencing runs are presented as "dRNA_X" where "X" is the number of a specific sample. The RNA from five different samples (mouse embryonic stem cells) were sequenced on the MinION with both an amplified cDNA and a direct RNA protocol. cDNA and direct RNA runs with the same "X" number correspond to the same sample. For the cDNA runs the sequencing chemistry used is the "SQK-PCS109" whereas for the direct RNA runs it is the "SQK-RNA001". The nanopore version used is the "v9.4.1". In the SQK-PCS109 chemistry the attachment of the sequencing adaptors on the cDNA molecules is performed through a click chemistry reaction.

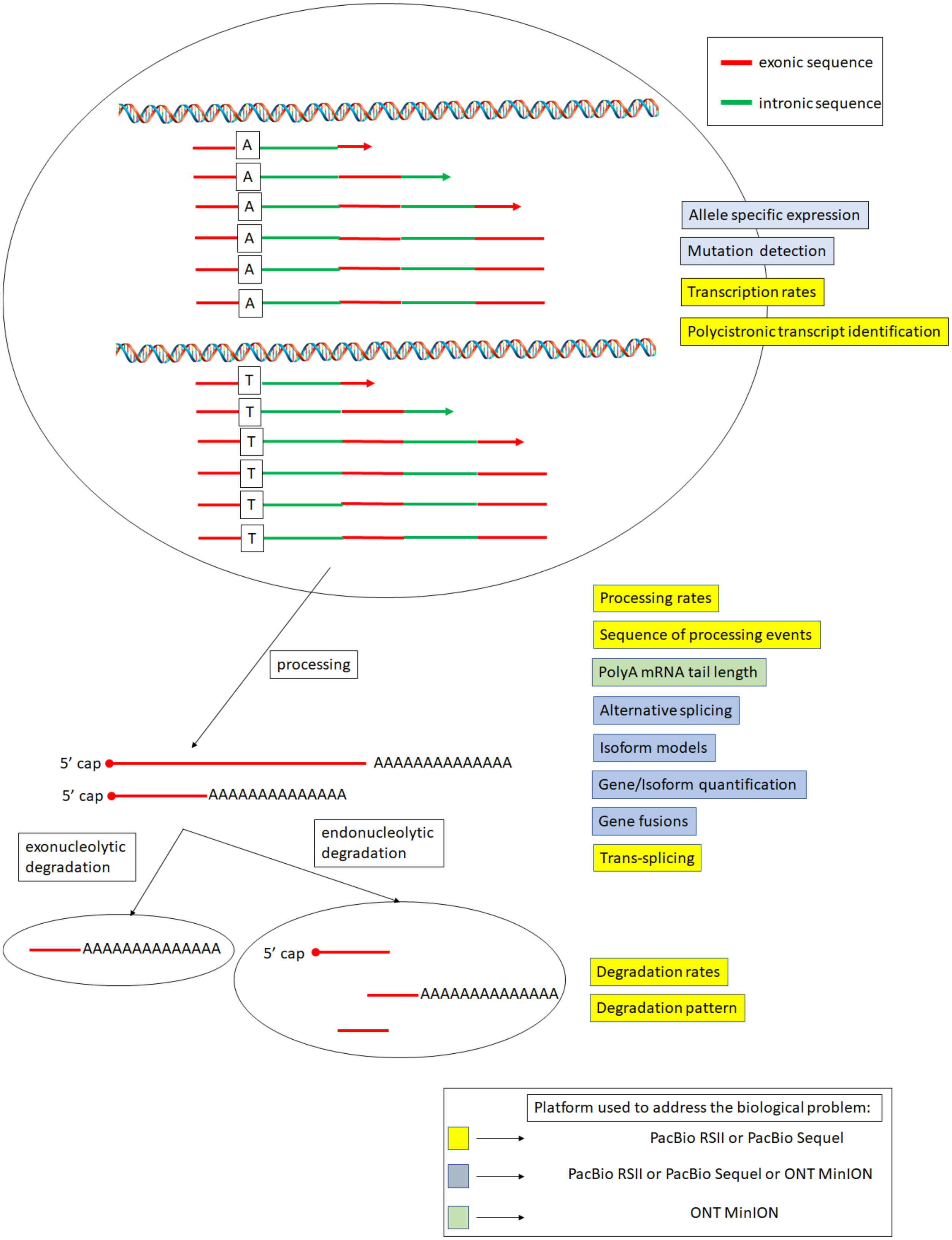

Supplementary Figure 6. Biological problems addressed with the long-read sequencing platforms relevant to RNA sequencing. The colored boxes present the problem addressed. As indicated in the bottom legend, the different colors correspond to the platform used to address the

biological question. The red horizontal lines and mixed red/green horizontal lines correspond to fully processed and unprocessed mRNA molecules respectively.

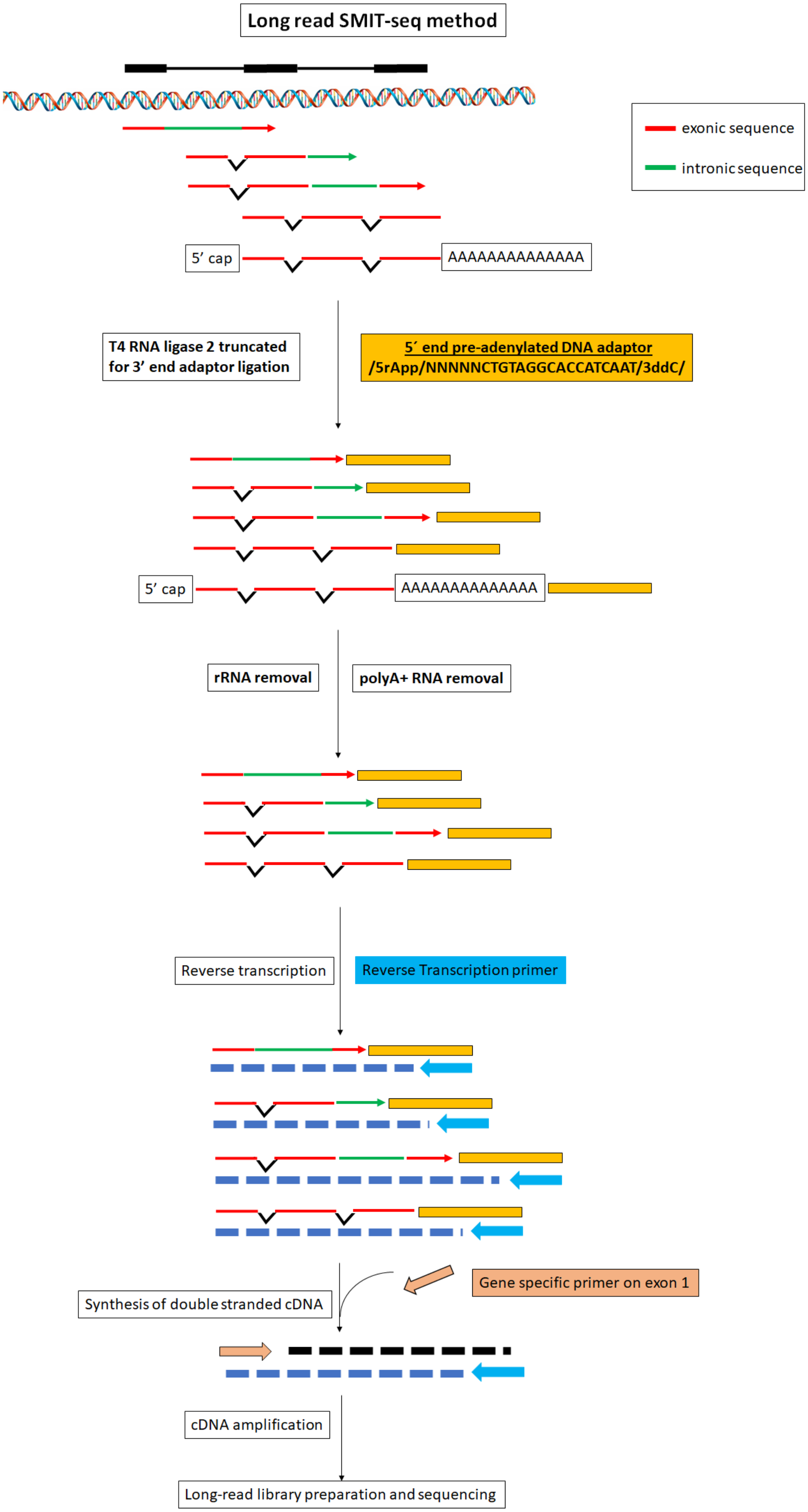

Long read SMIT-seq method
exonic sequence
intronic sequence
5′ cap
AAAAAAAAAAAAAA
T4 RNA ligase 2 truncated for 3′ end adaptor ligation
5′ end pre-adenylated DNA adaptor /5rApp/NNNNNCTGTAGGCACCATCAAT/3ddC/
5′ cap
AAAAAAAAAAAAAA
rRNA removal
polyA+ RNA removal
Reverse transcription
Reverse Transcription primer
Synthesis of double stranded cDNA
Gene specific primer on exon 1
cDNA amplification
Long-read library preparation and sequencing

Supplementary Figure 7 (previous page). Identification of processing patterns with long-read sequencing. The long read SMIT-seq method (Oesterreich et al., 2016) is presented here. The aim of this method is to sequence unprocessed and partially processed non poly-adenylated transcripts. The protocol has the following steps as presented in the figure. Initially, cell lysis is followed by chromatin isolation. The nascent RNA is extracted from the chromatin fraction with the acidic Phenol/Chloroform method followed by DNAse I treatment. A 3' end ligation is performed with a truncated T4 RNA ligase 2 that adds a 3′ end linker (orange box) to the 3' end of all the RNA molecules. Non poly-adenylated RNA is obtained after depletion of poly-adenylated RNA with oligo-dT coated beads. Then, rRNA removal is performed. The nascent polyA minus RNA is reversed transcribed with a custom primer hybridizing on the linker (cyan arrow). Double-stranded DNA is generated with gene-specific primers (pink arrow) in order to increase sequence read counts for a specific set of genes. Low PCR cycles cDNA amplification and long read sequencing is followed at the end.

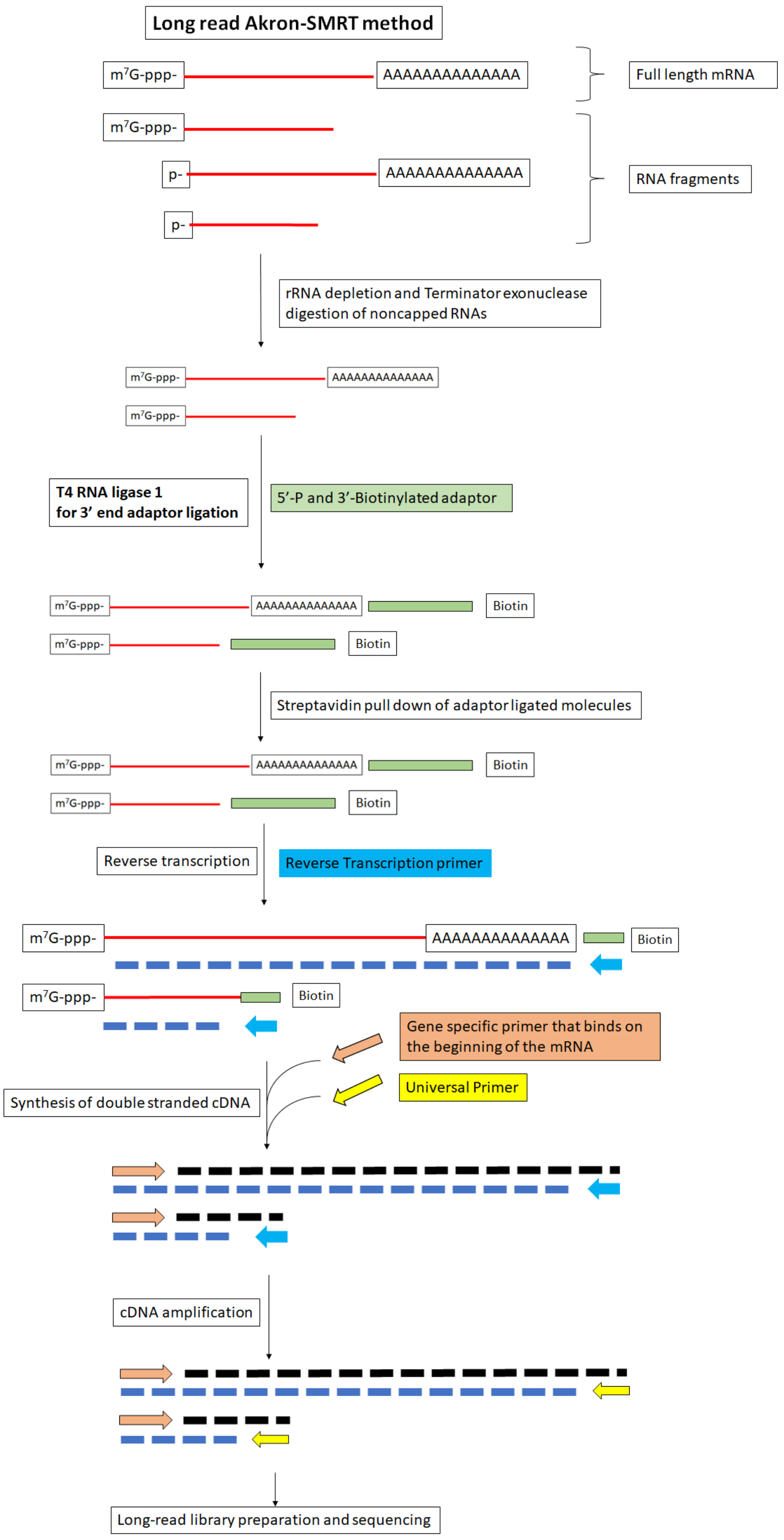
Long read Akron-SMRT method
m7G-ppp-
AAAAAAAAAAAAAA
Full length mRNA
p-
RNA fragments
rRNA depletion and Terminator exonuclease digestion of noncapped RNAs
T4 RNA ligase 1 for 3' end adaptor ligation
5'-P and 3'-Biotinylated adaptor
Biotin
Streptavidin pull down of adaptor ligated molecules
Reverse transcription
Reverse Transcription primer
Gene specific primer that binds on the beginning of the mRNA
Universal Primer
Synthesis of double stranded cDNA
cDNA amplification
Long-read library preparation and sequencing

Supplementary Figure 8 (previous page). Identification of degradation patterns with long-read sequencing. The long-read Akron-SMRT method (Ibrahim et al., 2018) is presented here. The aim of this method is to sequence degradation fragments of RNA molecules and define the exact position of the endonucleolytic cut on the 5' end fragments of the RNA molecules. The method has been used to profile the degradation patterns of the mRNA molecules occurred during their translation on the polysomes. The protocol has the following steps presented in the figure. Total RNA is extracted from the polysome fraction with Trizol followed by rRNA depletion, snRNA depletion and short fragment removal (<200bp). To sequence the degradation fragments derived from the 5' end of the mRNA molecules, the RNA is treated with Terminator exonuclease to remove non-capped, 5′-P-bearing RNAs. The 3' end of the remaining mRNA fragments is then ligated to a 5'-P and 3′-biotinylated RNA adaptor (green box) with a T4 RNA ligase 1, followed by streptavidin pulldown. cDNA synthesis is performed with a primer that binds on the added adaptor sequence (cyan arrow). Double-stranded DNA is generated with gene-specific primers (pink arrow) in order to increase sequence read counts for a specific set of genes. A universal primer (yellow arrow) is used for cDNA amplification followed by long read sequencing.

References

Abdel-Ghany, S.E., Hamilton, M., Jacobi, J.L., Ngam, P., Devitt, N., Schilkey, F., Ben-Hur, A., and Reddy, A.S. (2016). A survey of the sorghum transcriptome using single-molecule long reads. *Nat Commun* 7**,** 11706.

Adams, I., Braidwood, L., Stomeo, F., Phiri, N., Uwumukiza, B., Feyissa, B., Mahuku, G., Wangai, A., Smith, J., Mumford, R., and Boonham, N. (2017). Characterising maize viruses associated with maize lethal necrosis symptoms in sub Saharan Africa. *bioRxiv***,** 161489.

Akhter, S., Kretzschmar, W.W., Nordal, V., Delhomme, N., Street, N.R., Nilsson, O., Emanuelsson, O., and Sundstrom, J.F. (2018). Integrative Analysis of Three RNA Sequencing Methods Identifies Mutually Exclusive Exons of MADS-Box Isoforms During Early Bud Development in Picea abies. *Front Plant Sci* 9**,** 1625.

Asur, R.S., Kimble, D.C., Lach, F.P., Jung, M., Donovan, F.X., Kamat, A., Noonan, R.J., Thomas, J.W., Park, M., Chines, P., Vlachos, A., Auerbach, A.D., Smogorzewska, A., and Chandrasekharappa, S.C. (2018). Somatic mosaicism of an intragenic FANCB duplication in both fibroblast and peripheral blood cells observed in a Fanconi anemia patient leads to milder phenotype. *Mol Genet Genomic Med* 6**,** 77-91.

Balazs, Z., Tombacz, D., Szucs, A., Snyder, M., and Boldogkoi, Z. (2018). Dual Platform Long-Read RNA-Sequencing Dataset of the Human Cytomegalovirus Lytic Transcriptome. *Front Genet* 9**,** 432.

Barth, S., Pfuhl, T., Mamiani, A., Ehses, C., Roemer, K., Kremmer, E., Jaker, C., Hock, J., Meister, G., and Grasser, F.A. (2008). Epstein-Barr virus-encoded microRNA miR-BART2 down-regulates the viral DNA polymerase BALF5. *Nucleic Acids Res* 36**,** 666-675.

Bayega, A., Oikonomopoulos, S., Zorbas, E., Wang, Y.C., Gregoriou, M.-E., Tsoumani, K.T., Mathiopoulos, K.D., and Ragoussis, J. (2018). Transcriptome landscape of the developing olive fruit fly embryo delineated by Oxford Nanopore long-read RNA-Seq. *bioRxiv***,** 478172.

Bevilacqua, P.C., Ritchey, L.E., Su, Z., and Assmann, S.M. (2016). Genome-Wide Analysis of RNA Secondary Structure. *Annu Rev Genet* 50**,** 235-266.

Boldogkoi, Z. (2012). Transcriptional interference networks coordinate the expression of functionally related genes clustered in the same genomic loci. *Front Genet* 3**,** 122.

Boldogkői, Z., Moldován, N., Szűcs, A., and Tombácz, D. (2018a). Transcriptome-wide analysis of a baculovirus using nanopore sequencing. *Scientific Data* 5**,** 180276.

Boldogkői, Z., Szűcs, A., Balázs, Z., Sharon, D., Snyder, M., and Tombácz, D. (2018b). Transcriptomic study of Herpes simplex virus type-1 using full-length sequencing techniques. *Scientific Data* 5**,** 180266.

Bolisetty, M.T., Rajadinakaran, G., and Graveley, B.R. (2015). Determining exon connectivity in complex mRNAs by nanopore sequencing. *Genome Biol* 16**,** 204.

Bonsall, D., Golubchik, T., De Cesare, M., Limbada, M., Kosloff, B., Macintyre-Cockett, G., Hall, M., Wymant, C., Ansari, A., Abeler-Dorner, L., Schaap, A., Brown, A., Barnes, E., Piwowar-Manning, E., Wilson, E., Emel, L., Hayes, R., Fidler, S., Ayles, H., Bowden, R., and Fraser, C. (2018). A comprehensive genomics solution for HIV surveillance and clinical monitoring in a global health setting. *bioRxiv***,** 397083.

Bronzato Badial, A., Sherman, D., Stone, A., Gopakumar, A., Wilson, V., Schneider, W., and King, J. (2018). Nanopore Sequencing as a Surveillance Tool for Plant Pathogens in Plant and Insect Tissues. *Plant Dis* 102**,** 1648-1652.

Brown, J.B., Boley, N., Eisman, R., May, G.E., Stoiber, M.H., Duff, M.O., Booth, B.W., Wen, J., Park, S., Suzuki, A.M., Wan, K.H., Yu, C., Zhang, D., Carlson, J.W., Cherbas, L., Eads, B.D., Miller, D., Mockaitis, K., Roberts, J., Davis, C.A., Frise, E., Hammonds, A.S., Olson, S., Shenker, S., Sturgill, D., Samsonova, A.A., Weiszmann, R., Robinson, G., Hernandez, J., Andrews, J., Bickel, P.J., Carninci,

P., Cherbas, P., Gingeras, T.R., Hoskins, R.A., Kaufman, T.C., Lai, E.C., Oliver, B., Perrimon, N., Graveley, B.R., and Celniker, S.E. (2014). Diversity and dynamics of the Drosophila transcriptome. *Nature* 512**,** 393-399.

Bull, R.A., Eltahla, A.A., Rodrigo, C., Koekkoek, S.M., Walker, M., Pirozyan, M.R., Betz-Stablein, B., Toepfer, A., Laird, M., Oh, S., Heiner, C., Maher, L., Schinkel, J., Lloyd, A.R., and Luciani, F. (2016). A method for near full-length amplification and sequencing for six hepatitis C virus genotypes. *BMC Genomics* 17**,** 247.

Butt, S.L., Taylor, T.L., Volkening, J., Dimitrov, K.M., Williams-Coplin, D., Lahmers, K.K., Rana, A.M., Suarez, D.L., Afonso, C.L., and Stanton, J.B. (2018). Rapid and sensitive virulence prediction and identification of Newcastle disease virus genotypes using third-generation sequencing. *bioRxiv***,** 349159.

Cavelier, L., Ameur, A., Haggqvist, S., Hoijer, I., Cahill, N., Olsson-Stromberg, U., and Hermanson, M. (2015). Clonal distribution of BCR-ABL1 mutations and splice isoforms by single-molecule long-read RNA sequencing. *BMC Cancer* 15**,** 45.

Chao, Q., Gao, Z.F., Zhang, D., Zhao, B.G., Dong, F.Q., Fu, C.X., Liu, L.J., and Wang, B.C. (2018a). The developmental dynamics of the Populus stem transcriptome. *Plant Biotechnol J*.

Chao, Y., Yuan, J., Guo, T., Xu, L., Mu, Z., and Han, L. (2019). Analysis of transcripts and splice isoforms in Medicago sativa L. by single-molecule long-read sequencing. *Plant Mol Biol* 99**,** 219-235.

Chao, Y., Yuan, J., Li, S., Jia, S., Han, L., and Xu, L. (2018b). Analysis of Transcripts and splice isoforms in Red Clover (Trifolium pratense L.) by single-molecule long-read sequencing. *bioRxiv*.

Chen, S.Y., Deng, F., Jia, X., Li, C., and Lai, S.J. (2017). A transcriptome atlas of rabbit revealed by PacBio single-molecule long-read sequencing. *Sci Rep* 7**,** 7648.

Chen, Y., Wu, Y., Liu, L., Feng, J., Zhang, T., Qin, S., Zhao, X., Wang, C., Li, D., Han, W., Shao, M., Zhao, P., Xue, J., Liu, X., Li, H., Zhao, E., Zhao, W., Guo, X., Jin, Y., Cao, Y., Cui, L., Zhou, Z., Xia, Q., Rao, Z., and Zhang, Y. (2019). Study of the whole genome, methylome and transcriptome of Cordyceps militaris. *Sci Rep* 9**,** 898.

Cheng, B., Furtado, A., and Henry, R.J. (2017). Long-read sequencing of the coffee bean transcriptome reveals the diversity of full-length transcripts. *Gigascience* 6**,** 1-13.

Clark, M., Wrzesinski, T., Garcia-Bea, A., Kleinman, J., Hyde, T., Weinberger, D., Haerty, W., and Tunbridge, E. (2018). Long-read sequencing reveals the splicing profile of the calcium channel gene CACNA1C in human brain. *bioRxiv***,** 260562.

Cook, D.E., Valle-Inclan, J.E., Pajoro, A., Rovenich, H., Thomma, B., and Faino, L. (2019). Long-Read Annotation: Automated Eukaryotic Genome Annotation Based on Long-Read cDNA Sequencing. *Plant Physiol* 179**,** 38-54.

De Jong, L.C., Cree, S., Lattimore, V., Wiggins, G.a.R., Spurdle, A.B., Kconfab, I., Miller, A., Kennedy, M.A., and Walker, L.C. (2017). Nanopore sequencing of full-length BRCA1 mRNA transcripts reveals co-occurrence of known exon skipping events. *Breast Cancer Res* 19**,** 127.

De Roeck, A., Van Den Bossche, T., Van Der Zee, J., Verheijen, J., De Coster, W., Van Dongen, J., Dillen, L., Baradaran-Heravi, Y., Heeman, B., Sanchez-Valle, R., Llado, A., Nacmias, B., Sorbi, S., Gelpi, E., Grau-Rivera, O., Gomez-Tortosa, E., Pastor, P., Ortega-Cubero, S., Pastor, M.A., Graff, C., Thonberg, H., Benussi, L., Ghidoni, R., Binetti, G., De Mendonca, A., Martins, M., Borroni, B., Padovani, A., Almeida, M.R., Santana, I., Diehl-Schmid, J., Alexopoulos, P., Clarimon, J., Lleo, A., Fortea, J., Tsolaki, M., Koutroumani, M., Matej, R., Rohan, Z., De Deyn, P., Engelborghs, S., Cras, P., Van Broeckhoven, C., Sleegers, K., and European Early-Onset Dementia, C. (2017). Deleterious ABCA7 mutations and transcript rescue mechanisms in early onset Alzheimer's disease. *Acta Neuropathol* 134**,** 475-487.

Deininger, P., Morales, M.E., White, T.B., Baddoo, M., Hedges, D.J., Servant, G., Srivastav, S., Smither, M.E., Concha, M., Deharo, D.L., Flemington, E.K., and Belancio, V.P. (2017). A comprehensive approach to expression of L1 loci. *Nucleic Acids Res* 45**,** e31.

Depledge, D.P., Kalanghad Puthankalam, S., Sadaoka, T., Beady, D., Mori, Y., Placantonakis, D., Mohr, I., and Wilson, A. (2018). Native RNA sequencing on nanopore arrays redefines the transcriptional complexity of a viral pathogen. *bioRxiv*, 373522.

Depledge, D.P., Mohr, I., and Wilson, A.C. (2019). Going the Distance: Optimizing RNA-Seq Strategies for Transcriptomic Analysis of Complex Viral Genomes. *J Virol* 93.

Derrien, T., Johnson, R., Bussotti, G., Tanzer, A., Djebali, S., Tilgner, H., Guernec, G., Martin, D., Merkel, A., Knowles, D.G., Lagarde, J., Veeravalli, L., Ruan, X., Ruan, Y., Lassmann, T., Carninci, P., Brown, J.B., Lipovich, L., Gonzalez, J.M., Thomas, M., Davis, C.A., Shiekhattar, R., Gingeras, T.R., Hubbard, T.J., Notredame, C., Harrow, J., and Guigo, R. (2012). The GENCODE v7 catalog of human long noncoding RNAs: analysis of their gene structure, evolution, and expression. *Genome Res* 22**,** 1775-1789.

Dickson, A.M., Anderson, J.R., Barnhart, M.D., Sokoloski, K.J., Oko, L., Opyrchal, M., Galanis, E., Wilusz, C.J., Morrison, T.E., and Wilusz, J. (2012). Dephosphorylation of HuR protein during alphavirus infection is associated with HuR relocalization to the cytoplasm. *J Biol Chem* 287**,** 36229-36238.

Dong, L., Liu, H., Zhang, J., Yang, S., Kong, G., Chu, J.S., Chen, N., and Wang, D. (2015). Single-molecule real-time transcript sequencing facilitates common wheat genome annotation and grain transcriptome research. *BMC Genomics* 16**,** 1039.

Dougherty, M.L., Underwood, J.G., Nelson, B.J., Tseng, E., Munson, K.M., Penn, O., Nowakowski, T.J., Pollen, A.A., and Eichler, E.E. (2018). Transcriptional fates of human-specific segmental duplications in brain. *Genome Res* 28**,** 1566-1576.

Duchaine, T.F., and Fabian, M.R. (2019). Mechanistic Insights into MicroRNA-Mediated Gene Silencing. *Cold Spring Harb Perspect Biol* 11.

Durkin, K., Rosewick, N., Artesi, M., Hahaut, V., Griebel, P., Arsic, N., Burny, A., Georges, M., and Van Den Broeke, A. (2016). Characterization of novel Bovine Leukemia Virus (BLV) antisense transcripts by deep sequencing reveals constitutive expression in tumors and transcriptional interaction with viral microRNAs. *Retrovirology* 13**,** 33.

Eckert, S.E., Chan, J.Z., Houniet, D., Consortium, P., Breuer, J., and Speight, G. (2016). Enrichment by hybridisation of long DNA fragments for Nanopore sequencing. *Microb Genom* 2**,** e000087.

Faria, N.R., Kraemer, M.U.G., Hill, S.C., Goes De Jesus, J., Aguiar, R.S., Iani, F.C.M., Xavier, J., Quick, J., Du Plessis, L., Dellicour, S., Theze, J., Carvalho, R.D.O., Baele, G., Wu, C.H., Silveira, P.P., Arruda, M.B., Pereira, M.A., Pereira, G.C., Lourenco, J., Obolski, U., Abade, L., Vasylyeva, T.I., Giovanetti, M., Yi, D., Weiss, D.J., Wint, G.R.W., Shearer, F.M., Funk, S., Nikolay, B., Fonseca, V., Adelino, T.E.R., Oliveira, M.a.A., Silva, M.V.F., Sacchetto, L., Figueiredo, P.O., Rezende, I.M., Mello, E.M., Said, R.F.C., Santos, D.A., Ferraz, M.L., Brito, M.G., Santana, L.F., Menezes, M.T., Brindeiro, R.M., Tanuri, A., Dos Santos, F.C.P., Cunha, M.S., Nogueira, J.S., Rocco, I.M., Da Costa, A.C., Komninakis, S.C.V., Azevedo, V., Chieppe, A.O., Araujo, E.S.M., Mendonca, M.C.L., Dos Santos, C.C., Dos Santos, C.D., Mares-Guia, A.M., Nogueira, R.M.R., Sequeira, P.C., Abreu, R.G., Garcia, M.H.O., Abreu, A.L., Okumoto, O., Kroon, E.G., De Albuquerque, C.F.C., Lewandowski, K., Pullan, S.T., Carroll, M., De Oliveira, T., Sabino, E.C., Souza, R.P., Suchard, M.A., Lemey, P., Trindade, G.S., Drumond, B.P., Filippis, A.M.B., Loman, N.J., Cauchemez, S., Alcantara, L.C.J., and Pybus, O.G. (2018). Genomic and epidemiological monitoring of yellow fever virus transmission potential. *Science* 361**,** 894-899.

Faria, N.R., Quick, J., Claro, I.M., Theze, J., De Jesus, J.G., Giovanetti, M., Kraemer, M.U.G., Hill, S.C., Black, A., Da Costa, A.C., Franco, L.C., Silva, S.P., Wu, C.H., Raghwani, J., Cauchemez, S., Du Plessis, L., Verotti, M.P., De Oliveira, W.K., Carmo, E.H., Coelho, G.E., Santelli, A., Vinhal, L.C., Henriques, C.M., Simpson, J.T., Loose, M., Andersen, K.G., Grubaugh, N.D., Somasekar, S., Chiu, C.Y., Munoz-Medina, J.E., Gonzalez-Bonilla, C.R., Arias, C.F., Lewis-Ximenez, L.L., Baylis, S.A., Chieppe, A.O., Aguiar, S.F., Fernandes, C.A., Lemos, P.S., Nascimento, B.L.S., Monteiro, H.a.O., Siqueira, I.C., De Queiroz, M.G., De Souza, T.R., Bezerra, J.F., Lemos, M.R., Pereira, G.F., Loudal, D., Moura, L.C., Dhalia, R., Franca, R.F., Magalhaes, T., Marques, E.T., Jr., Jaenisch, T., Wallau,

G.L., De Lima, M.C., Nascimento, V., De Cerqueira, E.M., De Lima, M.M., Mascarenhas, D.L., Neto, J.P.M., Levin, A.S., Tozetto-Mendoza, T.R., Fonseca, S.N., Mendes-Correa, M.C., Milagres, F.P., Segurado, A., Holmes, E.C., Rambaut, A., Bedford, T., Nunes, M.R.T., Sabino, E.C., Alcantara, L.C.J., Loman, N.J., and Pybus, O.G. (2017). Establishment and cryptic transmission of Zika virus in Brazil and the Americas. *Nature* 546**,** 406-410.

Fiddes, I.T., Lodewijk, G.A., Mooring, M., Bosworth, C.M., Ewing, A.D., Mantalas, G.L., Novak, A.M., Van Den Bout, A., Bishara, A., Rosenkrantz, J.L., Lorig-Roach, R., Field, A.R., Haeussler, M., Russo, L., Bhaduri, A., Nowakowski, T.J., Pollen, A.A., Dougherty, M.L., Nuttle, X., Addor, M.C., Zwolinski, S., Katzman, S., Kriegstein, A., Eichler, E.E., Salama, S.R., Jacobs, F.M.J., and Haussler, D. (2018). Human-Specific NOTCH2NL Genes Affect Notch Signaling and Cortical Neurogenesis. *Cell* 173**,** 1356-1369 e1322.

Gao, Q., Liang, W.W., Foltz, S.M., Mutharasu, G., Jayasinghe, R.G., Cao, S., Liao, W.W., Reynolds, S.M., Wyczalkowski, M.A., Yao, L., Yu, L., Sun, S.Q., Fusion Analysis Working, G., Cancer Genome Atlas Research, N., Chen, K., Lazar, A.J., Fields, R.C., Wendl, M.C., Van Tine, B.A., Vij, R., Chen, F., Nykter, M., Shmulevich, I., and Ding, L. (2018a). Driver Fusions and Their Implications in the Development and Treatment of Human Cancers. *Cell Rep* 23**,** 227-238 e223.

Gao, S., Tian, X., Chang, H., Sun, Y., Wu, Z., Cheng, Z., Dong, P., Zhao, Q., Ruan, J., and Bu, W. (2018b). Two novel lncRNAs discovered in human mitochondrial DNA using PacBio full-length transcriptome data. *Mitochondrion* 38**,** 41-47.

Gao, Y., Wang, H., Zhang, H., Wang, Y., Chen, J., and Gu, L. (2018c). PRAPI: post-transcriptional regulation analysis pipeline for Iso-Seq. *Bioinformatics* 34**,** 1580-1582.

Greninger, A.L., Naccache, S.N., Federman, S., Yu, G., Mbala, P., Bres, V., Stryke, D., Bouquet, J., Somasekar, S., Linnen, J.M., Dodd, R., Mulembakani, P., Schneider, B.S., Muyembe-Tamfum, J.J., Stramer, S.L., and Chiu, C.Y. (2015). Rapid metagenomic identification of viral pathogens in clinical samples by real-time nanopore sequencing analysis. *Genome Med* 7**,** 99.

Grubaugh, N.D., Gangavarapu, K., Quick, J., Matteson, N.L., De Jesus, J.G., Main, B.J., Tan, A.L., Paul, L.M., Brackney, D.E., Grewal, S., Gurfield, N., Van Rompay, K.K.A., Isern, S., Michael, S.F., Coffey, L.L., Loman, N.J., and Andersen, K.G. (2019). An amplicon-based sequencing framework for accurately measuring intrahost virus diversity using PrimalSeq and iVar. *Genome Biol* 20**,** 8.

Guminska, N., Plecha, M., Zakrys, B., and Milanowski, R. (2018). Order of removal of conventional and nonconventional introns from nuclear transcripts of Euglena gracilis. *PLoS Genet* 14**,** e1007761.

Hargreaves, A.D., and Mulley, J.F. (2015). Assessing the utility of the Oxford Nanopore MinION for snake venom gland cDNA sequencing. *PeerJ* 3**,** e1441.

Herzel, L., Straube, K., and Neugebauer, K.M. (2018). Long-read sequencing of nascent RNA reveals coupling among RNA processing events. *Genome Res* 28**,** 1008-1019.

Hillman, R.T., Green, R.E., and Brenner, S.E. (2004). An unappreciated role for RNA surveillance. *Genome Biol* 5**,** R8.

Hoang, N.V., Furtado, A., Mason, P.J., Marquardt, A., Kasirajan, L., Thirugnanasambandam, P.P., Botha, F.C., and Henry, R.J. (2017). A survey of the complex transcriptome from the highly polyploid sugarcane genome using full-length isoform sequencing and de novo assembly from short read sequencing. *BMC Genomics* 18**,** 395.

Ibrahim, F., Maragkakis, M., Alexiou, P., and Mourelatos, Z. (2018). Ribothrypsis, a novel process of canonical mRNA decay, mediates ribosome-phased mRNA endonucleolysis. *Nat Struct Mol Biol* 25**,** 302-310.

Jaworski, E., and Routh, A. (2017). Parallel ClickSeq and Nanopore sequencing elucidates the rapid evolution of defective-interfering RNAs in Flock House virus. *PLoS Pathog* 13**,** e1006365.

Jeck, W.R., Lee, J., Robinson, H., Le, L.P., Iafrate, A.J., and Nardi, V. (2019). A Nanopore Sequencing-Based Assay for Rapid Detection of Gene Fusions. *J Mol Diagn* 21**,** 58-69.

Jenjaroenpun, P., Wongsurawat, T., Pereira, R., Patumcharoenpol, P., Ussery, D.W., Nielsen, J., and Nookaew, I. (2018). Complete genomic and transcriptional landscape analysis using third-

generation sequencing: a case study of Saccharomyces cerevisiae CEN.PK113-7D. *Nucleic Acids Res* 46**,** e38.
Jiang, X., Hall, A.B., Biedler, J.K., and Tu, Z. (2017). Single molecule RNA sequencing uncovers trans-splicing and improves annotations in Anopheles stephensi. *Insect Mol Biol* 26**,** 298-307.
Jo, I.H., Lee, J., Hong, C.E., Lee, D.J., Bae, W., Park, S.G., Ahn, Y.J., Kim, Y.C., Kim, J.U., Lee, J.W., Hyun, D.Y., Rhee, S.K., Hong, C.P., Bang, K.H., and Ryu, H. (2017). Isoform Sequencing Provides a More Comprehensive View of the Panax ginseng Transcriptome. *Genes (Basel)* 8.
Kafetzopoulou, L.E., Efthymiadis, K., Lewandowski, K., Crook, A., Carter, D., Osborne, J., Aarons, E., Hewson, R., Hiscox, J.A., Carroll, M.W., Vipond, R., and Pullan, S.T. (2018). Assessment of metagenomic Nanopore and Illumina sequencing for recovering whole genome sequences of chikungunya and dengue viruses directly from clinical samples. *Euro Surveill* 23.
Karl, J.A., Graham, M.E., Wiseman, R.W., Heimbruch, K.E., Gieger, S.M., Doxiadis, G.G., Bontrop, R.E., and O'connor, D.H. (2017). Major histocompatibility complex haplotyping and long-amplicon allele discovery in cynomolgus macaques from Chinese breeding facilities. *Immunogenetics* 69**,** 211-229.
Kilianski, A., Roth, P.A., Liem, A.T., Hill, J.M., Willis, K.L., Rossmaier, R.D., Marinich, A.V., Maughan, M.N., Karavis, M.A., Kuhn, J.H., Honko, A.N., and Rosenzweig, C.N. (2016). Use of Unamplified RNA/cDNA-Hybrid Nanopore Sequencing for Rapid Detection and Characterization of RNA Viruses. *Emerg Infect Dis* 22**,** 1448-1451.
Kim, J.A., Roy, N.S., Lee, I.H., Choi, A.Y., Choi, B.S., Yu, Y.S., Park, N.I., Park, K.C., Kim, S., Yang, H.S., and Choi, I.Y. (2018a). Genome-wide transcriptome profiling of the medicinal plant Zanthoxylum planispinum using a single-molecule direct RNA sequencing approach. *Genomics*.
Kim, J.Y., Lim, H.Y., Shin, S.E., Cha, H.K., Seo, J.H., Kim, S.K., Park, S.H., and Son, G.H. (2018b). Comprehensive transcriptome analysis of Sarcophaga peregrina, a forensically important fly species. *Sci Data* 5**,** 180220.
Kim, M.A., Rhee, J.S., Kim, T.H., Lee, J.S., Choi, A.Y., Choi, B.S., Choi, I.Y., and Sohn, Y.C. (2017). Alternative Splicing Profile and Sex-Preferential Gene Expression in the Female and Male Pacific Abalone Haliotis discus hannai. *Genes (Basel)* 8.
Kovar, L., Nageswara-Rao, M., Ortega-Rodriguez, S., Dugas, D.V., Straub, S., Cronn, R., Strickler, S.R., Hughes, C.E., Hanley, K.A., Rodriguez, D.N., Langhorst, B.W., Dimalanta, E.T., and Bailey, C.D. (2018). PacBio-Based Mitochondrial Genome Assembly of Leucaena trichandra (Leguminosae) and an Intrageneric Assessment of Mitochondrial RNA Editing. *Genome Biol Evol* 10**,** 2501-2517.
Kumar-Sinha, C., Kalyana-Sundaram, S., and Chinnaiyan, A.M. (2015). Landscape of gene fusions in epithelial cancers: seq and ye shall find. *Genome Med* 7**,** 129.
Kuo, R.I., Tseng, E., Eory, L., Paton, I.R., Archibald, A.L., and Burt, D.W. (2017). Normalized long read RNA sequencing in chicken reveals transcriptome complexity similar to human. *BMC Genomics* 18**,** 323.
Lagarde, J., Uszczynska-Ratajczak, B., Carbonell, S., Perez-Lluch, S., Abad, A., Davis, C., Gingeras, T.R., Frankish, A., Harrow, J., Guigo, R., and Johnson, R. (2017). High-throughput annotation of full-length long noncoding RNAs with capture long-read sequencing. *Nat Genet* 49**,** 1731-1740.
Lagarde, J., Uszczynska-Ratajczak, B., Santoyo-Lopez, J., Gonzalez, J.M., Tapanari, E., Mudge, J.M., Steward, C.A., Wilming, L., Tanzer, A., Howald, C., Chrast, J., Vela-Boza, A., Rueda, A., Lopez-Domingo, F.J., Dopazo, J., Reymond, A., Guigo, R., and Harrow, J. (2016). Extension of human lncRNA transcripts by RACE coupled with long-read high-throughput sequencing (RACE-Seq). *Nat Commun* 7**,** 12339.
Lea, W.A., Parnell, S.C., Wallace, D.P., Calvet, J.P., Zelenchuk, L.V., Alvarez, N.S., and Ward, C.J. (2018). Human-Specific Abnormal Alternative Splicing of Wild-Type PKD1 Induces Premature Termination of Polycystin-1. *J Am Soc Nephrol* 29**,** 2482-2492.

Lee, M., Lee, K., Yu, N., Jang, I., Choi, I., Kim, P., Jang, Y.E., Kim, B., Kim, S., Lee, B., Kang, J., and Lee, S. (2017). ChimerDB 3.0: an enhanced database for fusion genes from cancer transcriptome and literature data mining. *Nucleic Acids Res* 45**,** D784-D789.

Li, A., Zhang, J., and Zhou, Z. (2014). PLEK: a tool for predicting long non-coding RNAs and messenger RNAs based on an improved k-mer scheme. *BMC Bioinformatics* 15**,** 311.

Li, C., Chng, K.R., Boey, E.J., Ng, A.H., Wilm, A., and Nagarajan, N. (2016). INC-Seq: accurate single molecule reads using nanopore sequencing. *Gigascience* 5**,** 34.

Li, J., Harata-Lee, Y., Denton, M.D., Feng, Q., Rathjen, J.R., Qu, Z., and Adelson, D.L. (2017). Long read reference genome-free reconstruction of a full-length transcriptome from Astragalus membranaceus reveals transcript variants involved in bioactive compound biosynthesis. *Cell Discov* 3**,** 17031.

Liu, H., Smith, T.P.L., Nonneman, D.J., Dekkers, J.C.M., and Tuggle, C.K. (2017a). A high-quality annotated transcriptome of swine peripheral blood. *BMC Genomics* 18**,** 479.

Liu, X., Mei, W., Soltis, P.S., Soltis, D.E., and Barbazuk, W.B. (2017b). Detecting alternatively spliced transcript isoforms from single-molecule long-read sequences without a reference genome. *Mol Ecol Resour* 17**,** 1243-1256.

Luo, D., Zhou, Q., Wu, Y., Chai, X., Liu, W., Wang, Y., Yang, Q., Wang, Z., and Liu, Z. (2019). Full-length transcript sequencing and comparative transcriptomic analysis to evaluate the contribution of osmotic and ionic stress components towards salinity tolerance in the roots of cultivated alfalfa (Medicago sativa L.). *BMC Plant Biol* 19**,** 32.

Magrini, V., Gao, X., Rosa, B.A., Mcgrath, S., Zhang, X., Hallsworth-Pepin, K., Martin, J., Hawdon, J., Wilson, R.K., and Mitreva, M. (2018). Improving eukaryotic genome annotation using single molecule mRNA sequencing. *BMC Genomics* 19**,** 172.

Maher, C.A., Kumar-Sinha, C., Cao, X., Kalyana-Sundaram, S., Han, B., Jing, X., Sam, L., Barrette, T., Palanisamy, N., and Chinnaiyan, A.M. (2009). Transcriptome sequencing to detect gene fusions in cancer. *Nature* 458**,** 97-101.

Mccabe, M., Cormican, P., Johnston, D., and Earley, B. (2018). Simultaneous detection of DNA and RNA virus species involved in bovine respiratory disease by PCR-free rapid tagmentation-based library preparation and MinION nanopore sequencing. *bioRxiv***,** 269936.

Minoche, A.E., Dohm, J.C., Schneider, J., Holtgrawe, D., Viehover, P., Montfort, M., Sorensen, T.R., Weisshaar, B., and Himmelbauer, H. (2015). Exploiting single-molecule transcript sequencing for eukaryotic gene prediction. *Genome Biol* 16**,** 184.

Moldovan, N., Balazs, Z., Tombacz, D., Csabai, Z., Szucs, A., Snyder, M., and Boldogkoi, Z. (2017a). Multi-platform analysis reveals a complex transcriptome architecture of a circovirus. *Virus Res* 237**,** 37-46.

Moldovan, N., Szucs, A., Tombacz, D., Balazs, Z., Csabai, Z., Snyder, M., and Boldogkoi, Z. (2018a). Multiplatform next-generation sequencing identifies novel RNA molecules and transcript isoforms of the endogenous retrovirus isolated from cultured cells. *FEMS Microbiol Lett* 365.

Moldovan, N., Tombacz, D., Szucs, A., Csabai, Z., Balazs, Z., Kis, E., Molnar, J., and Boldogkoi, Z. (2018b). Third-generation Sequencing Reveals Extensive Polycistronism and Transcriptional Overlapping in a Baculovirus. *Sci Rep* 8**,** 8604.

Moldovan, N., Tombacz, D., Szucs, A., Csabai, Z., Snyder, M., and Boldogkoi, Z. (2017b). Multi-Platform Sequencing Approach Reveals a Novel Transcriptome Profile in Pseudorabies Virus. *Front Microbiol* 8**,** 2708.

Nattestad, M., Goodwin, S., Ng, K., Baslan, T., Sedlazeck, F.J., Rescheneder, P., Garvin, T., Fang, H., Gurtowski, J., Hutton, E., Tseng, E., Chin, C.S., Beck, T., Sundaravadanam, Y., Kramer, M., Antoniou, E., Mcpherson, J.D., Hicks, J., Mccombie, W.R., and Schatz, M.C. (2018). Complex rearrangements and oncogene amplifications revealed by long-read DNA and RNA sequencing of a breast cancer cell line. *Genome Res* 28**,** 1126-1135.

Nicholls, S.M., Aubrey, W., Edwards, A., De Grave, K., Huws, S., Schietgat, L., Soares, A., Creevey, C.J., and Clare, A. (2017). Computational haplotype recovery and long-read validation identifies novel isoforms of industrially relevant enzymes from natural microbial communities. *bioRxiv*, 223404.

Ning, G., Cheng, X., Luo, P., Liang, F., Wang, Z., Yu, G., Li, X., Wang, D., and Bao, M. (2017). Hybrid sequencing and map finding (HySeMaFi): optional strategies for extensively deciphering gene splicing and expression in organisms without reference genome. *Sci Rep* 7, 43793.

Nowell, P.C., and Hungerford, D.A. (1961). Chromosome studies in human leukemia. II. Chronic granulocytic leukemia. *J Natl Cancer Inst* 27, 1013-1035.

Oesterreich, F.C., Herzel, L., Straube, K., Hujer, K., Howard, J., and Neugebauer, K.M. (2016). Splicing of Nascent RNA Coincides with Intron Exit from RNA Polymerase II. *Cell* 165, 372-381.

Oh, J., Shin, Y., Ha, I.J., Lee, M.Y., Lee, S.G., Kang, B.C., Kyeong, D., and Kim, D. (2018). Transcriptome Profiling of Two Ornamental and Medicinal Papaver Herbs. *Int J Mol Sci* 19.

Pacbio_Website _1 (2013). https://www.pacb.com/uncategorized/a-closer-look-at-accuracy-in-pacbio/.

Pacbio_Website _2 (2015). https://www.pacb.com/wp-content/uploads/2015/09/Perspective_UnderstandingAccuracySMRTSequencing.pdf.

Park, S.G., Yoo, S.I., Ryu, D.S., Lee, H., Ahn, Y.J., Ryu, H., Ko, J., and Hong, C.P. (2017). Long-read transcriptome data for improved gene prediction in Lentinula edodes. *Data Brief* 15, 454-458.

Parker, B.C., and Zhang, W. (2013). Fusion genes in solid tumors: an emerging target for cancer diagnosis and treatment. *Chin J Cancer* 32, 594-603.

Peach, S.E., York, K., and Hesselberth, J.R. (2015). Global analysis of RNA cleavage by 5'-hydroxyl RNA sequencing. *Nucleic Acids Res* 43, e108.

Piriyapongsa, J., Kaewprommal, P., Vaiwsri, S., Anuntakarun, S., Wirojsirasak, W., Punpee, P., Klomsa-Ard, P., Shaw, P.J., Pootakham, W., Yoocha, T., Sangsrakru, D., Tangphatsornruang, S., Tongsima, S., and Tragoonrung, S. (2018). Uncovering full-length transcript isoforms of sugarcane cultivar Khon Kaen 3 using single-molecule long-read sequencing. *PeerJ* 6, e5818.

Prall, T.M., Graham, M.E., Karl, J.A., Wiseman, R.W., Ericsen, A.J., Raveendran, M., Alan Harris, R., Muzny, D.M., Gibbs, R.A., Rogers, J., and O'connor, D.H. (2017). Improved full-length killer cell immunoglobulin-like receptor transcript discovery in Mauritian cynomolgus macaques. *Immunogenetics* 69, 325-339.

Prazsak, I., Moldovan, N., Balazs, Z., Tombacz, D., Megyeri, K., Szucs, A., Csabai, Z., and Boldogkoi, Z. (2018). Long-read sequencing uncovers a complex transcriptome topology in varicella zoster virus. *BMC Genomics* 19, 873.

Pretto, D.I., Eid, J.S., Yrigollen, C.M., Tang, H.T., Loomis, E.W., Raske, C., Durbin-Johnson, B., Hagerman, P.J., and Tassone, F. (2015). Differential increases of specific FMR1 mRNA isoforms in premutation carriers. *J Med Genet* 52, 42-52.

Quick, J., Grubaugh, N.D., Pullan, S.T., Claro, I.M., Smith, A.D., Gangavarapu, K., Oliveira, G., Robles-Sikisaka, R., Rogers, T.F., Beutler, N.A., Burton, D.R., Lewis-Ximenez, L.L., De Jesus, J.G., Giovanetti, M., Hill, S.C., Black, A., Bedford, T., Carroll, M.W., Nunes, M., Alcantara, L.C., Jr., Sabino, E.C., Baylis, S.A., Faria, N.R., Loose, M., Simpson, J.T., Pybus, O.G., Andersen, K.G., and Loman, N.J. (2017). Multiplex PCR method for MinION and Illumina sequencing of Zika and other virus genomes directly from clinical samples. *Nat Protoc* 12, 1261-1276.

Raj, B., and Blencowe, B.J. (2015). Alternative Splicing in the Mammalian Nervous System: Recent Insights into Mechanisms and Functional Roles. *Neuron* 87, 14-27.

Russell, J.A., Campos, B., Stone, J., Blosser, E.M., Burkett-Cadena, N., and Jacobs, J.L. (2018). Unbiased Strain-Typing of Arbovirus Directly from Mosquitoes Using Nanopore Sequencing: A Field-forward Biosurveillance Protocol. *Sci Rep* 8, 5417.

Sahlin, K., Tomaszkiewicz, M., Makova, K.D., and Medvedev, P. (2018). Deciphering highly similar multigene family transcripts from Iso-Seq data with IsoCon. *Nat Commun* 9, 4601.

Salimullah, M., Sakai, M., Plessy, C., and Carninci, P. (2011). NanoCAGE: a high-resolution technique to discover and interrogate cell transcriptomes. *Cold Spring Harb Protoc* 2011, pdb prot5559.

Schoenberg, D.R., and Maquat, L.E. (2012). Regulation of cytoplasmic mRNA decay. *Nat Rev Genet* 13**,** 246-259.

Schreiner, D., Nguyen, T.M., Russo, G., Heber, S., Patrignani, A., Ahrne, E., and Scheiffele, P. (2014). Targeted combinatorial alternative splicing generates brain region-specific repertoires of neurexins. *Neuron* 84**,** 386-398.

Schultz, D.T., Kotlobay, A.A., Ziganshin, R., Bannikov, A., Markina, N.M., Chepurnyh, T.V., Shakhova, E.S., Palkina, K., Haddock, S.H.D., Yampolsky, I.V., and Oba, Y. (2018). Luciferase of the Japanese syllid polychaete Odontosyllis undecimdonta. *Biochem Biophys Res Commun* 502**,** 318-323.

Sharon, D., Tilgner, H., Grubert, F., and Snyder, M. (2013). A single-molecule long-read survey of the human transcriptome. *Nat Biotechnol* 31**,** 1009-1014.

Shepard, P.J., Choi, E.A., Lu, J., Flanagan, L.A., Hertel, K.J., and Shi, Y. (2011). Complex and dynamic landscape of RNA polyadenylation revealed by PAS-Seq. *RNA* 17**,** 761-772.

Sherbenou, D.W., Hantschel, O., Kaupe, I., Willis, S., Bumm, T., Turaga, L.P., Lange, T., Dao, K.H., Press, R.D., Druker, B.J., Superti-Furga, G., and Deininger, M.W. (2010). BCR-ABL SH3-SH2 domain mutations in chronic myeloid leukemia patients on imatinib. *Blood* 116**,** 3278-3285.

Steijger, T., Abril, J.F., Engstrom, P.G., Kokocinski, F., Hubbard, T.J., Guigo, R., Harrow, J., and Bertone, P. (2013). Assessment of transcript reconstruction methods for RNA-seq. *Nat Methods* 10**,** 1177-1184.

Sun, W., Gao, Q., Schaefke, B., Hu, Y., and Chen, W. (2018). Pervasive allele-specific regulation on RNA decay in hybrid mice. *Life Science Alliance* 1.

Suzuki, A., Suzuki, M., Mizushima-Sugano, J., Frith, M.C., Makalowski, W., Kohno, T., Sugano, S., Tsuchihara, K., and Suzuki, Y. (2017). Sequencing and phasing cancer mutations in lung cancers using a long-read portable sequencer. *DNA Res* 24**,** 585-596.

Tang, A.D., Soulette, C.M., Van Baren, M.J., Hart, K., Hrabeta-Robinson, E., Wu, C.J., and Brooks, A.N. (2018). Full-length transcript characterization of SF3B1 mutation in chronic lymphocytic leukemia reveals downregulation of retained introns. *bioRxiv***,** 410183.

Tardaguila, M., De La Fuente, L., Marti, C., Pereira, C., Pardo-Palacios, F.J., Del Risco, H., Ferrell, M., Mellado, M., Macchietto, M., Verheggen, K., Edelmann, M., Ezkurdia, I., Vazquez, J., Tress, M., Mortazavi, A., Martens, L., Rodriguez-Navarro, S., Moreno-Manzano, V., and Conesa, A. (2018). SQANTI: extensive characterization of long-read transcript sequences for quality control in full-length transcriptome identification and quantification. *Genome Res*.

Tilgner, H., Grubert, F., Sharon, D., and Snyder, M.P. (2014). Defining a personal, allele-specific, and single-molecule long-read transcriptome. *Proc Natl Acad Sci U S A* 111**,** 9869-9874.

Tombacz, D., Balazs, Z., Csabai, Z., Moldovan, N., Szucs, A., Sharon, D., Snyder, M., and Boldogkoi, Z. (2017). Characterization of the Dynamic Transcriptome of a Herpesvirus with Long-read Single Molecule Real-Time Sequencing. *Sci Rep* 7**,** 43751.

Tombacz, D., Csabai, Z., Olah, P., Balazs, Z., Liko, I., Zsigmond, L., Sharon, D., Snyder, M., and Boldogkoi, Z. (2016). Full-Length Isoform Sequencing Reveals Novel Transcripts and Substantial Transcriptional Overlaps in a Herpesvirus. *PLoS One* 11**,** e0162868.

Tombacz, D., Csabai, Z., Olah, P., Havelda, Z., Sharon, D., Snyder, M., and Boldogkoi, Z. (2015). Characterization of novel transcripts in pseudorabies virus. *Viruses* 7**,** 2727-2744.

Tombacz, D., Prazsak, I., Moldovan, N., Szucs, A., and Boldogkoi, Z. (2018a). Lytic Transcriptome Dataset of Varicella Zoster Virus Generated by Long-Read Sequencing. *Front Genet* 9**,** 460.

Tombacz, D., Prazsak, I., Szucs, A., Denes, B., Snyder, M., and Boldogkoi, Z. (2018b). Dynamic transcriptome profiling dataset of vaccinia virus obtained from long-read sequencing techniques. *Gigascience* 7.

Tombácz, D., Sharon, D., Szűcs, A., Moldován, N., Snyder, M., and Boldogkői, Z. (2018). Transcriptome-wide survey of pseudorabies virus using next- and third-generation sequencing platforms. *Scientific Data* 5**,** 180119.

Trapnell, C., Williams, B.A., Pertea, G., Mortazavi, A., Kwan, G., Van Baren, M.J., Salzberg, S.L., Wold, B.J., and Pachter, L. (2010). Transcript assembly and quantification by RNA-Seq reveals unannotated transcripts and isoform switching during cell differentiation. *Nat Biotechnol* 28**,** 511-515.

Treutlein, B., Gokce, O., Quake, S.R., and Sudhof, T.C. (2014). Cartography of neurexin alternative splicing mapped by single-molecule long-read mRNA sequencing. *Proc Natl Acad Sci U S A* 111**,** E1291-1299.

Volden, R., Palmer, T., Byrne, A., Cole, C., Schmitz, R.J., Green, R.E., and Vollmers, C. (2018). Improving nanopore read accuracy with the R2C2 method enables the sequencing of highly multiplexed full-length single-cell cDNA. *Proc Natl Acad Sci U S A*.

Wang, B., Tseng, E., Regulski, M., Clark, T.A., Hon, T., Jiao, Y., Lu, Z., Olson, A., Stein, J.C., and Ware, D. (2016). Unveiling the complexity of the maize transcriptome by single-molecule long-read sequencing. *Nat Commun* 7**,** 11708.

Wang, J.R., Holt, J., Mcmillan, L., and Jones, C.D. (2018). FMLRC: Hybrid long read error correction using an FM-index. *BMC Bioinformatics* 19**,** 50.

Wang, L., Park, H.J., Dasari, S., Wang, S., Kocher, J.P., and Li, W. (2013). CPAT: Coding-Potential Assessment Tool using an alignment-free logistic regression model. *Nucleic Acids Res* 41**,** e74.

Wang, T., Wang, H., Cai, D., Gao, Y., Zhang, H., Wang, Y., Lin, C., Ma, L., and Gu, L. (2017). Comprehensive profiling of rhizome-associated alternative splicing and alternative polyadenylation in moso bamboo (Phyllostachys edulis). *Plant J* 91**,** 684-699.

Wen, M., Ng, J.H.J., Zhu, F., Chionh, Y.T., Chia, W.N., Mendenhall, I.H., Lee, B.P., Irving, A.T., and Wang, L.F. (2018). Exploring the genome and transcriptome of the cave nectar bat Eonycteris spelaea with PacBio long-read sequencing. *Gigascience* 7.

Westbrook, C.J., Karl, J.A., Wiseman, R.W., Mate, S., Koroleva, G., Garcia, K., Sanchez-Lockhart, M., O'connor, D.H., and Palacios, G. (2015). No assembly required: Full-length MHC class I allele discovery by PacBio circular consensus sequencing. *Hum Immunol* 76**,** 891-896.

Workman, R.E., Myrka, A.M., Wong, G.W., Tseng, E., Welch, K.C., Jr., and Timp, W. (2018a). Single-molecule, full-length transcript sequencing provides insight into the extreme metabolism of the ruby-throated hummingbird Archilochus colubris. *Gigascience* 7**,** 1-12.

Workman, R.E., Tang, A., Tang, P.S., Jain, M., Tyson, J.R., Zuzarte, P.C., Gilpatrick, T., Razaghi, R., Quick, J., Sadowski, N., Holmes, N., Goes De Jesus, J., Jones, K., Snutch, T.P., Loman, N.J., Paten, B., Loose, M.W., Simpson, J.T., Olsen, H.E., Brooks, A.N., Akeson, M., and Timp, W. (2018b). Nanopore native RNA sequencing of a human poly(A) transcriptome. *bioRxiv***,** 459529.

Wyman, D., and Mortazavi, A. (2019). TranscriptClean: variant-aware correction of indels, mismatches and splice junctions in long-read transcripts. *Bioinformatics* 35**,** 340-342.

Xie, S.Q., Han, Y., Chen, X.Z., Cao, T.Y., Ji, K.K., Zhu, J., Ling, P., and Xiao, C.L. (2018). ISOdb: A Comprehensive Database of Full-Length Isoforms Generated by Iso-Seq. *Int J Genomics* 2018**,** 9207637.

Xue, X.Y., Majerciak, V., Uberoi, A., Kim, B.H., Gotte, D., Chen, X., Cam, M., Lambert, P.F., and Zheng, Z.M. (2017). The full transcription map of mouse papillomavirus type 1 (MmuPV1) in mouse wart tissues. *PLoS Pathog* 13**,** e1006715.

Yan, B., Boitano, M., Clark, T.A., and Ettwiller, L. (2018). SMRT-Cappable-seq reveals complex operon variants in bacteria. *Nat Commun* 9**,** 3676.

Yang, Y., Zhan, L., Zhang, W., Sun, F., Wang, W., Tian, N., Bi, J., Wang, H., Shi, D., Jiang, Y., Zhang, Y., and Jin, Y. (2011). RNA secondary structure in mutually exclusive splicing. *Nat Struct Mol Biol* 18**,** 159-168.

Yi, S., Zhou, X., Li, J., Zhang, M., and Luo, S. (2018). Full-length transcriptome of Misgurnus anguillicaudatus provides insights into evolution of genus Misgurnus. *Sci Rep* 8**,** 11699.

Zabriskie, M.S., Eide, C.A., Tantravahi, S.K., Vellore, N.A., Estrada, J., Nicolini, F.E., Khoury, H.J., Larson, R.A., Konopleva, M., Cortes, J.E., Kantarjian, H., Jabbour, E.J., Kornblau, S.M., Lipton, J.H., Rea, D., Stenke, L., Barbany, G., Lange, T., Hernandez-Boluda, J.C., Ossenkoppele, G.J., Press, R.D.,

Chuah, C., Goldberg, S.L., Wetzler, M., Mahon, F.X., Etienne, G., Baccarani, M., Soverini, S., Rosti, G., Rousselot, P., Friedman, R., Deininger, M., Reynolds, K.R., Heaton, W.L., Eiring, A.M., Pomicter, A.D., Khorashad, J.S., Kelley, T.W., Baron, R., Druker, B.J., Deininger, M.W., and O'hare, T. (2014). BCR-ABL1 compound mutations combining key kinase domain positions confer clinical resistance to ponatinib in Ph chromosome-positive leukemia. *Cancer Cell* 26**,** 428-442.

Zhang, G., Sun, M., Wang, J., Lei, M., Li, C., Zhao, D., Huang, J., Li, W., Li, S., Li, J., Yang, J., Luo, Y., Hu, S., and Zhang, B. (2019a). PacBio full-length cDNA sequencing integrated with RNA-seq reads drastically improves the discovery of splicing transcripts in rice. *Plant J* 97**,** 296-305.

Zhang, H., Wang, H., Zhu, Q., Gao, Y., Wang, H., Zhao, L., Wang, Y., Xi, F., Wang, W., Yang, Y., Lin, C., and Gu, L. (2018). Transcriptome characterization of moso bamboo (Phyllostachys edulis) seedlings in response to exogenous gibberellin applications. *BMC Plant Biol* 18**,** 125.

Zhang, S.J., Wang, C., Yan, S., Fu, A., Luan, X., Li, Y., Sunny Shen, Q., Zhong, X., Chen, J.Y., Wang, X., Chin-Ming Tan, B., He, A., and Li, C.Y. (2017). Isoform Evolution in Primates through Independent Combination of Alternative RNA Processing Events. *Mol Biol Evol* 34**,** 2453-2468.

Zhang, W., Ciclitira, P., and Messing, J. (2014). PacBio sequencing of gene families - a case study with wheat gluten genes. *Gene* 533**,** 541-546.

Zhang, X., Li, G., Jiang, H., Li, L., Ma, J., Li, H., and Chen, J. (2019b). Full-length transcriptome analysis of Litopenaeus vannamei reveals transcript variants involved in the innate immune system. *Fish Shellfish Immunol* 87**,** 346-359.

Zhu, F.Y., Chen, M.X., Ye, N.H., Shi, L., Ma, K.L., Yang, J.F., Cao, Y.Y., Zhang, Y., Yoshida, T., Fernie, A.R., Fan, G.Y., Wen, B., Zhou, R., Liu, T.Y., Fan, T., Gao, B., Zhang, D., Hao, G.F., Xiao, S., Liu, Y.G., and Zhang, J. (2017). Proteogenomic analysis reveals alternative splicing and translation as part of the abscisic acid response in Arabidopsis seedlings. *Plant J* 91**,** 518-533.

Zulkapli, M.M., Rosli, M.a.F., Salleh, F.I.M., Mohd Noor, N., Aizat, W.M., and Goh, H.H. (2017). Iso-Seq analysis of Nepenthes ampullaria, Nepenthes rafflesiana and Nepenthes x hookeriana for hybridisation study in pitcher plants. *Genom Data* 12**,** 130-131.

Zuo, C., Blow, M., Sreedasyam, A., Kuo, R.C., Ramamoorthy, G.K., Torres-Jerez, I., Li, G., Wang, M., Dilworth, D., Barry, K., Udvardi, M., Schmutz, J., Tang, Y., and Xu, Y. (2018). Revealing the transcriptomic complexity of switchgrass by PacBio long-read sequencing. *Biotechnol Biofuels* 11**,** 170.
